# Causal association between neutrophil extracellular traps and rheumatoid arthritis: A 2-sample bidirectional Mendelian randomization study

**DOI:** 10.1097/MD.0000000000045331

**Published:** 2025-10-31

**Authors:** Junwei Luo, Xinlong Li, Xi Wang, Jiaqi Yuan

**Affiliations:** a Department of Anesthesiology, Sir Run Run Shaw Hospital, School of Medicine, Zhejiang University, Hangzhou, Zhejiang Province, China.

**Keywords:** causality, interleukins, Mendelian randomization, neutrophil extracellular traps, rheumatoid arthritis

## Abstract

This study explores the causal association between neutrophil extracellular traps and rheumatoid arthritis (RA). A 2-sample bidirectional Mendelian randomization (MR) analysis was performed. The primary analyses were performed using the inverse variance weighted method, with confirmation using the weighted median, weighted mode, and MR-Egger methods. Heterogeneity was detected using Cochran’s *Q*-test, pleiotropy using MR-Egger regression, and outliers using Mendelian randomization pleiotropy residual sum and outlier. The leave-one-out method was used to determine whether a single single-nucleotide polymorphism drove the results. Using RA as the outcome, a causal link emerged between neutrophil count and RA. Notably, substantial heterogeneity and the detection of outliers for neutrophil count led to the attenuation of this causal association post-correction, but the weighted median and weighted mode analyses hinted at a potential causal relationship. Causal effects of RA were seen on neutrophil count, tumor necrosis factor-α, IL-5, IL-13, and myeloperoxidase. Mendelian randomization pleiotropy residual sum and outlier identified outliers in neutrophil count, yet the causal link persisted following correction. No weak instrumental bias was observed. No horizontal pleiotropy was observed. The leave-one-out analysis showed that no single single-nucleotide polymorphism was driving the results. Neutrophil extracellular traps had no causal associations on RA, but RA had causal associations on neutrophil count, tumor necrosis factor-α levels, IL-5, IL-13, and myeloperoxidase.

## 1. Introduction

Rheumatoid arthritis (RA) is a systemic inflammatory disease characterized by symmetric, relapsing, or chronic destructive synovitis. There may also be multisystem involvement.^[1]^ The global prevalence of RA is 0.5% to 1%.^[1]^ RA is more common in females than males, with usual onset between the ages of 30 and 60 years.^[1]^ Characteristically, RA involves 3 or more proximal interphalangeal joints, metacarpophalangeal joints, wrist, and metatarsophalangeal joints, although other joints may be involved as well. The eventual, classic presentation is symmetric polyarthritis, but in the early stages, it can present as oligoarthritis or, less commonly, as recurrent monoarthritis. The most common complication of RA is musculoskeletal disability from destructive arthritis. The common concomitant comorbid conditions in patients with RA include osteoporosis, increased cardiovascular mortality, and Sicca symptoms of the eyes and mouth.^[2]^ The most prominent risk factor for the development of RA is genetic.^[1]^ A family history of RA is associated with an increased risk for RA. First degree relatives of patients with RA are reported to have a 2 to 5 times higher risk of developing the disease. Familial aggregation in population-based studies was reported to have an estimated risk of 3.6 to 4.6 for siblings and an estimated risk of 2.7 to 3.9 for parents. Twin studies have reported inconsistent results indicating an interaction of genetic and environmental factors.^[3]^

Discovered in 2004, neutrophil extracellular traps (NETs) are an antimicrobial mechanism found on neutrophils. NETs are composed of extracellular DNA and antimicrobial proteins. The formation of NETs involves either suicidal NETosis, which involves neutrophil death, or vital NETosis, in which the neutrophil remains alive. Still, the signaling and molecular events leading to NETosis remain elusive, but several cytokines are involved, including interleukin (IL)-1β, IL-4, IL-5, IL-6, IL-18, IL-13, tumor necrosis factor (TNF)-α, blood myeloperoxidase (MPO), and blood MPO-DNA complexes.^[4]^ NETs play a crucial role in the immune system by entrapping microbes.^[4]^ The number of neutrophils will influence the NET levels.^[4]^

On the other hand, despite their essential roles in immunity, recent research suggests that an increase in NETs is a factor in the onset of autoimmune diseases, such as RA,^[5,6]^ autoimmune small-vessel vasculitis, and systemic lupus erythematosus.^[7]^ NET formation appears to involve oxidative stress, which is common in autoimmune diseases like RA.^[8]^ In addition, animal experiments showed that NETs drive the pathogenesis of RA and that NETs could be used for the diagnosis of RA.^[9]^ NETs appear to act as inflammatory mediators that promote RA-related bone resorption^[10]^ and interstitial lung disease associated with RA.^[11]^ Many molecules released during NET production (e.g., histones, citrulline peptides, and MPO) are targeted autoantigens in autoimmune diseases, suggesting that NETs have a direct causal relationship between the production of autoantigens and autoimmune diseases, including RA,^[12]^ and that such autoantibodies could be used for RA diagnosis.^[13]^ There is a vicious circle between NETs and fibroblast-like synovial cells mediated by IL-33.^[14]^ NETs have been observed in patients with RA flares, with diagnosis and treatment potentials.^[15]^ In addition, NETs mediate bone erosion in RA by enhancing RANKL-induced osteoclastogenesis^[16]^ and induce fibroblast-like synovial cell pyroptosis via the NF-κB/Caspase 3/GSDME signaling pathway.^[17]^ Therefore, there is an accumulation of compelling evidence linking NETs to RA,^[18]^ but studies on the association between NETs and autoimmune diseases have yielded inconsistent results. Traditionally, genetics is considered a major factor in the onset of autoimmune diseases.^[19]^ In addition, the exact causal relationship between RA and NETs remains uncertain, that is, that RA leads to an increase in NETs and NETs exacerbate disease complications.^[6,15,20,21]^ Therefore, the present study examined the causal relationship between RA and NETs through Mendelian randomization (MR).

MR is based on the common genetic variations for different environmental exposures and allows the exploration of possible causal associations between exposures and diseases.^[22]^ MR studies use data from large-scale genome-wide association studies (GWASs), which provide results on millions of single-nucleotide polymorphisms (SNPs) and their association with various phenotypes and diseases.^[23]^ GWASs led to a revolution in genetic medicine and the understanding of complex traits and diseases.^[23]^ Two-sample MR uses the associations between SNPs and exposure and between SNPs and outcomes from different GWASs to combine them into a causality estimation. Under key assumptions, MR reduces reverse causation and confounding, which often substantially impede or mislead the interpretation of results from epidemiological studies.^[24]^ Bidirectional MR examines the causal associations in 2 directions, first for factors on disease and then for the disease on the factor, allowing a better understanding of the causal associations.^[25]^

Therefore, this MR study aimed to explore the bidirectional causal associations between genetically predicted NETs and RA. The results could help understand the pathophysiology of RA in relation to NETs, that is, NETs are involved in the onset of RA, or RA leads to increased NETs that participate in exacerbation.

## 2. Materials and methods

### 2.1. Study design

**Figure 1.**
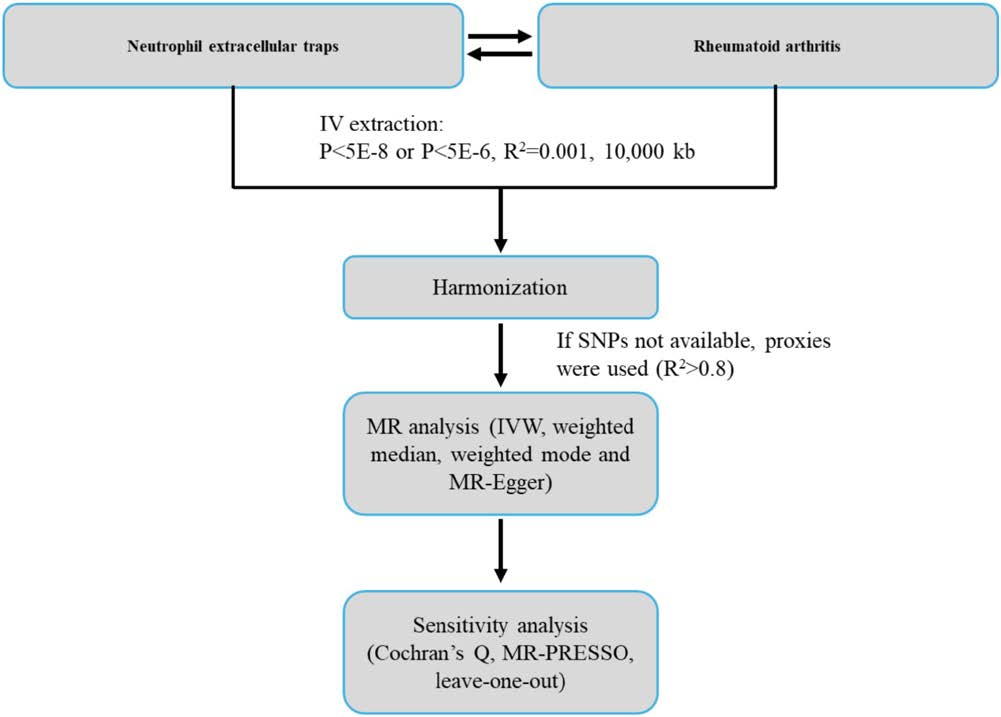
Schematic representation of the Mendelian randomization analysis. IV = instrumental variable, IVW = inverse variance weighted, MR = Mendelian randomization, MR-PRESSO = Mendelian Randomization Pleiotropy RESidual Sum and Outlier, SNPs = single-nucleotide polymorphisms.

This study used publicly available data from GWASs to investigate the causal association of both NETs and related cytokines on RA (Fig. 1). The key assumptions for MR studies are the relevance assumption, the independence assumption, and the no horizontal pleiotropy assumption. The relevance assumption involves the SNPs being used as instrumental variables (IVs) for the exposure and being associated with the exposure. The independence assumption stipulated that there are no common causes for the SNPs and the outcome of interest. Finally, the no horizontal pleiotropy assumption assumes that there is no independent pathway between the SNPs and the outcome other than through exposure.^[24]^

The datasets used here were obtained through studies already approved by ethics committees. Therefore, no additional ethical approval was required for the present study.

### 2.2. Data source

The genetic data pertaining to RA were sourced from a previously published study.^[26]^ The GWAS dataset, which comprises 14,361 RA patients and 43,923 healthy controls of European descent, encompasses 1,31,08,512 SNPs. The exposure data are as follows: NETs with 657 participants,^[27]^ neutrophil count determined in a large cohort of 5,19,288 Europeans,^[28]^ MPO levels assessed in 5357 Icelanders,^[29]^ MPO-DNA complexes evaluated in 5590 individuals from the Netherlands,^[30]^ and a series of ILs studied predominantly in Finnish populations: IL-6 in 8189 individuals, IL-18 in 3636, IL-1β in 3309, TNF-α in 3454, IL-4 in 8124, IL-5 in 3364, and IL-13 in 3557 individuals^[31]^ (Table S1, Supplemental Digital Content, https://links.lww.com/MD/Q425).

### 2.3. Instrumental variable selection

The selection criteria for the IVs included in this study were as follows. SNPs associated with NETs and NET-related indicators were screened, where *P* < 5 × 10^−8^ was the criterion for inclusion. For NETs, IL-6, IL-18, IL-1β, TNF-α, IL-4, IL-5, IL-13, and MPO-DNA, candidate IVs could not be identified, and a more lenient threshold of *P* < 5 × 10^−6^ had to be adopted.^[32]^ SNPs with a minimum minor allele frequency of >0.01 were selected.^[33]^ SNPs showing linkage disequilibrium were filtered out based on *R*^2^ < 0.001 and a window size of 10,000 kb.^[34]^ If a selected IV was not present in the outcome summary data, SNPs with high linkage disequilibrium (i.e., *R*^2^ > 0.8) were sought as proxy SNPs for replacement.^[35]^ Weak instrumental bias can affect the MR results. Hence, the *F*-value was calculated for each SNP to assess IV strength: *F* = *R*^2^ × (N − 2)/(1 − *R*^2^). The required *F*-value was >10.^[36]^

### 2.4. Mendelian randomization analysis

This bidirectional MR analysis first used NETs and NET-related factors as exposures and RA as the outcome. Then, RA was used as the exposure, and NETs and NET-related factors were used as outcomes. The inverse variance weighted (IVW) method was the primary method used to detect causal associations.^[37]^ If the IVW was statistically significant, the robustness of the association was evaluated with other MR analyses, including the MR-Egger,^[38]^ weighted median,^[39]^ and weighted mode^[40]^ methods. The results were shown as odds ratio (OR) and 95% confidence interval (CI). All analyses were conducted using the “TwoSampleMR” package in R 4.0.5 (The R Project for Statistical Computing, www.r-project.org).^[41]^ The analyses were visualized using scatter and forest plots.

### 2.5. Sensitivity analysis

Heterogeneity among IVs was detected using Cochran’s *Q* test, with *P* > .05 indicating low heterogeneity.^[42]^ Heterogeneity was also visually assessed using funnel plots. Horizontal pleiotropy was detected using the MR-Egger regression method, with intercepts approaching 0 or *P* > .05, indicating the absence of pleiotropy.^[42]^ The Mendelian Randomization Pleiotropy RESidual Sum and Outlier (MR-PRESSO) method was used to detect potential outliers (i.e., SNPs with *P* < .05).^[42]^ Such SNPs were removed to recalibrate the causal associations and correct for horizontal pleiotropy. Finally, a Leave-one-out analysis was used to assess the robustness and consistency of the results and to detect whether a single SNP was driving the associations all by itself.

## 3. Results

### 3.1. Instrument variable selection

In this study, for MR analysis using NETs, IL-6, IL-18, IL-1β, TNF-α, neutrophil count, IL-4, IL-5, IL-13, MPO, and MPO-DNA as exposures, respectively, 17, 8, 19, 5, 5, 430, 12, 6, 12, 5, and 10 IVs were selected. There were 0, 1, 3, 0, 0, 13, 1, 1, 1, 1, and 3 SNPs that did not match information in the summary data, and proxy SNPs were not found for any of them. The *R*^2^ values were 0.59, 0.023, 0.167, 0.025, 0.034, 0.093, 0.034, 0.045, 0.146, 0.083, and 0.04, respectively. All *F*-values were >10, indicating the absence of weak instrumental bias (Tables S2 and S3, Supplemental Digital Content, https://links.lww.com/MD/Q425).

### 3.2. Mendelian randomization analysis

**Figure 2.**
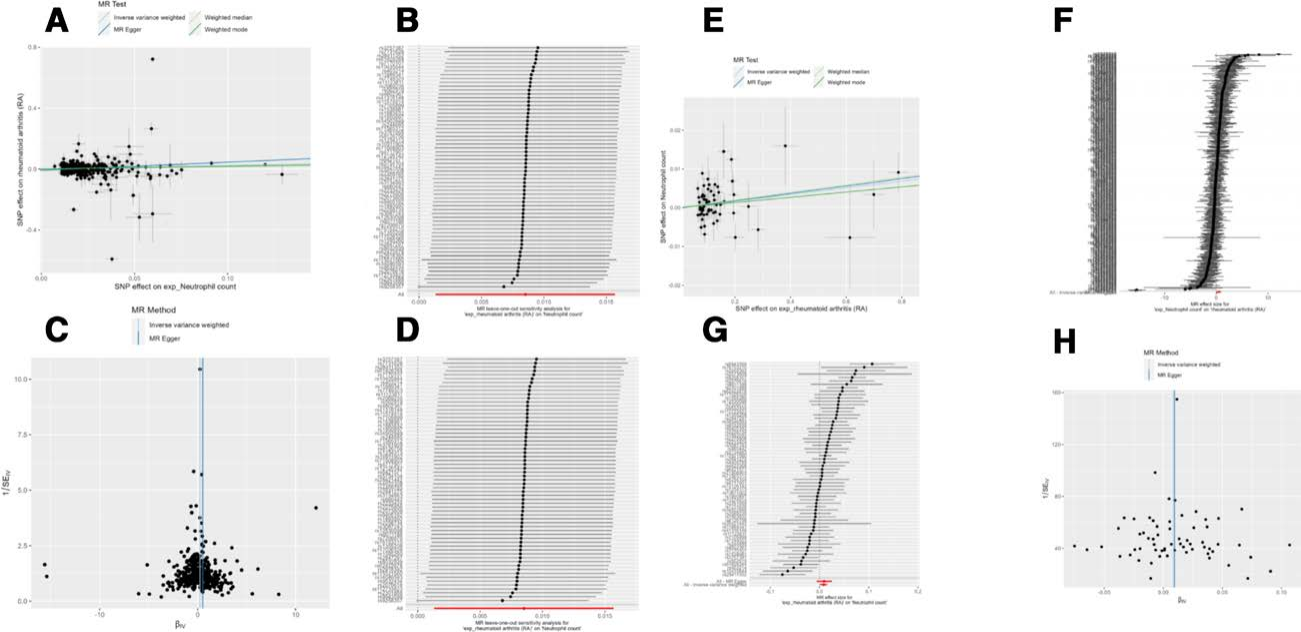
(A–D) Mendelian randomization analysis of rheumatoid arthritis (RA) as the outcome and neutrophil count as exposure. (A) Scatter plot. (B) Forest plot. (C) Funnel plot. (D) Leave-on-out analysis. (E–H) Mendelian randomization analysis of rheumatoid arthritis (RA) as the exposure and TNF-α levels as the outcome. (E) Scatter plot. (F) Forest plot. (G) Funnel plot. (H) Leave-on-out analysis. RA = rheumatoid arthritis, TNF = tumor necrosis factor.

When using RA as the outcome, the IVW analysis showed that neutrophil count had a causal association with RA (OR = 1.26, 95% CI: 1.01–1.58, *P* = .04), confirmed by the MR-Egger (*P* = .02), weighted median (*P* < .001), and weighted mode (*P* = .05) methods (Table 1 and Fig. 2A–D). All other exposures (i.e., IL-1β, IL-4, IL-5, IL-6, IL-18, IL-13, TNF-α, MPO, and blood MPO-DNA complexes) showed no statistically significant causal associations with RA (all IVW *P* > .05; Figs. S1 and S2, Supplemental Digital Content, https://links.lww.com/MD/Q426).

**Table 1:** Genetic prediction of causal associations between neutrophil extracellular traps (NETs)-related indicators and the risk of developing rheumatoid arthritis.

Exposure	Outcome	SNPs, n	Methods	OR (95% CI)	*P*
Neutrophil extracellular traps measurement	Rheumatoid arthritis	11	IVW	0.99 (0.97–1.01)	.30
			MR-Egger	0.97 (0.90–1.05)	.47
			Weighted median	0.99 (0.96–1.01)	.36
			Weighted mode	0.98 (0.95–1.02)	.46
Interleukin-6 levels		5	IVW	0.91 (0.79–1.05)	.19
			MR-Egger	1.04 (0.80–1.35)	.79
			Weighted median	0.88 (0.74–1.06)	.18
			Weighted mode	0.86 (0.69–1.07)	.25
Interleukin-18 levels		15	IVW	1.01 (0.96–1.07)	.58
			MR-Egger	1.03 (0.92–1.16)	.62
			Weighted median	1.01 (0.94–1.09)	.75
			Weighted mode	1.01 (0.92–1.11)	.78
Interleukin-1β levels		5	IVW	0.97 (0.88–1.07)	.60
			MR-Egger	0.91 (0.74–1.11)	.42
			Weighted median	0.97 (0.87–1.09)	.63
			Weighted mode	0.95 (0.85–1.08)	.49
TNF-α levels		5	IVW	1.04 (0.93–1.17)	.51
			MR-Egger	0.94 (0.78–1.13)	.55
			Weighted median	1.05 (0.92–1.19)	.49
			Weighted mode	1.05 (0.89–1.23)	.62
Neutrophil count		391	IVW	1.26 (1.01–1.58)	.04
			MR-Egger	1.69 (1.10–2.59)	.02
			Weighted median	1.20 (1.06–1.37)	<.001
			Weighted mode	1.19 (1.00–1.42)	.05
Neutrophil count (outlier corrected)		369	IVW	1.09 (1–1.18 )	.06
			MR-Egger	1.15 (0.97–1.36 )	.11
			Weighted median	1.26 (1.09–1.47 )	<.001
			Weighted mode	1.26 (1.1–1.46 )	<.001
Interleukin-4 levels		9	IVW	1.00 (0.87–1.16)	.95
			MR-Egger	0.86 (0.63–1.16)	.35
			Weighted median	1.06 (0.90–1.25)	.47
			Weighted mode	1.12 (0.89–1.39)	.36
Interleukin-5 levels		4	IVW	0.89 (0.77–1.03)	.12
			MR-Egger	0.94 (0.46–1.92)	.88
			Weighted median	0.89 (0.77–1.02)	.10
			Weighted mode	0.89 (0.77–1.03)	.22
Interleukin-13 levels		9	IVW	0.97 (0.91–1.02)	.21
			MR-Egger	0.99 (0.90–1.09)	.87
			Weighted median	0.98 (0.92–1.04)	.45
			Weighted mode	0.98 (0.92–1.04)	.47
Blood protein levels of myeloperoxidase		4	IVW	0.95 (0.81–1.1)	.48
			MR-Egger	1.11 (0.97–1.27)	.28
			Weighted median	1.01 (0.91–1.11)	.89
			Weighted mode	1.01 (0.91–1.12)	.90
Blood protein levels of myeloperoxidase-DNA complexes		7	IVW	0.89 (0.76–1.03)	.13
			MR-Egger	0.89 (0.54–1.44)	.65
			Weighted median	0.94 (0.77–1.13)	.5
			Weighted mode	0.97 (0.74–1.27)	.81

CI = confidence interval, IVW = inverse variance weighted, MR = Mendelian randomization, NETs = neutrophil extracellular traps, OR = odds ratio, SNPs = single-nucleotide polymorphisms, TNF = tumor necrosis factor.

### 3.3. Sensitivity analysis

Significant heterogeneity was observed for neutrophil count (*P* < .001) and MPO levels (*P* = .026; Table S4, Supplemental Digital Content, https://links.lww.com/MD/Q425). No horizontal pleiotropy was observed (Table S4, Supplemental Digital Content, https://links.lww.com/MD/Q425). The MR-PRESSO analysis detected 22 outliers for neutrophil count as the exposure (Table S5, Supplemental Digital Content, https://links.lww.com/MD/Q425). When correcting for those outliers, the causal association between neutrophil count and RA was lost (OR = 1.09, 95% CI: 1.00–1.18, *P* = .06). However, employing alternative analytical frameworks such as the weighted median analysis (OR = 1.26, 95% CI: 1.09–1.47, *P* < .001) and the weighted mode analysis (OR = 1.26, 95% CI: 1.10–1.46, *P* < .001), the data persistently implicated elevated neutrophil count as a probable risk factor for RA. Additionally, the leave-one-out sensitivity analysis ascertained the stability of our conclusions, affirming that no individual SNP singularly dominated or skewed the overall outcomes (Figs. S3 and S4, Supplemental Digital Content, https://links.lww.com/MD/Q426).

### 3.4. Results of reverse Mendelian randomization analyses

#### 3.4.1. Instrument variable selection using neutrophil parameters as the outcomes

In this study, for MR analysis using NETs, IL-6, IL-18, IL-1β, TNF-α, neutrophil count, IL-4, IL-5, IL-13, MPO, and MPO-DNA as outcomes, respectively, 73, 86, 86, 86, 86, 86, 86, 86, 86, 76, and 76 IVs were selected. There were 17, 4, 4, 4, 4, 4, 4, 4, 4, 14, and 14 SNPs that did not match information in the summary data, and proxy SNPs were not found for any of them. The *R*^2^ value was 0.16. All *F*-values were >10, indicating the absence of weak instrumental bias (Tables S6 and S7, Supplemental Digital Content, https://links.lww.com/MD/Q425).

#### 3.4.2. Mendelian randomization analysis using neutrophil parameters as the outcomes

**Figure 3.**
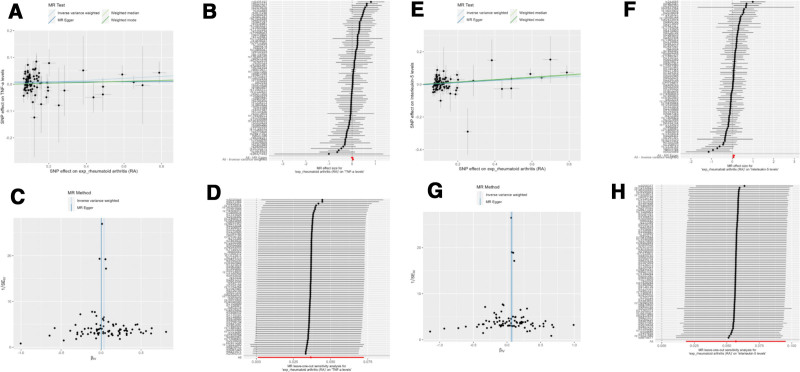
(A–D) Mendelian randomization analysis of rheumatoid arthritis (RA) as the exposure and neutrophil count as outcome. (A) Scatter plot. (B) Forest plot. (C) Funnel plot. (D) Leave-on-out analysis. (E–H) Mendelian randomization analysis of rheumatoid arthritis (RA) as the exposure and IL-5 levels as the outcome. (E) Scatter plot. (F) Forest plot. (G) Funnel plot. (H) Leave-on-out analysis. IL = interleukin, RA = rheumatoid arthritis.

**Figure 4.**
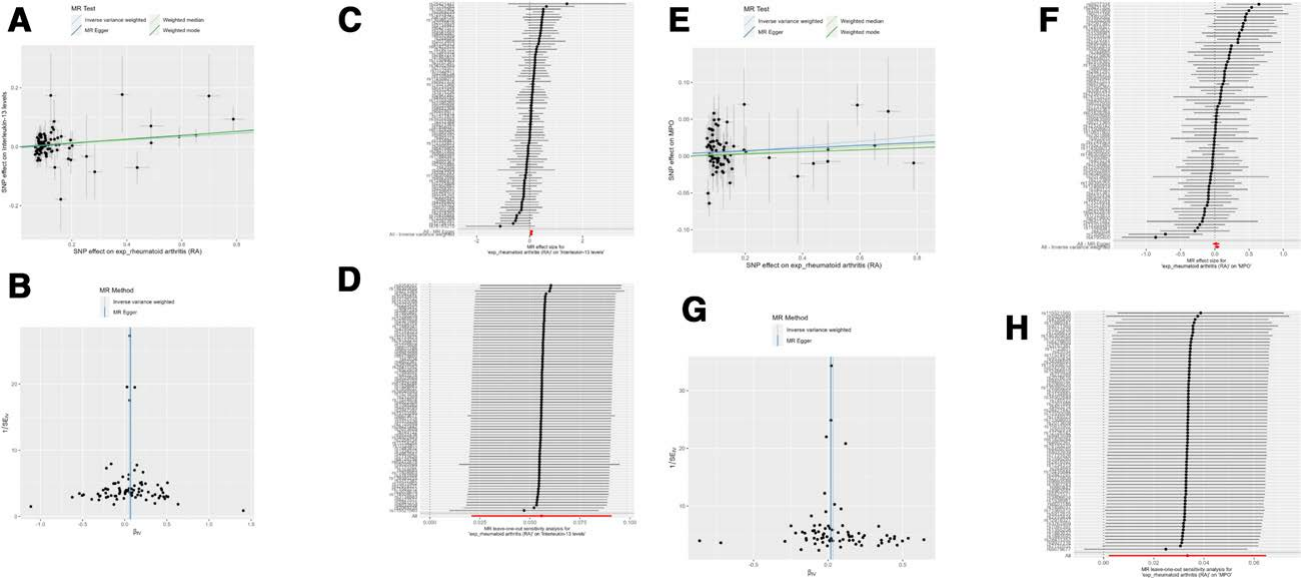
(A–D) Mendelian randomization analysis of rheumatoid arthritis (RA) as the exposure and IL-13 levels as the outcome. (A) Scatter plot. (B) Forest plot. (C) Funnel plot. (D) Leave-on-out analysis. (E–H) Mendelian randomization analysis of rheumatoid arthritis (RA) as the exposure and MPO levels as the outcome. (E) Scatter plot. (F) Forest plot. (G) Funnel plot. (H) Leave-on-out analysis. IL = interleukin, MPO = myeloperoxidase, RA = rheumatoid arthritis.

When using NETs and related factors as the outcomes, the IVW analysis showed that RA had a causal association with neutrophil count (OR = 1.01, 95% CI: 1.00–1.02, *P* = .02; Fig. 3A–D), TNF-α levels (OR = 1.04, 95% CI: 1.00–1.08, *P* = .04; Fig. 2E–H), IL-5 (OR = 1.06, 965% CI: 1.02–1.10, *P* = .004; Fig. 3E–H), IL-13 (OR = 1.06, 95% CI: 1.02–1.10, *P* = .002; Fig. 4A–D), and MPO (OR = 1.03, 95% CI: 1.00–1.07, *P* = .04; Fig. 4E–H). Notably, the associations with IL-5 and IL-13 were consistently validated by alternative methodologies, including weighted median, and weighted mode (all *P* < .05). The neutrophil count correlation was further corroborated by the weighted median approach alone (*P* = .02). Conversely, neither TNF-α nor MPO received supplementary validation from these alternative methods (Table 2 and Fig. 4). Regarding MPO-DNA complexes, while the weighted median and weighted mode methodologies hinted at a potential causal effect of RA (both with *P* = .04), this association was not substantiated by the IVW methodology, thereby necessitating a cautious interpretation of these findings. There were no statistically significant causal associations using RA as the exposure and NETs, IL-6, IL-18, IL-1β, IL-4, and MPO-DNA complexes (Figs. S5 and S6, Supplemental Digital Content, https://links.lww.com/MD/Q426).

**Table 2:** Genetic prediction of causal associations between neutrophil extracellular traps (NETs)-related indicators and the risk of developing rheumatoid arthritis.

Exposure	Outcome	SNPs	Methods	OR (95% CI)	*P*
Rheumatoid arthritis	Neutrophil extracellular traps measurement	71	IVW	1.10 (0.86–1.4)	.46
			MR-Egger	0.78 (0.44–1.38)	.40
			Weighted median	1.07 (0.72–1.59)	.73
			Weighted mode	1.06 (0.67–1.69)	.79
	Interleukin-6 levels	84	IVW	1.02 (1.00–1.04)	.11
			MR-Egger	1.02 (0.98–1.06)	.29
			Weighted median	1.02 (0.99–1.05)	.25
			Weighted mode	1.03 (1.00–1.06)	.10
	Interleukin-18 levels	84	IVW	0.99 (0.96–1.03)	.58
			MR-Egger	0.98 (0.93–1.04)	.55
			Weighted median	1.01 (0.96–1.06)	.74
			Weighted mode	1.01 (0.96–1.05)	.83
	Interleukin-1β levels	84	IVW	1.02 (0.99–1.05)	.13
			MR-Egger	1.01 (0.97–1.05)	.64
			Weighted median	1.01 (0.97–1.06)	.59
			Weighted mode	1.01 (0.98–1.05)	.44
	TNF-α levels	84	IVW	1.04 (1.00–1.08)	.04
			MR-Egger	1.00 (0.95–1.06)	.94
			Weighted median	1.02 (0.96–1.08)	.55
			Weighted mode	1.02 (0.97–1.07)	.43
	Neutrophil count	63	IVW	1.01 (1.00–1.02)	.02
			MR-Egger	1.01 (0.99–1.03)	.23
			Weighted median	1.01 (1.00–1.02)	.02
			Weighted mode	1.01 (1.00–1.02)	.26
	Neutrophil count (outlier corrected)	60	IVW	1.01 (1.00–1.01)	.04
			MR-Egger	1.01 (0.99–1.02)	.3
			Weighted median	1.01 (1.00–1.02)	.02
			Weighted mode	1.01 (1.00–1.02)	.26
	Interleukin-4 levels	78	IVW	1.01 (0.98–1.04)	.43
			MR-Egger	1.00 (0.96–1.04)	.99
			Weighted median	1.02 (0.98–1.06)	.37
			Weighted mode	1.01 (0.98–1.05)	.38
	Interleukin-5 levels	84	IVW	1.06 (1.02–1.10)	.004
			MR-Egger	1.07 (1.01–1.14)	.02
			Weighted median	1.07 (1.02–1.13)	.01
			Weighted mode	1.08 (1.03–1.13)	.003
	Interleukin-13 levels	84	IVW	1.06 (1.02–1.10)	.002
			MR-Egger	1.07 (1.02–1.13)	.01
			Weighted median	1.06 (1.00–1.12)	.04
			Weighted mode	1.07 (1.02–1.12)	.01
	Blood protein levels of myeloperoxidase	74	IVW	1.03 (1.00–1.07)	.04
			MR-Egger	1.02 (0.97–1.07)	.44
			Weighted median	1.02 (0.97–1.07)	.39
			Weighted mode	1.01 (0.98–1.05)	.47
	Blood protein levels of myeloperoxidase-DNA complexes	74	IVW	1.01 (0.97–1.05)	.51
			MR-Egger	1.08 (0.98–1.19)	.11
			Weighted median	1.06 (1.00–1.13)	.04
			Weighted mode	1.08 (1.00–1.17)	.04

CI = confidence interval, IVW = inverse variance weighted, MR = Mendelian randomization, NETs = neutrophil extracellular traps, OR = odds ratio, SNPs = single-nucleotide polymorphisms, TNF = tumor necrosis factor.

### 3.5. Sensitivity analysis (using neutrophil parameters as the outcomes)

Despite observing significant heterogeneity for IL-4 levels (*P* = .045; Table S8, Supplemental Digital Content, https://links.lww.com/MD/Q425 and Fig. S7, Supplemental Digital Content, https://links.lww.com/MD/Q426), our analysis did not uncover any evidence of horizontal pleiotropy, ensuring the robustness of our findings (Table S8, Supplemental Digital Content, https://links.lww.com/MD/Q425). The MR-PRESSO analysis detected 3 outliers for neutrophil count as the outcome. When correcting for those outliers, the causal association between RA and neutrophil count remained (IVW: OR = 1.01, 95% CI: 1.00–1.01, *P* = .04, weighted median: OR = 1.01, 95% CI: 1.00–1.02, *P* = .02; Table S9, Supplemental Digital Content, https://links.lww.com/MD/Q425). The leave-one-out analysis showed that no single SNP was driving the results (Fig. S8, Supplemental Digital Content, https://links.lww.com/MD/Q426).

## 4. Discussion

This 2-sample bidirectional MR analysis explored the causal association between genetically predicted NETs and RA. The results suggested that NETs had no causal associations with RA but that RA had causal associations with TNF-α levels, neutrophil count, IL-5, IL-13, and MPO, which are biomarkers associated with NETs.^[4,21]^ Further in-depth research is necessary to examine causal relationships between NETs and RA. The study findings refute the hypothesis that an increase in NETs is a factor in the onset of RA, instead suggesting that it is the presence of autoimmune diseases that leads to an increase in NETs.

Although they play a crucial role in the innate immune system, excessive NETs have been associated with many autoimmune diseases, including RA.^[5,7]^ NET formation appears to involve oxidative stress, commonly observed in patients with RA.^[8]^ Although it was long believed that autoimmune diseases like RA were solely due to defective adaptive immune responses, it is now known that the innate immune system is also involved and that neutrophils play important roles in autoimmunity,^[43]^ as supported by the present MR analysis. NETs carry proteins with immune functions that can activate other immune pathways.^[44]^ During NETosis, some of the neutrophil content is spilled along with the DNA, including autoantigens,^[45]^ which can trigger autoimmunity against proteins found in the nucleus.^[12]^ In addition, some cytokines in the NETs will activate the HLRP3 inflammasome of macrophages, leading to IL-1 and IL-18 release and neutrophil activation. The NET cytokines will also stimulate dendritic cells to produce type I interferon and trigger IL-6 and TNF-α, 2 proinflammatory cytokines involved in systemic inflammation. Of note, many studies about NETs and autoimmune diseases were not designed to examine causality and only reported associations between NETs and autoimmune diseases.^[5–7]^ A large body of evidence supports the role of NETs in the pathogenesis of RA, although evidence of direct causality of NETs in RA is conflicting.^[5–13]^ In the present bidirectional MR study, no causal associations were observed between NETs and RA. On the other hand, RA had a causal relationship with cytokines associated with NETs, including TNF-α levels, neutrophil count, IL-5, IL-13, and MPO. Furthermore, TNF-α, IL-5, and IL-13 are involved in the development and exacerbation of RA.^[46–48]^ Indeed, RA involves the formation of NETs, and NETs and related cytokines can exacerbate RA.^[6,15,20,21]^ A study reported a vicious circle in which RA and NETs exacerbate each other.^[14]^ Therefore, the present study supports the concept that autoimmune diseases increase NETs, which can then participate in the maintenance and exacerbation of the autoimmune condition.^[44]^

Nevertheless, it must be emphasized that the MR methodology analyses the genetic prediction of the exposure in association with the genetic prediction of the outcome. RA is a complex disease that involves many genes and environmental factors,^[1]^ and environmental factors can have an important impact on the outcome.^[49]^ The absence of genetic association can be because the genetic variation is insufficient to model the effect of exposure on outcome adequately, which is a known limitation of MR studies. Therefore, the results indicate that if there is a causal relationship between NETs and RA, it is not genetically predicted and can be influenced by several environmental and metabolic factors or that the causal relationship is indirect. For example, oxidative stress could be an indirect pathway linking NETs and RA. These results imply that in vivo and in vitro studies should be performed to evaluate the causality relationships between RA and NETs and related cytokines.

The present study observed bidirectional causal associations between neutrophil counts and RA. Although the causal association was lost after removing the outliers in the forward analysis, the association was observed in the reverse analysis. Nevertheless, the neutrophil counts appear involved in RA, warranting further study. In addition, the literature supports the role of neutrophils in RA. RA involves the presence of autoantibodies, which are known inducers of neutrophils, along with cytokines and chemokines.^[50]^ Hence, neutrophils play a central role in RA.^[51]^ Neutrophil depletion or inhibition decreases joint damage in RA models.^[52]^ Neutrophils in RA are different from those from healthy individuals and are in a proinflammatory state predisposing to oxidative stress. Activated neutrophils are found in the synovial fluid of patients with RA.^[51]^ Hypoxia, antiapoptotic cytokines (such as TNF-α, macrophage colony-stimulating factor, and IL-8), and autoantibodies found in synovial fluid in RA increase neutrophil survival for up to several days and increase their proliferation. Hence, the role of neutrophils in RA warrants further investigation.

TNF-α is well known to be involved in RA and is targeted by recent disease-modifying antirheumatic drugs,^[47]^ supporting the causal association of RA on TNF-α observed here. IL-5 also plays a role in RA since IL-5 targeting biologic disease-modifying antirheumatic drugs used to treat asthma can trigger RA as a side effect.^[46]^ IL-13 is another well-known actor in RA that modulates the activity of the immune cells.^[48]^ Increased serum PMO is another well-known feature of RA. Hence, the causal association of RA on TNF-α, IL-5, IL-13, and MPO levels follows what is known about the pathophysiology of RA, supporting the validity of the present MR analysis. Nevertheless, TNF-α, IL-5, IL-13, and MPO could be investigated as markers for the development of RA in future longitudinal studies. Besides their involvement in RA, future studies should also examine those cytokines in relation to NETs induced by autoimmune diseases.

The main strength of MR analyses is the use of data from thousands of individuals. On the other hand, the GWAS data were mainly from individuals of European ancestry, and their generalizability to other populations remains unknown. Some participants could have been included in both the exposure and the outcome, but it is impossible to determine how many. Still, using stringent statistics should minimize the impact of eventual overlap. Finally, using different criteria or thresholds during the IV selection process, different SNPs could be selected as IVs, ultimately affecting the causal associations.

NETs had no causal associations with RA, but RA had causal associations with TNF-α levels, neutrophil count, IL-5, IL-13, and MPO. Further in-depth research is necessary to examine causal relationships between NETs and RA.

## Author contributions

**Conceptualization:** Junwei Luo.

**Data curation:** Junwei Luo, Xinlong Li.

**Formal analysis:** Junwei Luo.

**Writing – original draft:** Junwei Luo, Xinlong Li, Xi Wang, Jiaqi Yuan.

**Writing – review & editing:** Junwei Luo, Xinlong Li, Xi Wang, Jiaqi Yuan.

## Supplementary Material




