# Supplementary material for: Causal association between neutrophil extracellular traps and rheumatoid arthritis: A 2-sample bidirectional Mendelian randomization study
Source: Medicine (Baltimore). 2025 Oct 31;104(44):e45331. doi: 10.1097/MD.0000000000045331 (PMC12582744; doi:10.1097/MD.0000000000045331)
Supplement: Supplementary file 1 [file medi-104-e45331-s001.docx]

**Table S1. GWAS datasets used in this study**

| **Trait** | **GWAS ID** | **Sample size (case/control)** | **Number of SNPs** |
| --- | --- | --- | --- |
| Rheumatoid arthritis (RA) | ebi-a-GCST90013534 | 14,361/43,923 | 13,108,512 |
| Neutrophil extracellular trap measurement | GCST90137414 | 657 |  |
| Interleukin-6 levels | GCST004446 | 8189 |  |
| Interleukin-18 levels | GCST004441 | 3636 |  |
| Interleukin-1β levels | GCST004448 | 3309 |  |
| TNF-α levels | GCST004426 | 3454 |  |
| Neutrophil count | GCST90002351 | 519288 |  |
| Interleukin-4 levels | GCST004453 | 8124 |  |
| Interleukin-5 levels | GCST004452 | 3364 |  |
| Interleukin-13 levels | GCST004443 | 3557 |  |
| Blood protein levels of myeloperoxidase | GCST90087967 | 5357 |  |
| Blood protein levels of myeloperoxidase-DNA complexes | GCST90013658 | 5590 |  |

**Table S2. IV selection for NETs as exposure and RA as outcomes**

| **Exposure** | **Outcome** | **IVs, n** | **Mean F** | **F range** | **Lacking SNPs** | **Proxies, n** | **R^2^** |
| --- | --- | --- | --- | --- | --- | --- | --- |
| Neutrophil extracellular traps measurement | Rheumatoid arthritis | 17 | 23.60 | 20.59-28.57 | 0 | - | 0.590 |
| Interleukin-6 levels |  | 8 | 23.77 | 21.64-29.30 | 1 | 0 | 0.023 |
| Interleukin-18 levels |  | 19 | 32.43 | 20.60-96.11 | 3 | 0 | 0.167 |
| Interleukin-1β levels |  | 5 | 16.29 | 14.61-20.02 | 0 | - | 0.025 |
| TNF-a levels |  | 5 | 23.50 | 22.06-24.93 | 0 | - | 0.034 |
| Neutrophil count |  | 430 | 111.94 | 29.85-3750.82 | 13 | 0 | 0.093 |
| Interleukin-4 levels |  | 12 | 23.37 | 21.08-26.62 | 1 | 0 | 0.034 |
| Interleukin-5 levels |  | 6 | 25.31 | 22.04-37.91 | 1 | 0 | 0.045 |
| Interleukin-13 levels |  | 12 | 45.27 | 21.08-292.67 | 1 | 0 | 0.146 |
| Blood protein levels of myeloperoxidase |  | 5 | 91.40 | 31.30-240.40 | 1 | 0 | 0.083 |
| Blood protein levels of myeloperoxidase-DNA complexes |  | 10 | 22.69 | 21.12-25.84 | 3 | 0 | 0.040 |

​​**Supplementary Table 3. Detailed information on IV for NETs as exposure and RA as outcomes**

Neutrophil count and RA

| SNP | chr.exposure | pval.exposure | effect_allele.exposure | other_allele.exposure | r2 | F | pval.outcome | effect_allele.outcome | other_allele.outcome |
| --- | --- | --- | --- | --- | --- | --- | --- | --- | --- |
| rs172933 | 1 | 2.24001e-08 | C | T | 6.03264525348128e-05 | 31.328572172615 | 0.9028 | C | T |
| rs301817 | 1 | 5.88979e-13 | A | C | 0.000100012289394236 | 51.9401763663253 | 0.0167201 | A | C |
| rs10864368 | 1 | 4.00959e-35 | C | T | 0.000294749616898047 | 153.104477046474 | 0.1132 | C | T |
| rs284317 | 1 | 1.59993e-11 | G | A | 8.75845697032871e-05 | 45.4854246842899 | 0.1538 | G | A |
| rs1135172 | 1 | 2.45997e-08 | G | A | 5.99847977709924e-05 | 31.1511342897983 | 0.6432 | G | A |
| rs10917107 | 1 | 1.13999e-08 | A | G | 6.28360991117841e-05 | 32.6319570282479 | 0.4854 | A | G |
| rs112203773 | 1 | 7.69999e-09 | A | C | 6.43054011843608e-05 | 33.395042040998 | 0.0646294 | A | C |
| rs3762297 | 1 | 2.83009e-21 | T | C | 0.000172852988407742 | 89.7756549285434 | 0.4407 | T | C |
| rs3917932 | 1 | 2.99916e-154 | G | C | 0.00134708397434296 | 700.465434462001 | 0.9679 | G | C |
| rs11800162 | 1 | 5.06991e-19 | T | C | 0.000153117491382394 | 79.5239461371349 | 0.6386 | T | C |
| rs3754224 | 1 | 2.70023e-14 | C | T | 0.000111745676528334 | 58.0344504806236 | 0.726599 | C | T |
| rs140958972 | 1 | 1.45011e-23 | G | A | 0.000193100300165112 | 100.293649205306 | 0.0468101 | G | A |
| rs12121236 | 1 | 8.77001e-14 | A | C | 0.000107306208970567 | 55.7285920554348 | 0.4072 | A | C |
| rs111322601 | 1 | 1.31e-08 | T | C | 6.23306127687085e-05 | 32.3694321887549 | 0.1174 | T | C |
| rs74076327 | 1 | 7.85055e-17 | C | T | 0.000133905889209576 | 69.5447660378203 | 0.6693 | C | T |
| rs6678033 | 1 | 1.45881e-102 | A | G | 0.000889744086101808 | 462.443103511972 | 0.3856 | A | G |
| rs17440390 | 1 | 1.34999e-08 | T | C | 6.22203958873158e-05 | 32.3121909760552 | 0.8101 | T | C |
| rs41313381 | 1 | 1.16011e-26 | A | C | 0.000220184649288796 | 114.363986984948 | 0.5445 | A | C |
| rs116631966 | 1 | 1.02e-10 | A | G | 8.0593943692281e-05 | 41.8546798779031 | 0.5489 | A | G |
| rs55851397 | 1 | 1.17999e-09 | A | C | 7.13306020183338e-05 | 37.0436253437892 | 0.4241 | A | C |
| rs694180 | 1 | 1.23994e-20 | G | A | 0.000167218518526098 | 86.8487583320476 | 0.8378 | G | A |
| rs6679677 | 1 | 8.6896e-33 | A | C | 0.00027410677990445 | 142.378840314907 | 1.40929e-145 | A | C |
| rs68002561 | 1 | 2.43002e-09 | G | A | 6.86601689345652e-05 | 35.656712681271 | 0.782899 | G | A |
| rs7555995 | 1 | 2.86022e-12 | G | C | 9.40731254853655e-05 | 48.8554530259566 | 0.2685 | G | C |
| rs114427170 | 1 | 2.81002e-09 | A | G | 6.81047617852591e-05 | 35.368258075211 | 0.1101 | A | G |
| rs61801809 | 1 | 1.78999e-09 | T | C | 6.97816656190553e-05 | 36.2391708423588 | 0.8306 | T | C |
| rs3845532 | 1 | 1.27997e-21 | C | A | 0.000175880281373696 | 91.348234146546 | 0.2565 | C | A |
| rs2312675 | 1 | 2.60976e-17 | C | T | 0.000138130425326346 | 71.7391053991582 | 0.3551 | C | T |
| rs60124939 | 1 | 1.51985e-12 | T | C | 9.64509007736822e-05 | 50.0904337266154 | 0.6401 | T | C |
| rs41272536 | 1 | 2.33024e-13 | G | A | 0.000103580665828241 | 53.7935616082125 | 0.0527096 | G | A |
| rs16843353 | 1 | 8.83995e-10 | C | G | 7.24363599642127e-05 | 37.6179125250289 | 0.00808705 | C | G |
| rs2808519 | 1 | 1.50003e-13 | T | G | 0.00010523706066312 | 54.653883897604 | 0.759799 | T | G |
| rs6593925 | 1 | 9.2406e-13 | C | G | 9.83373001341632e-05 | 51.0702053435799 | 0.516 | C | G |
| rs4844622 | 1 | 1.44012e-25 | T | C | 0.000210478896060631 | 109.321753941826 | 0.0917994 | T | C |
| rs35090781 | 1 | 4.42996e-08 | T | C | 5.7757122292752e-05 | 29.9941973854405 | 0.9702 | T | C |
| rs7536608 | 1 | 3.72992e-25 | A | C | 0.000206966797179402 | 107.497208593071 | 0.6202 | A | C |
| rs11580229 | 1 | 2.75994e-30 | A | G | 0.000252128379043773 | 130.959756111153 | 0.144 | A | G |
| rs486650 | 1 | 5.19039e-13 | T | C | 0.000100489121595369 | 52.1878383068022 | 0.7641 | T | C |
| rs2804804 | 1 | 3.2802e-23 | G | A | 0.000189893785670471 | 98.6279132134893 | 0.8591 | G | A |
| rs1886654 | 1 | 3.07964e-80 | C | T | 0.000692803536034785 | 360.012595012201 | 0.0481604 | C | T |
| rs35020138 | 1 | 1.42004e-17 | C | T | 0.00014044127747995 | 72.9394329245919 | 0.8924 | C | T |
| rs56188865 | 1 | 1.93019e-43 | C | T | 0.000367879166071421 | 191.104804108531 | 0.3952 | C | T |
| rs12037562 | 1 | 2.56e-10 | C | T | 7.70858608656301e-05 | 40.0326943001725 | 0.4919 | C | T |
| rs6432335 | 2 | 2.42003e-10 | G | T | 7.73165599724281e-05 | 40.1525116159147 | 0.0145399 | G | T |
| rs4632345 | 2 | 3.23996e-09 | A | G | 6.75268369996258e-05 | 35.0681091166759 | 0.1032 | A | G |
| rs36101491 | 2 | 1.40994e-23 | T | C | 0.000193101952581784 | 100.294507613637 | 0.155 | T | C |
| rs7575465 | 2 | 5.70953e-11 | A | G | 8.27787355775004e-05 | 42.9893970910323 | 0.3634 | A | G |
| rs1260326 | 2 | 6.91035e-62 | C | T | 0.000530668346328807 | 275.714956091508 | 0.5006 | C | T |
| rs116447416 | 2 | 1.46994e-12 | G | A | 9.6579397329925e-05 | 50.1571730714144 | 0.9566 | G | A |
| rs17030394 | 2 | 6.29071e-16 | G | A | 0.0001260168528371 | 65.4470348717284 | 0.0497004 | G | A |
| rs77552263 | 2 | 7.18952e-42 | A | G | 0.000354096699217489 | 183.942592014531 | 0.2392 | A | G |
| rs75475627 | 2 | 3.76011e-19 | G | C | 0.000154263375464383 | 80.1191706451008 | 0.3428 | G | C |
| rs2421200 | 2 | 1.02991e-26 | T | G | 0.000220707939475962 | 114.635844099656 | 0.05425 | T | G |
| rs6731993 | 2 | 2.45019e-31 | T | A | 0.000261302385044132 | 135.726135883046 | 1.40101e-08 | T | A |
| rs4599108 | 2 | 6.57961e-22 | T | C | 0.000178511553377422 | 92.7151012238887 | 0.00187599 | T | C |
| rs2309996 | 2 | 4.26973e-30 | G | T | 0.000250371945439073 | 130.047206231297 | 0.29 | G | T |
| rs35789178 | 2 | 3.41036e-13 | G | T | 0.000102123458859983 | 53.0366987486877 | 0.7848 | G | T |
| rs11691193 | 2 | 2.52e-10 | T | C | 7.71405742138055e-05 | 40.0611105582623 | 0.5407 | T | C |
| rs75726191 | 2 | 1.29003e-15 | T | C | 0.00012326216957195 | 64.0162097652431 | 0.2038 | T | C |
| rs6734238 | 2 | 1.58016e-90 | G | A | 0.000783956686101616 | 407.417129081339 | 7.67803e-06 | G | A |
| rs11683933 | 2 | 5.72005e-13 | A | C | 0.000100186073695442 | 52.0304381903263 | 0.5977 | A | C |
| rs4954387 | 2 | 3.67003e-09 | G | A | 6.7087375674111e-05 | 34.8398722799065 | 0.2167 | G | A |
| rs354703 | 2 | 4.08037e-14 | C | T | 0.000110164994188673 | 57.2134420907746 | 0.5526 | C | T |
| rs3856364 | 2 | 5.27959e-12 | G | C | 9.17506058645041e-05 | 47.6491769578097 | 0.00609705 | G | C |
| rs188653407 | 2 | 2.57988e-15 | G | C | 0.000120630540115897 | 62.6493080744944 | 0.4556 | G | C |
| rs2729707 | 2 | 2.67979e-44 | G | A | 0.00037546362840365 | 195.046238507635 | 0.3419 | G | A |
| rs11689257 | 2 | 2.50998e-10 | A | G | 7.71653272480047e-05 | 40.0739664460423 | 0.0844306 | A | G |
| rs2632372 | 2 | 6.68036e-28 | C | T | 0.000231125324508256 | 120.047891370444 | 0.1614 | C | T |
| rs62189859 | 2 | 2.95999e-10 | C | T | 7.65605729642385e-05 | 39.7598777313277 | 0.8387 | C | T |
| rs6740847 | 2 | 3.95003e-29 | G | A | 0.000241947875315005 | 125.670550103415 | 0.4903 | G | A |
| rs13392977 | 2 | 3.72992e-11 | A | G | 8.43627015739312e-05 | 43.8120659537658 | 0.3568 | A | G |
| rs2052249 | 2 | 1.23001e-08 | A | C | 6.25516616377928e-05 | 32.4842341080634 | 0.693999 | A | C |
| rs1047891 | 2 | 8.79023e-21 | A | C | 0.000168472394163098 | 87.500097026214 | 0.001327 | A | C |
| rs11695689 | 2 | 2.87998e-09 | C | A | 6.80153322023249e-05 | 35.3218122228088 | 0.542 | C | A |
| rs55729107 | 2 | 7.87046e-14 | G | A | 0.000107662969984794 | 55.9138928872956 | 0.589899 | G | A |
| rs9247 | 2 | 1.31e-10 | T | C | 7.9612879143716e-05 | 41.3451451650689 | 0.00490095 | T | C |
| rs1344760 | 2 | 5.62989e-37 | T | C | 0.000311178440170584 | 161.64090664762 | 0.5434 | T | C |
| rs56217149 | 3 | 4.45e-10 | A | G | 7.50256472512028e-05 | 38.9626914596335 | 0.0717993 | A | G |
| rs1366045 | 3 | 1.20005e-28 | C | T | 0.000237755922948157 | 123.492683320957 | 0.4409 | C | T |
| rs6442061 | 3 | 3.67959e-17 | T | G | 0.000136857866651491 | 71.0781017393556 | 0.0242198 | T | G |
| rs6779340 | 3 | 8.6896e-14 | G | C | 0.000107350090339993 | 55.7513839283951 | 0.2102 | G | C |
| rs3733120 | 3 | 1.63001e-09 | C | G | 7.01258770258183e-05 | 36.4179400172123 | 0.4566 | C | G |
| rs12487658 | 3 | 2.06016e-12 | T | C | 9.53458109418685e-05 | 49.5164659683618 | 0.175 | T | C |
| rs11721064 | 3 | 3.22033e-13 | T | G | 0.000102323306052806 | 53.1404978183593 | 0.9939 | T | G |
| rs7639292 | 3 | 4.82948e-18 | T | C | 0.000144552334213967 | 75.0748556702618 | 0.9733 | T | C |
| rs4687892 | 3 | 2.5398e-12 | C | T | 9.45275912779229e-05 | 49.0914952651471 | 0.1354 | C | T |
| rs2734031 | 3 | 4.20049e-91 | C | T | 0.000788742216346024 | 409.906100803903 | 0.00317797 | C | T |
| rs6764912 | 3 | 3.13979e-20 | A | G | 0.000163722683525582 | 85.0328192386189 | 0.484 | A | G |
| rs55659176 | 3 | 5.81969e-17 | T | C | 0.000135078427263167 | 70.1538124464329 | 0.2018 | T | C |
| rs11712552 | 3 | 6.4506e-26 | G | A | 0.000213615364901266 | 110.951169252625 | 0.2541 | G | A |
| rs2046934 | 3 | 1.66001e-09 | A | G | 7.00847962532713e-05 | 36.3966043557768 | 0.664899 | A | G |
| rs56082403 | 3 | 5.08042e-15 | C | T | 0.000118116848618202 | 61.3436715727202 | 0.5797 | C | T |
| rs11716015 | 3 | 1.16001e-10 | T | C | 8.01405071137051e-05 | 41.6191787591389 | 0.5144 | T | C |
| rs3749440 | 3 | 2.19989e-18 | G | A | 0.0001474496819253 | 76.5798472023732 | 0.1132 | G | A |
| rs56174170 | 3 | 2.04e-09 | C | T | 6.92879414770298e-05 | 35.9827511485967 | 0.1779 | C | T |
| rs34834004 | 3 | 6.77018e-11 | G | T | 8.20888012678785e-05 | 42.6310647881968 | 0.4034 | G | T |
| rs59519784 | 3 | 1.84999e-09 | A | G | 6.9664401157352e-05 | 36.1782685568106 | 0.4439 | A | G |
| rs789858 | 3 | 1.07004e-12 | T | C | 9.77767365094377e-05 | 50.7790554053604 | 0.3515 | T | C |
| rs7626444 | 3 | 1.26999e-26 | C | G | 0.000219977835118815 | 114.256543994705 | 0.2291 | C | G |
| rs35734242 | 4 | 5.42001e-31 | C | T | 0.000258191941786347 | 134.110086826196 | 0.649 | C | T |
| rs6855981 | 4 | 1.85012e-16 | A | G | 0.000130656332872945 | 67.8568704021072 | 0.3505 | A | G |
| rs6831368 | 4 | 3.65006e-27 | G | A | 0.00022468545077082 | 116.702230282193 | 0.8309 | G | A |
| rs140311179 | 4 | 1.36e-08 | G | C | 6.21740571974655e-05 | 32.2881249495705 | 0.4685 | G | C |
| rs28530750 | 4 | 2.35993e-31 | A | G | 0.000261482669737472 | 135.819804162289 | 0.778901 | A | G |
| rs7658676 | 4 | 3.57998e-08 | T | C | 5.8549106249939e-05 | 30.4055114036236 | 0.02003 | T | C |
| rs116114800 | 4 | 2.83002e-08 | A | G | 5.94266137202567e-05 | 30.8612425114749 | 0.8264 | A | G |
| rs218264 | 4 | 5.58985e-46 | T | A | 0.000390439308389581 | 202.828859055843 | 0.9223 | T | A |
| rs723585 | 4 | 2.9703e-31 | G | A | 0.000260668321591061 | 135.396703677232 | 0.0009438 | G | A |
| rs7684253 | 4 | 3.29003e-13 | T | C | 0.00010225498534778 | 53.1050125735897 | 0.540201 | T | C |
| rs11723621 | 4 | 2.66993e-37 | G | A | 0.000313934759100409 | 163.073119634743 | 0.9105 | G | A |
| rs16850073 | 4 | 2.11836e-149 | T | C | 0.00130409014117978 | 678.080030535435 | 0.1967 | T | C |
| rs1371794 | 4 | 1e-200 | C | A | 0.00257736488549406 | 1341.84793367459 | 0.0164399 | C | A |
| rs7687658 | 4 | 1.13999e-08 | G | A | 6.28359257937101e-05 | 32.6318670152868 | 0.0335398 | G | A |
| rs17213043 | 4 | 1.97015e-15 | G | C | 0.0001216818896974 | 63.195391508063 | 0.3609 | G | C |
| rs7679673 | 4 | 2.14981e-33 | A | C | 0.000279567256069503 | 145.215959762716 | 0.0766302 | A | C |
| rs2522440 | 4 | 2.90001e-08 | C | T | 5.93449424681645e-05 | 30.8188267360228 | 0.776401 | C | T |
| rs4145952 | 4 | 1.05999e-11 | A | C | 8.91012426948815e-05 | 46.2731509093036 | 0.3362 | A | C |
| rs2290846 | 4 | 9.91973e-19 | A | G | 0.000150484530941079 | 78.1562714441178 | 0.4898 | A | G |
| rs6817881 | 4 | 1.3499e-13 | T | C | 0.000105626973786184 | 54.8564030253766 | 0.0489396 | T | C |
| rs4535497 | 5 | 1.74985e-12 | A | C | 9.59448781130211e-05 | 49.8276126800235 | 0.8157 | A | C |
| rs7705526 | 5 | 9.18967e-54 | A | C | 0.00045917984613244 | 238.555205321204 | 0.6339 | A | C |
| rs13189073 | 5 | 2.66999e-08 | A | G | 5.96438410293338e-05 | 30.9740590446123 | 0.4867 | A | G |
| rs464609 | 5 | 3.02002e-08 | A | G | 5.91819182235702e-05 | 30.7341604932179 | 0.0118801 | A | G |
| rs62360185 | 5 | 8.05008e-11 | G | A | 8.14466820198416e-05 | 42.297566715822 | 0.8339 | G | A |
| rs12658947 | 5 | 3.01002e-09 | G | A | 6.78550266524921e-05 | 35.2385564834554 | 0.5178 | G | A |
| rs66514959 | 5 | 2.48028e-38 | A | G | 0.000323034085987975 | 167.801283910727 | 0.0624195 | A | G |
| rs6859727 | 5 | 4.29042e-43 | C | T | 0.000365009225309929 | 189.613391211326 | 0.3446 | C | T |
| rs62375385 | 5 | 5.45997e-09 | C | T | 6.55974386854474e-05 | 34.0660661918995 | 0.1132 | C | T |
| rs2432142 | 5 | 1.89017e-14 | A | G | 0.000113151064779887 | 58.7644130811998 | 0.8401 | A | G |
| rs1445171 | 5 | 1.15001e-13 | C | T | 0.000106214255255856 | 55.1614346854962 | 0.4078 | C | T |
| rs1966479 | 5 | 1.21004e-12 | G | A | 9.72872390508693e-05 | 50.524816637684 | 0.2105 | G | A |
| rs6878780 | 5 | 6.01035e-12 | C | T | 9.13159725709684e-05 | 47.4234366497282 | 0.8997 | C | T |
| rs2522051 | 5 | 1.88018e-35 | C | T | 0.000297694130848451 | 154.634428193469 | 0.755499 | C | T |
| rs10479004 | 5 | 7.06968e-13 | T | G | 9.93549275183947e-05 | 51.5987494813319 | 0.601501 | T | G |
| rs6860961 | 5 | 2.30001e-10 | C | T | 7.75292550712743e-05 | 40.2629783076569 | 0.8067 | C | T |
| rs6881942 | 5 | 2.75994e-28 | T | C | 0.000234581840425209 | 121.843647894212 | 0.785199 | T | C |
| rs114363252 | 5 | 9.52994e-10 | A | G | 7.21503884950196e-05 | 37.4693900710751 | 0.562 | A | G |
| rs11960649 | 5 | 1.9602e-53 | C | A | 0.000456136508230959 | 236.973395030175 | 0.6411 | C | A |
| rs284440 | 5 | 6.16027e-15 | G | T | 0.000117328302378469 | 60.9340941227261 | 0.590999 | G | T |
| rs9885207 | 5 | 1.22011e-16 | C | T | 0.000132270352818023 | 68.6952287756071 | 0.5921 | C | T |
| rs2561758 | 5 | 3.4898e-55 | G | A | 0.000471526639262961 | 244.972693547206 | 0.1146 | G | A |
| rs13190036 | 5 | 3.62994e-12 | G | A | 9.31511772636767e-05 | 48.3766085745863 | 0.0909494 | G | A |
| rs10447304 | 5 | 5.57956e-18 | A | G | 0.000143921070859373 | 74.746954864068 | 0.0633899 | A | G |
| rs155795 | 5 | 1.71002e-11 | C | G | 8.72686258400717e-05 | 45.3213307682449 | 0.8154 | C | G |
| rs12203592 | 6 | 1.66001e-08 | T | C | 6.14404168058462e-05 | 31.9071086674963 | 0.2387 | T | C |
| rs6936191 | 6 | 9.86961e-20 | C | T | 0.00015939357906245 | 82.7840493429376 | 0.3179 | C | T |
| rs3777755 | 6 | 4.77969e-11 | T | C | 8.34550153845746e-05 | 43.3406381126147 | 0.04605 | T | C |
| rs6915310 | 6 | 1.71002e-36 | T | C | 0.000306694682826442 | 159.311114938078 | 0.23 | T | C |
| rs4712614 | 6 | 1.03992e-23 | G | T | 0.000194290906801584 | 100.912154143303 | 0.735301 | G | T |
| rs1555244 | 6 | 5.7597e-27 | T | C | 0.000222965997007256 | 115.808942178165 | 2.33292e-13 | T | C |
| rs1611236 | 6 | 3.34965e-20 | A | G | 0.000163441461727457 | 84.8867369069654 | 4.5436e-19 | A | G |
| rs73428122 | 6 | 3.53997e-09 | G | T | 6.72251616806966e-05 | 34.9114322351987 | 3.73422e-17 | G | T |
| rs9265980 | 6 | 1.62181e-115 | A | G | 0.00100487127077726 | 522.340467646343 | 0.01704 | A | G |
| rs200801362 | 6 | 1.7402e-54 | C | T | 0.000465451659080041 | 241.815083468829 | 3.75898e-10 | C | T |
| rs2736429 | 6 | 2.03002e-46 | G | A | 0.000394480213734339 | 204.928892662628 | 4.2776e-17 | G | A |
| rs9378212 | 6 | 1e-200 | T | C | 0.00184589562700294 | 960.320407805139 | 1 | T | C |
| rs2856821 | 6 | 1.35988e-13 | C | T | 0.00010558250594886 | 54.8333066220824 | 1.737e-66 | C | T |
| rs7738554 | 6 | 5.70033e-11 | A | T | 8.27500252167387e-05 | 42.9744857344776 | 0.0442996 | A | T |
| rs10948036 | 6 | 3.4898e-36 | A | C | 0.000304111229954253 | 157.968744227126 | 0.8895 | A | C |
| rs68016381 | 6 | 2.22998e-14 | T | C | 0.000112485034671553 | 58.4184749186287 | 0.8533 | T | C |
| rs68137036 | 6 | 1.02e-09 | G | A | 7.1920486214586e-05 | 37.3499878337126 | 0.658999 | G | A |
| rs12189880 | 6 | 1.53993e-12 | T | C | 9.64294423810867e-05 | 50.0792885341732 | 0.0938296 | T | C |
| rs915125 | 6 | 1.91999e-16 | T | C | 0.000130570176467436 | 67.8121188973999 | 0.5068 | T | C |
| rs10944174 | 6 | 3.36001e-08 | G | A | 5.87786845801069e-05 | 30.5247422050594 | 0.03904 | G | A |
| rs6454596 | 6 | 1.38007e-39 | C | G | 0.000333965513799428 | 173.481552654717 | 0.4761 | C | G |
| rs207253 | 6 | 9.41998e-10 | A | G | 7.21777835193399e-05 | 37.4836179770878 | 0.1642 | A | G |
| rs9487053 | 6 | 2.49977e-41 | C | T | 0.000349473626873153 | 181.540205318428 | 0.0201298 | C | T |
| rs62429983 | 6 | 1.76999e-09 | A | G | 6.98650063084395e-05 | 36.2824545397996 | 0.6159 | A | G |
| rs1490384 | 6 | 2.18978e-14 | T | C | 0.000112515147643379 | 58.4341156822933 | 0.8222 | T | C |
| rs9402685 | 6 | 1.05003e-52 | C | T | 0.000449837621708886 | 233.699506056719 | 0.0288503 | C | T |
| rs12214269 | 6 | 6.59933e-24 | A | G | 0.000196035436320784 | 101.818417603195 | 0.000650894 | A | G |
| rs6924387 | 6 | 1.72982e-18 | G | A | 0.00014840649747652 | 77.0768551547022 | 0.5623 | G | A |
| rs72978754 | 6 | 5.38022e-11 | C | T | 8.30162376657252e-05 | 43.1127490507054 | 0.0310599 | C | T |
| rs9390461 | 6 | 3.4898e-20 | G | A | 0.00016325655430719 | 84.7906856951474 | 0.5117 | G | A |
| rs9479025 | 6 | 4.47003e-08 | T | A | 5.77598951806229e-05 | 29.9956374736413 | 0.5975 | T | A |
| rs10945542 | 6 | 4.82003e-10 | T | C | 7.47400617753472e-05 | 38.8143687073879 | 0.2808 | T | C |
| rs212409 | 6 | 4.70977e-28 | A | G | 0.000232343766650207 | 120.680904664672 | 1.44901e-07 | A | G |
| rs6978000 | 7 | 3.18999e-08 | T | A | 5.90169263648019e-05 | 30.6484724029115 | 0.2588 | T | A |
| rs798563 | 7 | 3.67003e-09 | C | A | 6.71084093935915e-05 | 34.8507962618638 | 0.4967 | C | A |
| rs62454712 | 7 | 2.63998e-14 | C | T | 0.000111821418560661 | 58.0737910523883 | 0.9908 | C | T |
| rs1474419 | 7 | 2.54976e-19 | C | T | 0.000155694207578475 | 80.8624120857688 | 0.3255 | C | T |
| rs73049276 | 7 | 5.12979e-22 | A | G | 0.000179415352876774 | 93.184599681803 | 0.5931 | A | G |
| rs12530608 | 7 | 3.63003e-09 | G | A | 6.7128348009577e-05 | 34.8611514960109 | 0.0310799 | G | A |
| rs7776857 | 7 | 3.16009e-13 | T | G | 0.000102400796594591 | 53.1807458111532 | 0.2596 | T | G |
| rs2158799 | 7 | 6.53131e-152 | G | C | 0.0013265539864128 | 689.775938409184 | 0.3284 | G | C |
| rs56388170 | 7 | 1e-200 | T | G | 0.00207393695966918 | 1079.20463040882 | 0.5199 | T | G |
| rs2710804 | 7 | 3.76011e-23 | C | T | 0.00018938426486806 | 98.3632258134862 | 0.2256 | C | T |
| rs3735485 | 7 | 4.19952e-28 | G | A | 0.000232830730772931 | 120.93389598753 | 0.9715 | G | A |
| rs10252457 | 7 | 1.82012e-12 | G | A | 9.57991197883278e-05 | 49.7519079073869 | 0.671301 | G | A |
| rs981132 | 7 | 7.87952e-25 | G | T | 0.000204118257685853 | 106.017393646331 | 0.8248 | G | T |
| rs778732 | 7 | 6.5298e-18 | T | C | 0.000143401365368612 | 74.4770015205119 | 0.659699 | T | C |
| rs62465399 | 7 | 1.65997e-12 | T | C | 9.61339051196947e-05 | 49.9257906152038 | 0.2642 | T | C |
| rs33951980 | 7 | 6.62979e-22 | T | C | 0.000178409462974946 | 92.6620681802298 | 0.4159 | T | C |
| rs76873475 | 7 | 1.17999e-08 | A | C | 6.27258692337737e-05 | 32.574709007867 | 0.0898794 | A | C |
| rs42033 | 7 | 5.50935e-59 | T | A | 0.000505055187999588 | 262.400615147569 | 5.45205e-07 | T | A |
| rs445 | 7 | 1.36144e-175 | T | C | 0.00153509435161226 | 798.378591938255 | 0.0183299 | T | C |
| rs7790229 | 7 | 1.45011e-12 | A | G | 9.66343032592012e-05 | 50.1856904614889 | 0.3462 | A | G |
| rs342242 | 7 | 3.61993e-16 | C | T | 0.000128122552198343 | 66.5407730145328 | 0.453401 | C | T |
| rs35759345 | 7 | 6.19013e-11 | T | C | 8.25024454175648e-05 | 42.8458997626121 | 0.2068 | T | C |
| rs9656395 | 7 | 4.08037e-12 | G | A | 9.27119575771153e-05 | 48.148485542739 | 0.8367 | G | A |
| rs7803075 | 7 | 2.18978e-18 | G | A | 0.000147557339210392 | 76.6357686193133 | 0.4726 | G | A |
| rs13231262 | 7 | 1.33999e-23 | T | C | 0.00019329514761729 | 100.394869866783 | 0.0418302 | T | C |
| rs12716647 | 8 | 1.02991e-12 | C | G | 9.79682878988147e-05 | 50.878544833752 | 0.7587 | C | G |
| rs6998846 | 8 | 4.54046e-11 | A | G | 8.36115678384605e-05 | 43.4219471936463 | 0.4687 | A | G |
| rs1991651 | 8 | 7.39946e-31 | G | C | 0.000257106015465172 | 133.545889798455 | 0.1417 | G | C |
| rs12550612 | 8 | 1.08993e-33 | A | G | 0.000281995895775127 | 146.477826879485 | 0.1372 | A | G |
| rs6468341 | 8 | 7.10068e-18 | C | T | 0.000143074257487816 | 74.3070903055894 | 6.60693e-05 | C | T |
| rs2923416 | 8 | 4.20998e-08 | G | A | 5.79555016016431e-05 | 30.097224904477 | 0.647201 | G | A |
| rs28571765 | 8 | 3.80014e-21 | C | T | 0.000171747935983796 | 89.2016188792106 | 0.9264 | C | T |
| rs7816785 | 8 | 4.31023e-27 | T | C | 0.000224087356455004 | 116.391508849625 | 0.622401 | T | C |
| rs609264 | 8 | 7.93049e-11 | G | T | 8.15420689423245e-05 | 42.3471078835745 | 0.00751294 | G | T |
| rs7846314 | 8 | 4.69894e-149 | T | A | 0.00130061829392196 | 676.272443689495 | 0.4075 | T | A |
| rs4276676 | 8 | 1.16011e-20 | C | T | 0.00016756779957632 | 87.0301958292005 | 0.1208 | C | T |
| rs16939607 | 8 | 1.99022e-25 | A | G | 0.000209341083407954 | 108.730655631832 | 0.2454 | A | G |
| rs7815046 | 8 | 4.64997e-10 | C | A | 7.48526200790208e-05 | 38.8728274033354 | 0.46 | C | A |
| rs73271394 | 8 | 3.39e-08 | C | T | 5.87644873685308e-05 | 30.5173689251956 | 0.4669 | C | T |
| rs10104995 | 8 | 3.3597e-22 | T | C | 0.000181059943037623 | 94.0389202617811 | 0.5264 | T | C |
| rs4734879 | 8 | 2.63027e-23 | G | A | 0.000190742791809846 | 99.0689580774132 | 0.5224 | G | A |
| rs28723563 | 8 | 5.54e-09 | C | T | 6.55839783011251e-05 | 34.0590754857457 | 0.3651 | C | T |
| rs2954038 | 8 | 2.63998e-23 | A | C | 0.000190775829144327 | 99.0861204498267 | 0.4848 | A | C |
| rs59697075 | 8 | 6.4003e-80 | T | C | 0.000689896599498959 | 358.500974170414 | 0.000596101 | T | C |
| rs145209947 | 8 | 1.9602e-14 | A | C | 0.000112940942992476 | 58.6552751048728 | 0.8578 | A | C |
| rs7005996 | 8 | 3.32966e-11 | T | C | 8.48152321210478e-05 | 44.0470984920933 | 0.8134 | T | C |
| rs6985508 | 8 | 3.3597e-39 | A | G | 0.000330606367140706 | 171.736034993665 | 0.732799 | A | G |
| rs2992836 | 9 | 6.64967e-29 | T | G | 0.000239884773604264 | 124.598693875336 | 0.3146 | T | G |
| rs2219143 | 9 | 1.29987e-20 | A | G | 0.000167121629863421 | 86.7984285801102 | 0.8814 | A | G |
| rs385893 | 9 | 7.70016e-34 | C | T | 0.0002835011355428 | 147.259919025742 | 0.1861 | C | T |
| rs36051895 | 9 | 1.75999e-09 | T | G | 6.98663792289947e-05 | 36.2831675778533 | 0.7295 | T | G |
| rs10811668 | 9 | 1.55998e-08 | A | C | 6.1689779684582e-05 | 32.0366152650254 | 0.742 | A | C |
| rs3739873 | 9 | 3.45001e-09 | A | G | 6.73271234342397e-05 | 34.9643866712498 | 0.5087 | A | G |
| rs3793537 | 9 | 2.55e-09 | C | G | 6.84504992672887e-05 | 35.5478192284874 | 0.4683 | C | G |
| rs10868825 | 9 | 2.59998e-08 | A | T | 5.9745450113114e-05 | 31.0268295193338 | 0.4391 | A | T |
| rs626416 | 9 | 5.77032e-18 | C | G | 0.000143778075259106 | 74.6726778829008 | 0.718701 | C | G |
| rs796007 | 9 | 9.41022e-21 | A | G | 0.000168275966581492 | 87.3980605753573 | 0.3775 | A | G |
| rs1288649 | 9 | 6.00994e-09 | G | T | 6.52229874352756e-05 | 33.8715934598292 | 0.159 | G | T |
| rs10992394 | 9 | 1.13999e-09 | A | G | 7.15280670486612e-05 | 37.1461808199434 | 0.681299 | A | G |
| rs10760690 | 9 | 1.84999e-08 | T | G | 6.10360663601188e-05 | 31.6971094227548 | 0.6955 | T | G |
| rs117468663 | 9 | 8.73977e-12 | T | A | 8.98428854977516e-05 | 46.6583445588931 | 0.587899 | T | A |
| rs1411424 | 9 | 2.50998e-09 | A | G | 6.85075202847963e-05 | 35.5774335003579 | 0.5747 | A | G |
| rs7866863 | 9 | 1e-10 | A | G | 8.06442005522022e-05 | 41.8807817701152 | 0.809 | A | G |
| rs17831500 | 9 | 5.64027e-18 | C | A | 0.00014396035627318 | 74.7673611036166 | 0.77 | C | A |
| rs12378064 | 9 | 1.99022e-11 | T | C | 8.67325985797432e-05 | 45.0429308765231 | 0.1477 | T | C |
| rs2519093 | 9 | 9.83105e-59 | T | C | 0.000503195612679836 | 261.433989362514 | 0.1611 | T | C |
| rs2157770 | 9 | 2.66993e-21 | G | A | 0.000173055813651871 | 89.8810156803257 | 0.000184001 | G | A |
| rs4413892 | 9 | 1.65006e-51 | A | G | 0.000439181406307021 | 228.16095980674 | 0.000201502 | A | G |
| rs9411293 | 9 | 4.18986e-11 | G | C | 8.38914516327773e-05 | 43.5673112775652 | 0.2141 | G | C |
| rs10906393 | 10 | 9.66051e-19 | T | A | 0.000150706647642718 | 78.2716482855149 | 0.9209 | T | A |
| rs692594 | 10 | 8.23e-11 | C | G | 8.14200472708749e-05 | 42.2837334106766 | 0.535499 | C | G |
| rs9804265 | 10 | 2.78997e-89 | C | A | 0.000772776683078936 | 401.602461667572 | 0.5196 | C | A |
| rs2807742 | 10 | 1.16011e-45 | A | G | 0.000387631037701873 | 201.369428084425 | 0.5986 | A | G |
| rs1571956 | 10 | 1.42988e-11 | A | G | 8.79794073970709e-05 | 45.6904943722139 | 0.1251 | A | G |
| rs72790862 | 10 | 6.70965e-24 | C | T | 0.000195890943800726 | 101.743355244388 | 0.9288 | C | T |
| rs10995477 | 10 | 2.86022e-56 | C | T | 0.000481503692093825 | 250.158578531806 | 0.02834 | C | T |
| rs138645114 | 10 | 2.03986e-14 | G | T | 0.000112809401315467 | 58.5869519304756 | 0.3142 | G | T |
| rs3747869 | 10 | 1.61994e-22 | C | A | 0.000183757432158365 | 95.4401997618218 | 0.07027 | C | A |
| rs2802372 | 10 | 7.44046e-12 | C | A | 9.04597202866042e-05 | 46.9787159902574 | 0.00843199 | C | A |
| rs1412445 | 10 | 2.63998e-25 | T | C | 0.000208214150680351 | 108.145210813416 | 0.836 | T | C |
| rs74149356 | 10 | 2.06001e-09 | T | A | 6.92683443461903e-05 | 35.9725732227448 | 0.1664 | T | A |
| rs1977289 | 10 | 2.13993e-21 | C | T | 0.000173879896516397 | 90.3090988791814 | 0.8257 | C | T |
| rs10786325 | 10 | 6.05341e-104 | G | C | 0.000902758857618042 | 469.213622891223 | 0.0857492 | G | C |
| rs7917772 | 10 | 5.41003e-16 | A | G | 0.000126530601180564 | 65.7138845819724 | 0.00742096 | A | G |
| rs10509912 | 10 | 7.0194e-14 | A | T | 0.000108073410759715 | 56.1270750242033 | 0.698601 | A | T |
| rs72836628 | 10 | 4.07005e-12 | T | C | 9.27121435097499e-05 | 48.1485821128586 | 0.1043 | T | C |
| rs180941 | 10 | 8.32914e-11 | A | G | 8.13360399393347e-05 | 42.2401024785996 | 0.3306 | A | G |
| rs3781454 | 10 | 6.26037e-31 | A | G | 0.000257855598965269 | 133.935338541221 | 0.4023 | A | G |
| rs9419387 | 10 | 2.65999e-10 | A | G | 7.6938505471049e-05 | 39.9561629194985 | 0.383 | A | G |
| rs14408 | 11 | 1.2106e-115 | C | T | 0.00100576880680982 | 522.807484072491 | 0.01085 | C | T |
| rs10833024 | 11 | 4.51024e-15 | T | C | 0.000118568254284998 | 61.5781357066921 | 0.6142 | T | C |
| rs4909932 | 11 | 8.35988e-12 | G | A | 9.00617927439294e-05 | 46.7720404806401 | 0.8584 | G | A |
| rs9988894 | 11 | 5.5195e-13 | G | C | 0.000100327407440062 | 52.1038455436613 | 0.7691 | G | C |
| rs1840595 | 11 | 1.29e-10 | T | C | 7.97051292797213e-05 | 41.393057002109 | 0.8147 | T | C |
| rs35043339 | 11 | 1.19001e-08 | A | C | 6.27020413291342e-05 | 32.5623339584504 | 0.8551 | A | C |
| rs58984522 | 11 | 4.70999e-08 | C | T | 5.75532257672062e-05 | 29.8883045640898 | 0.8221 | C | T |
| rs11823949 | 11 | 1.55991e-20 | A | G | 0.00016630244443873 | 86.3728951864131 | 0.5215 | A | G |
| rs7929589 | 11 | 1.79019e-14 | T | C | 0.000113296946967761 | 58.8401848163995 | 0.1905 | T | C |
| rs174557 | 11 | 3.19007e-31 | G | A | 0.000260418157232398 | 135.266729108913 | 0.4307 | G | A |
| rs7947929 | 11 | 1.38007e-14 | A | G | 0.000114268599225535 | 59.3448650720343 | 0.9507 | A | G |
| rs2282611 | 11 | 1.03001e-09 | G | T | 7.18637570781772e-05 | 37.3205249512374 | 0.0980302 | G | T |
| rs7131178 | 11 | 4.79005e-09 | T | A | 6.60724296962209e-05 | 34.3127548543146 | 0.3405 | T | A |
| rs61918362 | 11 | 2.90001e-08 | A | T | 5.93366454945272e-05 | 30.8145177223852 | 0.1377 | A | T |
| rs7934719 | 11 | 2.81968e-20 | T | C | 0.000164074305839771 | 85.2154716516694 | 0.1552 | T | C |
| rs61904448 | 11 | 2.36974e-11 | C | T | 8.60683036402405e-05 | 44.697912197605 | 0.690601 | C | T |
| rs73000965 | 11 | 6.62979e-45 | A | T | 0.000380861458244476 | 197.851377169966 | 0.5514 | A | T |
| rs1715460 | 11 | 4.32016e-19 | G | C | 0.000153735700574575 | 79.8450720466571 | 0.767499 | G | C |
| rs546039 | 11 | 1.94998e-08 | T | C | 6.08263303497444e-05 | 31.5881831752623 | 0.285 | T | C |
| rs8705 | 11 | 1.97015e-18 | A | G | 0.000147866981395645 | 76.796608983771 | 0.967 | A | G |
| rs470631 | 11 | 3.14e-08 | A | T | 5.90641887107183e-05 | 30.6730179757561 | 0.1628 | A | T |
| rs632887 | 12 | 1.05999e-12 | G | A | 9.7812119520555e-05 | 50.7974329019293 | 0.6009 | G | A |
| rs10849020 | 12 | 2.33991e-24 | G | C | 0.00019997528624961 | 103.865136955909 | 0.6417 | G | C |
| rs10846411 | 12 | 1.07994e-15 | G | A | 0.00012398838992689 | 64.3934190879293 | 0.0149899 | G | A |
| rs28588142 | 12 | 6.59994e-10 | T | C | 7.35696726208396e-05 | 38.2065118571546 | 0.6313 | T | C |
| rs7488780 | 12 | 1.12001e-08 | C | G | 6.28962158193909e-05 | 32.6631787183262 | 0.3165 | C | G |
| rs7969023 | 12 | 6.23003e-09 | C | T | 6.50827298957627e-05 | 33.7987501915805 | 0.8615 | C | T |
| rs739842 | 12 | 3.23996e-08 | C | T | 5.89361900792929e-05 | 30.6065422345065 | 0.7011 | C | T |
| rs12426444 | 12 | 6.6604e-19 | A | G | 0.000152012661926402 | 78.9500485681557 | 0.2357 | A | G |
| rs1700159 | 12 | 1.91999e-20 | T | C | 0.000165588399031271 | 86.0019783092545 | 0.1228 | T | C |
| rs1245035 | 12 | 3.39e-16 | A | C | 0.000128307765159225 | 66.6369761799637 | 0.2658 | A | C |
| rs1042725 | 12 | 3.32966e-11 | T | C | 8.47591131796802e-05 | 44.0179517691794 | 0.1332 | T | C |
| rs4761234 | 12 | 4.25011e-29 | C | T | 0.000241623958118638 | 125.502263069151 | 0.7905 | C | T |
| rs11104881 | 12 | 7.83971e-35 | C | T | 0.000292115772365927 | 151.73595543463 | 0.4984 | C | T |
| rs35864914 | 12 | 1.01e-08 | T | C | 6.33245648261121e-05 | 32.8856424392889 | 0.3485 | T | C |
| rs3184504 | 12 | 1.9002e-52 | C | T | 0.000447686372765304 | 232.581389286346 | 3.24496e-07 | C | T |
| rs11064881 | 12 | 2.36974e-20 | A | G | 0.000164768252614666 | 85.5759470265169 | 0.5662 | A | G |
| rs9165 | 12 | 7.32993e-14 | C | T | 0.000107902068336775 | 56.038080083077 | 0.6941 | C | T |
| rs370591186 | 12 | 1.89017e-13 | T | C | 0.000104355666205427 | 54.1960921504533 | 0.03271 | T | C |
| rs76603681 | 13 | 2.64972e-13 | A | G | 0.000103060654742311 | 53.5234713225144 | 0.8756 | A | G |
| rs9508005 | 13 | 2.18002e-08 | G | T | 6.04049083443147e-05 | 31.3693180952702 | 0.0112899 | G | T |
| rs78738581 | 13 | 1.13006e-20 | A | G | 0.000167633700734876 | 87.0644288522218 | 0.0253402 | A | G |
| rs7325851 | 13 | 6.32995e-13 | G | A | 9.98312457081864e-05 | 51.8461441239743 | 0.0464601 | G | A |
| rs2296028 | 13 | 2.15998e-09 | C | G | 6.9067710132334e-05 | 35.868372270118 | 0.6175 | C | G |
| rs9543219 | 13 | 5.06058e-11 | T | C | 8.32526138802117e-05 | 43.2355163211458 | 0.2914 | T | C |
| rs150861794 | 13 | 2.47002e-12 | T | C | 9.46315311659183e-05 | 49.1454800050478 | 0.0448601 | T | C |
| rs2260850 | 13 | 4.42996e-28 | G | T | 0.000232727404018114 | 120.880214861552 | 0.2093 | G | T |
| rs2038700 | 14 | 1.24997e-64 | C | T | 0.000555292519715576 | 288.51584208195 | 0.2473 | C | T |
| rs72664840 | 14 | 9.21086e-17 | T | C | 0.00013326891003215 | 69.2139032764038 | 0.0272797 | T | C |
| rs72731564 | 14 | 2.94985e-16 | T | C | 0.000128880881324342 | 66.9346639378716 | 0.4034 | T | C |
| rs34765661 | 14 | 8.58025e-27 | C | T | 0.000221362138690305 | 114.975710821178 | 0.0701795 | C | T |
| rs4903580 | 14 | 1.16011e-17 | T | C | 0.000141199590677086 | 73.3333252798536 | 0.672901 | T | C |
| rs149859245 | 14 | 1.40994e-14 | C | T | 0.000114230343825506 | 59.3249950383526 | 0.6413 | C | T |
| rs10498635 | 14 | 8.72971e-34 | T | C | 0.000282947904717133 | 146.972471201719 | 0.0156401 | T | C |
| rs12588718 | 14 | 4.29042e-12 | C | G | 9.25175399233061e-05 | 48.0475084738974 | 0.00567701 | C | G |
| rs2494748 | 14 | 1.5e-10 | T | C | 7.91359277644114e-05 | 41.097431668454 | 0.3254 | T | C |
| rs11625865 | 14 | 1.26009e-13 | A | G | 0.000105892157485698 | 54.9941383400763 | 0.2614 | A | G |
| rs4924450 | 15 | 3.40001e-10 | A | G | 7.6024499430602e-05 | 39.4814597695353 | 0.808 | A | G |
| rs72726027 | 15 | 1.71002e-45 | C | T | 0.00038625031753125 | 200.651884243535 | 0.8881 | C | T |
| rs12909505 | 15 | 2.99985e-28 | C | T | 0.00023411359126246 | 121.600378653663 | 0.05254 | C | T |
| rs780142 | 15 | 3.83999e-09 | G | T | 6.69338693296603e-05 | 34.7601478999194 | 0.8826 | G | T |
| rs62011281 | 15 | 7.03008e-09 | A | G | 6.46845848646069e-05 | 33.59197221878 | 0.0563703 | A | G |
| rs2062250 | 15 | 9.62055e-31 | A | G | 0.000256150296748048 | 133.049343625959 | 0.528 | A | G |
| rs7496362 | 15 | 4.25991e-18 | G | C | 0.000144974730304616 | 75.2942635665171 | 0.6169 | G | C |
| rs11072516 | 15 | 6.36063e-15 | A | T | 0.000117223324808619 | 60.879567951937 | 0.6896 | A | T |
| rs4886830 | 15 | 1.61002e-09 | T | A | 7.01797240967981e-05 | 36.4459059709554 | 0.00867801 | T | A |
| rs4843073 | 15 | 1.00995e-11 | T | C | 8.92642317511884e-05 | 46.3578039429023 | 0.1908 | T | C |
| rs7183988 | 15 | 6.26037e-18 | G | T | 0.000143518700930941 | 74.5379497213386 | 0.6592 | G | T |
| rs67175901 | 15 | 1.99022e-43 | T | C | 0.000367934613249409 | 191.133618249742 | 0.2845 | T | C |
| rs2955958 | 15 | 9.67008e-09 | A | G | 6.34553996351356e-05 | 32.9535917382642 | 0.01217 | A | G |
| rs4984803 | 16 | 1.33999e-17 | A | G | 0.000140682413760961 | 73.0646868287783 | 0.0861609 | A | G |
| rs35929659 | 16 | 1.21004e-13 | C | T | 0.000106084528145879 | 55.0940549095797 | 0.750601 | C | T |
| rs6500550 | 16 | 5.52968e-26 | T | C | 0.000214233344890141 | 111.272214953425 | 0.790801 | T | C |
| rs16958642 | 16 | 2.77e-10 | A | G | 7.68179743643527e-05 | 39.8935631784808 | 0.5457 | A | G |
| rs12923918 | 16 | 4.81005e-08 | G | A | 5.74856784260518e-05 | 29.8532241399936 | 0.0494094 | G | A |
| rs61739285 | 16 | 1.15001e-13 | T | C | 0.000106250611114392 | 55.1803177856344 | 0.6903 | T | C |
| rs11574938 | 16 | 1.57e-25 | C | G | 0.000210224771688438 | 109.189735178151 | 0.1585 | C | G |
| rs1362623 | 16 | 1.49005e-14 | T | C | 0.000113947997340255 | 59.1783430005023 | 0.6811 | T | C |
| rs11648664 | 16 | 5.43e-13 | A | G | 0.000100418772789144 | 52.1512998161105 | 0.1156 | A | G |
| rs75974417 | 16 | 2.32006e-12 | T | C | 9.48884159358884e-05 | 49.2789019546293 | 0.2085 | T | C |
| rs12927351 | 16 | 2.83009e-19 | A | C | 0.000155296675903406 | 80.6559152387045 | 0.9691 | A | C |
| rs12930850 | 16 | 1.29003e-22 | G | A | 0.000184708924867443 | 95.9344787131358 | 0.5739 | G | A |
| rs72832055 | 16 | 3.09001e-09 | A | G | 6.77429995686039e-05 | 35.1803744980765 | 0.4294 | A | G |
| rs305082 | 16 | 3.04018e-45 | C | T | 0.000383910490390777 | 199.43590845048 | 0.2876 | C | T |
| rs72803323 | 16 | 3.55959e-12 | T | C | 9.32666508121085e-05 | 48.4365835515412 | 0.0821505 | T | C |
| rs9905106 | 17 | 1.61994e-24 | C | T | 0.000201345746594319 | 104.577083516935 | 0.5486 | C | T |
| rs7225843 | 17 | 2.6903e-20 | C | T | 0.000164239408303165 | 85.301235204515 | 0.399 | C | T |
| rs78813154 | 17 | 7.13017e-12 | T | G | 9.0606004434645e-05 | 47.0546930565768 | 0.2956 | T | G |
| rs10852834 | 17 | 9.66941e-83 | G | C | 0.000715283419827612 | 371.702538611578 | 0.00297002 | G | C |
| rs16961474 | 17 | 1.10999e-09 | A | G | 7.15800664992012e-05 | 37.1731872713211 | 0.1221 | A | G |
| rs55767800 | 17 | 3.86999e-08 | C | T | 5.82596188449723e-05 | 30.2551670860328 | 0.0831591 | C | T |
| rs11653826 | 17 | 3.2802e-14 | T | C | 0.000111011333440163 | 57.6530314367051 | 0.1155 | T | C |
| rs2617884 | 17 | 1.05003e-24 | C | G | 0.000203063409586862 | 105.46940268724 | 0.3975 | C | G |
| rs11489942 | 17 | 9.1601e-36 | T | G | 0.000300356367249592 | 156.017717438408 | 0.3983 | T | G |
| rs4794822 | 17 | 1e-200 | T | C | 0.00717122529563478 | 3750.81483710811 | 0.0143999 | T | C |
| rs584438 | 17 | 6.68036e-11 | A | C | 8.21534341700369e-05 | 42.6646332625619 | 0.0270097 | A | C |
| rs56301633 | 17 | 1.13999e-24 | A | G | 0.00020275389670464 | 105.308611735552 | 0.5633 | A | G |
| rs56378716 | 17 | 3.71963e-50 | G | A | 0.000427307973396474 | 221.98990633015 | 0.5675 | G | A |
| rs113686433 | 17 | 8.30042e-13 | G | A | 9.87264046943732e-05 | 51.2723017181313 | 0.2236 | G | A |
| rs2665405 | 17 | 6.74062e-53 | A | G | 0.000451409416193244 | 234.516453032476 | 0.1456 | A | G |
| rs749780 | 17 | 4.93969e-18 | A | C | 0.000144458270878109 | 75.0259957768308 | 0.793 | A | C |
| rs820384 | 17 | 2.39999e-08 | C | G | 6.00681166950435e-05 | 31.1944058353123 | 0.3551 | C | G |
| rs2001613 | 17 | 2.12002e-09 | T | C | 6.91825999524956e-05 | 35.9280411942327 | 0.4745 | T | C |
| rs72901753 | 17 | 1.22999e-11 | C | G | 8.85536363768194e-05 | 45.9887360893861 | 0.223 | C | G |
| rs7235882 | 18 | 2.39994e-12 | T | C | 9.47162225786124e-05 | 49.1894673985 | 0.6531 | T | C |
| rs303753 | 18 | 9.56974e-29 | A | G | 0.000238497689190605 | 123.878055659048 | 0.0203901 | A | G |
| rs78285907 | 18 | 3.11e-10 | T | A | 7.63846442124014e-05 | 39.6685064192303 | 0.2511 | T | A |
| rs9965539 | 18 | 1.3499e-27 | A | G | 0.000228398034411683 | 118.630996783991 | 0.692599 | A | G |
| rs55874505 | 18 | 4.05976e-19 | G | T | 0.000153921888823504 | 79.9417867504147 | 0.431 | G | T |
| rs17742008 | 18 | 4.10015e-14 | A | C | 0.000110145340357548 | 57.2032338825754 | 0.1647 | A | C |
| rs2926999 | 18 | 1.15001e-14 | A | G | 0.000114963392943135 | 59.7057444428282 | 0.8973 | A | G |
| rs763362 | 18 | 2.22998e-15 | G | A | 0.000121182337431117 | 62.9359179969064 | 2.02302e-05 | G | A |
| rs72973711 | 18 | 5.52968e-21 | T | A | 0.000170317497039826 | 88.458557807981 | 0.1721 | T | A |
| rs12232734 | 18 | 9.42996e-10 | A | T | 7.2190807930683e-05 | 37.490382348084 | 0.3525 | A | T |
| rs930232 | 19 | 2.91005e-25 | A | G | 0.000207830869771573 | 107.946095571132 | 0.566899 | A | G |
| rs28540102 | 19 | 8.50942e-17 | C | T | 0.000133660066264183 | 69.4170794615033 | 3.79997e-05 | C | T |
| rs571497 | 19 | 9.75888e-45 | A | G | 0.000379574956434693 | 197.182806482326 | 0.1823 | A | G |
| rs10409243 | 19 | 5.55009e-26 | T | C | 0.000214155973895487 | 111.232020061876 | 0.00207802 | T | C |
| rs10412541 | 19 | 2.06001e-08 | A | G | 6.06486156528966e-05 | 31.4958872098881 | 0.0109099 | A | G |
| rs150665764 | 19 | 3.74999e-09 | C | G | 6.70056735230317e-05 | 34.7974398069722 | 0.2337 | C | G |
| rs4808683 | 19 | 3.67003e-10 | G | C | 7.57408503724237e-05 | 39.3341424278906 | 0.5203 | G | C |
| rs1985157 | 19 | 1.29003e-38 | C | T | 0.000325388834631874 | 169.024865184553 | 0.00547205 | C | T |
| rs56408111 | 19 | 1.10994e-18 | C | T | 0.000150128739982448 | 77.9714586273636 | 0.0950495 | C | T |
| rs4805881 | 19 | 3.05e-12 | C | A | 9.38174176069488e-05 | 48.7226425519452 | 0.1555 | C | A |
| rs8112528 | 19 | 2.35001e-08 | A | G | 6.01517042083097e-05 | 31.2378168794373 | 0.1004 | A | G |
| rs4760 | 19 | 1.41906e-171 | G | A | 0.00149986771295894 | 780.030247374763 | 0.9306 | G | A |
| rs11673093 | 19 | 1.44877e-109 | A | G | 0.000951778604305402 | 494.716164575983 | 0.9787 | A | G |
| rs386243 | 19 | 1.02991e-30 | T | C | 0.000255946400571183 | 132.943408954009 | 0.5535 | T | C |
| rs2254458 | 20 | 1.75999e-10 | T | C | 7.856617419589e-05 | 40.801519952811 | 0.5291 | T | C |
| rs156338 | 20 | 3.32001e-09 | T | C | 6.74553066747376e-05 | 35.0309594060076 | 0.4095 | T | C |
| rs633284 | 20 | 1.7398e-13 | T | A | 0.000104708796947249 | 54.379506344237 | 0.4922 | T | A |
| rs12481262 | 20 | 4.78961e-13 | C | T | 0.000100812937342058 | 52.3560251452905 | 0.3626 | C | T |
| rs4812447 | 20 | 1.49005e-18 | G | A | 0.000148945136108617 | 77.3566458454435 | 0.2399 | G | A |
| rs2179593 | 20 | 8.62005e-09 | A | C | 6.38640998248486e-05 | 33.1658510488685 | 0.2164 | A | C |
| rs1800961 | 20 | 8.99083e-21 | T | C | 0.000168461449824392 | 87.4944118689842 | 0.5847 | T | C |
| rs6067411 | 20 | 2.63998e-09 | T | C | 6.83173871188515e-05 | 35.4786864985599 | 0.00495998 | T | C |
| rs1555275 | 20 | 2.29985e-11 | G | A | 8.62084644039433e-05 | 44.7707082604755 | 0.6205 | G | A |
| rs2315008 | 20 | 1.63983e-15 | G | T | 0.000122319139264614 | 63.5263870451484 | 0.0564495 | G | T |
| rs62239920 | 21 | 1.55998e-10 | A | G | 7.89918957444179e-05 | 41.0226260285342 | 0.3043 | A | G |
| rs7283930 | 21 | 7.43002e-09 | T | C | 6.44219630768774e-05 | 33.455578792401 | 0.258 | T | C |
| rs9977672 | 21 | 7.34007e-24 | A | G | 0.000195552978307525 | 101.56778577641 | 0.231 | A | G |
| rs1788493 | 21 | 1.88018e-11 | T | C | 8.69445424812466e-05 | 45.1530094946688 | 0.8154 | T | C |
| rs2839166 | 21 | 1.90985e-11 | C | T | 8.69387440190923e-05 | 45.1499979108092 | 0.8248 | C | T |
| rs41433144 | 22 | 1.45011e-12 | A | T | 9.66618950766801e-05 | 50.2000212759783 | 0.2124 | A | T |
| rs5747308 | 22 | 2.29985e-30 | C | A | 0.000252785822530819 | 131.30132975338 | 0.9091 | C | A |
| rs9605901 | 22 | 1.29e-09 | T | C | 7.10389962089933e-05 | 36.8921769686031 | 0.4536 | T | C |
| rs2330721 | 22 | 3.58996e-10 | T | C | 7.58375897038397e-05 | 39.3843854238106 | 0.8238 | T | C |
| rs35955747 | 22 | 1.33999e-21 | T | A | 0.00017578897284755 | 91.3008022283566 | 0.3762 | T | A |
| rs139386 | 22 | 3.21004e-08 | C | T | 5.89813477022237e-05 | 30.6299947212657 | 0.1349 | C | T |
| rs47341 | 22 | 2.26001e-10 | T | C | 7.75577849657922e-05 | 40.2777957803704 | 0.8817 | T | C |
| rs738408 | 22 | 7.73927e-12 | T | C | 9.0331535531723e-05 | 46.9121394057139 | 0.9046 | T | C |

NETs and RA

| SNP | chr.exposure | pval.exposure | effect_allele.exposure | other_allele.exposure | r2 | F | pval.outcome | effect_allele.outcome | other_allele.outcome |
| --- | --- | --- | --- | --- | --- | --- | --- | --- | --- |
| rs62172784 | 2 | 2.08719530856408e-07 | A | G | 0.0384413089711633 | 26.1856687595129 | 0.6034 | G | A |
| rs10027231 | 4 | 1.76320924813796e-06 | A | C | 0.0316816487612366 | 21.4304313370316 | 0.7877 | C | A |
| rs2051756 | 4 | 2.59841118655236e-06 | T | C | 0.0320838107709936 | 21.7114831726704 | 0.609 | C | T |
| rs73329516 | 5 | 2.38991544596619e-06 | C | T | 0.0343363409953864 | 23.2899966176222 | 0.4667 | T | C |
| rs846294 | 7 | 1.07527486419377e-06 | G | C | 0.0366568914956012 | 24.923896499239 | 0.9799 | C | G |
| rs2738098 | 8 | 2.25071280466973e-06 | A | G | 0.0304889203263534 | 20.598261569619 | 0.490399 | G | A |
| rs3095792 | 10 | 4.6339639853318e-06 | A | T | 0.033400531476221 | 22.6333127932881 | 0.752 | T | A |
| rs12874659 | 13 | 1.95848959490628e-06 | T | C | 0.0327971565119692 | 22.2105814307458 | 0.2366 | C | T |
| rs17576011 | 14 | 1.04443984777173e-06 | T | C | 0.0351834224207366 | 23.8855158805449 | 0.2032 | C | T |
| rs2930850 | 15 | 1.11796124443004e-06 | A | G | 0.0346637080572131 | 23.5200198800982 | 0.155 | G | A |
| rs12926465 | 16 | 2.08265734263984e-06 | A | T | 0.0334618766158587 | 22.6763214539821 | 0.0206001 | T | A |
| rs12918182 | 16 | 2.4126998269673e-06 | T | A | 0.0352564573207442 | 23.9369101978691 | 0.917 | A | T |
| rs35659308 | 17 | 1.4693864747246e-06 | G | A | 0.0366568914956012 | 24.923896499239 | 0.0815098 | A | G |
| rs10406817 | 19 | 8.32553756014065e-07 | G | C | 0.0350118417174048 | 23.7648059492397 | 0.8925 | C | G |
| rs78230269 | 20 | 5.75551013771984e-07 | G | C | 0.0385197398451354 | 26.2412351497469 | 0.3038 | C | G |
| rs12482056 | 21 | 7.78820800250447e-08 | A | C | 0.0417907284233475 | 28.5667525135222 | 0.00240902 | C | A |

MPO-DNA and RA

| SNP | chr.exposure | pval.exposure | effect_allele.exposure | other_allele.exposure | r2 | F | pval.outcome | effect_allele.outcome | other_allele.outcome |
| --- | --- | --- | --- | --- | --- | --- | --- | --- | --- |
| rs147802074 | 1 | 1.42001e-06 | T | C | 0.00414393806109863 | 23.2526835658704 | 0.6501 | T | C |
| rs7615041 | 3 | 4.469e-06 | A | G | 0.00376930949318127 | 21.1425944297917 | 0.9643 | A | G |
| rs13314784 | 3 | 9.77597e-07 | C | T | 0.00425510051370651 | 23.8791096824687 | 0.8942 | C | T |
| rs289078 | 4 | 3.508e-07 | A | T | 0.00460280686518461 | 25.83941861605 | 0.0229599 | A | T |
| rs10457220 | 6 | 3.586e-06 | C | T | 0.00382093058764883 | 21.4332551038007 | 0.957 | C | T |
| rs67983734 | 8 | 4.17696e-06 | T | C | 0.0037748940783987 | 21.1740378602263 | 0.764499 | T | C |
| rs10985542 | 9 | 2.83602e-06 | A | G | 0.00391234690484426 | 21.9480629403819 | 0.3199 | A | G |

MPO and RA

| SNP | chr.exposure | pval.exposure | effect_allele.exposure | other_allele.exposure | r2 | F | pval.outcome | effect_allele.outcome | other_allele.outcome |
| --- | --- | --- | --- | --- | --- | --- | --- | --- | --- |
| rs10103048 | 8 | 2.30403992296525e-08 | A | C | 0.00581143633813519 | 31.302152054626 | 0.00111199 | C | A |
| rs12948186 | 17 | 3.56943929198845e-55 | G | A | 0.0446709725486436 | 250.398607311413 | 0.8563 | G | A |
| rs139237904 | 17 | 5.31740818827143e-15 | T | C | 0.0113492512569143 | 61.4729120046107 | 0.8202 | T | C |
| rs3744376 | 17 | 2.50149707846009e-12 | A | G | 0.00911453324852867 | 49.2572826866507 | 0.9567 | A | G |

IL-18 and RA

| SNP | chr.exposure | pval.exposure | effect_allele.exposure | other_allele.exposure | r2 | F | pval.outcome | effect_allele.outcome | other_allele.outcome |
| --- | --- | --- | --- | --- | --- | --- | --- | --- | --- |
| rs385076 | 2 | 1.65882e-22 | C | T | 0.0257669354811728 | 96.1135963752462 | 0.8926 | C | T |
| rs1656939 | 3 | 1.56401e-06 | T | A | 0.00628789892129272 | 22.9948137445172 | 0.9262 | T | A |
| rs4482818 | 4 | 1.45301e-07 | G | A | 0.00758181396708661 | 27.7628043743638 | 0.8689 | G | A |
| rs4414903 | 4 | 3.30697e-06 | C | G | 0.00589647094822004 | 21.5548731089109 | 0.1796 | C | G |
| rs116383510 | 5 | 2.99502e-07 | C | A | 0.00720884630987467 | 26.3871685325887 | 0.750601 | C | A |
| rs658805 | 6 | 4.94197e-07 | A | G | 0.00689560407144325 | 25.2326193483363 | 0.751201 | A | G |
| rs117266781 | 7 | 3.15e-06 | T | C | 0.00593713664787904 | 21.704416666001 | 0.1225 | T | C |
| rs1852105 | 7 | 4.31996e-06 | C | T | 0.00576851799469357 | 21.0844202503382 | 0.1577 | C | T |
| rs78623212 | 7 | 6.71104e-07 | T | C | 0.00654932395316148 | 23.9571463582825 | 0.1781 | T | C |
| rs2729385 | 11 | 3.78504e-06 | A | G | 0.00603477163701416 | 22.0635083633939 | 0.0575201 | A | G |
| rs71478720 | 11 | 3.70766e-22 | T | C | 0.0250741767416786 | 93.4630677590706 | 0.3809 | T | C |
| rs1979967 | 15 | 9.45301e-07 | T | C | 0.00656567299714813 | 24.0173457098265 | 0.786799 | T | C |
| rs143370787 | 16 | 2.35402e-06 | G | C | 0.00609296825692669 | 22.2775832532748 | 0.4902 | G | C |
| rs10414578 | 19 | 4.15901e-07 | T | C | 0.00699245543673816 | 25.5895166116612 | 0.670101 | T | C |
| rs78716465 | 20 | 1.63099e-06 | A | G | 0.00626389997861911 | 22.9064965253975 | 0.4118 | A | G |
| rs11700536 | 21 | 4.20901e-06 | T | C | 0.00584608871809381 | 21.3696150671067 | 0.9035 | T | C |

IL-13 and RA

| SNP | chr.exposure | pval.exposure | effect_allele.exposure | other_allele.exposure | r2 | F | pval.outcome | effect_allele.outcome | other_allele.outcome |
| --- | --- | --- | --- | --- | --- | --- | --- | --- | --- |
| rs75383097 | 1 | 4.28499e-06 | C | G | 0.00591584809251679 | 21.1559956252622 | 0.4897 | C | G |
| rs12623722 | 2 | 4.19199e-06 | A | G | 0.00589583494639825 | 21.0840010244959 | 0.0666101 | A | G |
| rs6799107 | 3 | 1.245e-06 | C | T | 0.00656197402512786 | 23.4819053120477 | 0.584501 | C | T |
| rs139083458 | 5 | 2.81002e-06 | T | C | 0.00617084503909053 | 22.0735666733682 | 0.246 | T | C |
| rs27949 | 5 | 3.42799e-06 | T | C | 0.00600324002039851 | 21.4704102988773 | 0.593 | T | C |
| rs75995699 | 6 | 2.64198e-06 | A | G | 0.00631637531131307 | 22.5974481955993 | 0.3063 | A | G |
| rs9472168 | 6 | 1.08094e-65 | G | A | 0.0760682483728949 | 292.686794765316 | 0.6691 | G | A |
| rs142167313 | 6 | 3.97997e-07 | C | T | 0.00718296420933765 | 25.7201849320196 | 0.6975 | C | T |
| rs117795020 | 9 | 9.86211e-07 | A | G | 0.00675654240093602 | 24.1829010315247 | 0.619699 | A | G |
| rs7073807 | 10 | 2.374e-06 | C | T | 0.00623664608306545 | 22.3104190126444 | 0.712801 | C | T |
| rs76339001 | 21 | 1.15199e-06 | T | A | 0.00661795656805323 | 23.6835724533015 | 0.897 | T | A |

IL-6 and RA

| SNP | chr.exposure | pval.exposure | effect_allele.exposure | other_allele.exposure | r2 | F | pval.outcome | effect_allele.outcome | other_allele.outcome |
| --- | --- | --- | --- | --- | --- | --- | --- | --- | --- |
| rs10752777 | 1 | 3.24698e-06 | A | T | 0.00263635165060126 | 21.6408638907112 | 0.774599 | A | T |
| rs13412535 | 2 | 7.34294e-08 | A | G | 0.00356653487049922 | 29.3037337731148 | 0.559 | A | G |
| rs75101555 | 3 | 2.59597e-06 | G | C | 0.00268485783217405 | 22.0401056222107 | 0.8223 | G | C |
| rs1333040 | 9 | 3.16804e-06 | T | C | 0.00265712611362448 | 21.8118483240118 | 0.1227 | T | C |
| rs76856708 | 16 | 2.61198e-06 | C | T | 0.00268863324703594 | 22.0711817064205 | 0.341 | C | T |
| rs73273528 | 20 | 9.58098e-07 | T | C | 0.00284285937543953 | 23.3408444451851 | 0.159 | T | C |
| rs1884910 | 20 | 3.26002e-06 | G | C | 0.00266129229752801 | 21.8461390013167 | 0.719 | G | C |

IL-5 and RA

| SNP | chr.exposure | pval.exposure | effect_allele.exposure | other_allele.exposure | r2 | F | pval.outcome | effect_allele.outcome | other_allele.outcome |
| --- | --- | --- | --- | --- | --- | --- | --- | --- | --- |
| rs6737109 | 2 | 2.399e-06 | C | T | 0.00651370320311355 | 22.0426494451638 | 0.0849591 | C | T |
| rs11680908 | 2 | 2.03002e-06 | G | A | 0.00667494077227265 | 22.5919508099673 | 0.09769 | G | A |
| rs7578892 | 2 | 2.43703e-06 | G | C | 0.00655368773914127 | 22.1788514457814 | 0.8518 | G | C |
| rs7767396 | 6 | 7.69006e-10 | G | A | 0.0111488478401837 | 37.9050237812128 | 0.6278 | G | A |
| rs73040130 | 19 | 6.00399e-07 | C | T | 0.0073380996423231 | 24.8530652668355 | 0.2058 | C | T |

IL-4 and RA

| SNP | chr.exposure | pval.exposure | effect_allele.exposure | other_allele.exposure | r2 | F | pval.outcome | effect_allele.outcome | other_allele.outcome |
| --- | --- | --- | --- | --- | --- | --- | --- | --- | --- |
| rs116705532 | 1 | 1.75501e-06 | G | T | 0.00280835136499153 | 22.8736670806295 | 0.1196 | G | T |
| rs79597994 | 1 | 4.31698e-06 | T | C | 0.00258811318875512 | 21.0752003229806 | 0.288 | T | C |
| rs4444693 | 3 | 1.70098e-06 | A | T | 0.00281398924572108 | 22.9197164894629 | 0.7487 | A | T |
| rs7613691 | 3 | 4.048e-06 | G | A | 0.00262315306826184 | 21.3612831358224 | 0.678 | G | A |
| rs13106889 | 4 | 2.27201e-07 | A | T | 0.00326669403143729 | 26.6190451994092 | 0.8088 | A | T |
| rs17713451 | 7 | 4.97004e-07 | A | G | 0.00311153342315192 | 25.3507541817786 | 0.0419199 | A | G |
| rs10512267 | 9 | 2.93697e-07 | C | T | 0.00321391748356229 | 26.1876025953266 | 0.4629 | C | T |
| rs117146485 | 9 | 2.70901e-06 | C | T | 0.00265295471216912 | 21.6046142353779 | 0.8239 | C | T |
| rs9508291 | 13 | 3.03201e-06 | C | T | 0.00267562748858581 | 21.7897477102368 | 0.0965206 | C | T |
| rs2849346 | 18 | 2.86702e-06 | G | C | 0.00267350501964208 | 21.7724164341594 | 0.4766 | G | C |
| rs9941733 | 20 | 6.88098e-07 | G | A | 0.00304120734864651 | 24.7760351458629 | 0.5329 | G | A |

IL-1β and RA

| SNP | chr.exposure | pval.exposure | effect_allele.exposure | other_allele.exposure | r2 | F | pval.outcome | effect_allele.outcome | other_allele.outcome |
| --- | --- | --- | --- | --- | --- | --- | --- | --- | --- |
| rs143319329 | 7 | 1.996e-06 | T | C | 0.00461644671917719 | 15.3373935605021 | 0.3168 | T | C |
| rs61335305 | 15 | 1.89601e-06 | A | C | 0.00504627918683254 | 16.7726849216828 | 0.1961 | A | C |
| rs62015704 | 16 | 2.08699e-06 | G | A | 0.00439815545735118 | 14.6089525418083 | 0.843 | G | A |
| rs9898641 | 17 | 3.59203e-06 | C | T | 0.00601752167375123 | 20.0204174711455 | 0.7669 | C | T |
| rs1942793 | 18 | 4.976e-06 | T | G | 0.00442316473238334 | 14.6923926429644 | 0.5027 | T | G |

**Table S4. Results of heterogeneity and pleiotropy tests for instrumental variables**

| **Exposure** | **Outcome** | **Heterogeneity** | | **Pleiotropy** | |
| --- | --- | --- | --- | --- | --- |
|  |  | **Q statistic (IVW)** | **P** | **MR-Egger intercept** | **P** |
| Neutrophil extracellular traps measurement | Rheumatoid arthritis | 17.052 | 0.073 | 0.014 | 0.629 |
| Interleukin-6 levels |  | 3.968 | 0.410 | -0.021 | 0.322 |
| Interleukin-18 levels |  | 13.740 | 0.469 | -0.003 | 0.771 |
| Interleukin-1β levels |  | 2.980 | 0.561 | 0.013 | 0.493 |
| TNF-a levels |  | 5.413 | 0.247 | 0.025 | 0.273 |
| Neutrophil count |  | 4546.517 | <0.001 | -0.008 | 0.118 |
| Neutrophil count (Outlier corrected) |  | 539.459 | <0.001 | -0.001 | 0.464 |
| Interleukin-4 levels |  | 11.646 | 0.168 | 0.025 | 0.282 |
| Interleukin-5 levels |  | 4.168 | 0.244 | -0.008 | 0.897 |
| Interleukin-13 levels |  | 5.492 | 0.704 | -0.008 | 0.533 |
| Blood protein levels of myeloperoxidase |  | 9.257 | 0.026 | -0.048 | 0.096 |
| Blood protein levels of myeloperoxidase-DNA complexes |  | 4.156 | 0.656 | 0 | 0.990 |

**Table S5. Results of MR-PRESSO Test**

| **Exposure** | **Outcome** | **Raw** | | **Outlier corrected** | | **Global P** | Outliers, n | **Distortion P** |  |
| --- | --- | --- | --- | --- | --- | --- | --- | --- | --- |
|  |  | **OR (CI%)** | **P** | **OR (CI%)** | **P** |  |  |  |  |
| Neutrophil extracellular traps measurement | Rheumatoid arthritis | 1.00 (0.98-1.01) | 0.75 | NA | NA | 0.039 | 0 | NA |  |
| Interleukin-6 levels |  | 0.94 (0.84-1.04) | 0.26 | NA | NA | 0.598 | 0 | NA |  |
| Interleukin-18 levels |  | 1.01 (0.97-1.06) | 0.59 | NA | NA | 0.551 | 0 | NA |  |
| Interleukin-1β levels |  | 0.97 (0.90-1.06) | 0.57 | NA | NA | 0.63 | 0 | NA |  |
| TNF-a levels |  | 1.04 (0.93-1.17) | 0.54 | NA | NA | 0.334 | 0 | NA |  |
| Neutrophil count |  | 1.21 (0.98-1.50) | 0.07 | 1.05 (0.97-1.14) | 0.24 | <0.001 | 22 | <0.001 |  |
| Neutrophil count(Outlier corrected) |  | 1.09 (1 - 1.18) | 0.06 |  | NA | NA | <0.001 | 0 | NA |
| Interleukin-4 levels |  | 1.00 (0.89-1.13) | 0.98 | NA | NA | 0.287 | 0 | NA |  |
| Interleukin-5 levels |  | 0.92 (0.82 – 1.04) | 0.26 | NA | NA | 0.382 | 0 | NA |  |
| Interleukin-13 levels |  | 0.96 (0.93 – 1) | 0.11 | NA | NA | 0.693 | 0 | NA |  |
| Blood protein levels of myeloperoxidase |  | 0.95 (0.81-1.1) | 0.54 | NA | NA | 0.098 | 0 | NA |  |
| Blood protein levels of myeloperoxidase-DNA complexes |  | 0.89 (0.78-1.01) | 0.12 | NA | NA | 0.643 | 0 | NA |  |

**Table S6. IV selection for RA as exposure and NETs as outcomes**

| **Exposure** | **Outcomes** | **IVs, n** | **Mean F** | **F range** | **Lacking SNPs** | **Proxies, n** | **R^2^** |
| --- | --- | --- | --- | --- | --- | --- | --- |
| Rheumatoid arthritis | Neutrophil extracellular traps measurement | 90 | 107.49 | 29.98-1487.90 | 17 | 0 | 0.16 |
|  | Interleukin-6 levels | 90 | 107.49 | 29.98-1487.90 | 4 | 0 | 0.16 |
|  | Interleukin-18 levels | 90 | 107.49 | 29.98-1487.90 | 4 | 0 | 0.16 |
|  | Interleukin-1β levels | 90 | 107.49 | 29.98-1487.90 | 4 | 0 | 0.16 |
|  | TNF-a levels | 90 | 107.49 | 29.98-1487.90 | 4 | 0 | 0.16 |
|  | Neutrophil count | 90 | 107.49 | 29.98-1487.90 | 4 | 0 | 0.16 |
|  | Interleukin-4 levels | 90 | 107.49 | 29.98-1487.90 | 4 | 0 | 0.16 |
|  | Interleukin-5 levels | 90 | 107.49 | 29.98-1487.90 | 4 | 0 | 0.16 |
|  | Interleukin-13 levels | 90 | 107.49 | 29.98-1487.90 | 4 | 0 | 0.16 |
|  | Blood protein levels of myeloperoxidase | 90 | 107.49 | 29.98-1487.90 | 14 | 0 | 0.16 |
|  | Blood protein levels of myeloperoxidase-DNA complexes | 90 | 107.49 | 29.98-1487.90 | 14 | 0 | 0.16 |

​​**Supplementary Table 7. Detailed information on IV for RA as exposure and NETs as outcomes**

**RA and TNF-a**

| SNP | chr.exposure | pval.exposure | effect_allele.exposure | other_allele.exposure | r2 | F | pval.outcome | effect_allele.outcome | other_allele.outcome |
| --- | --- | --- | --- | --- | --- | --- | --- | --- | --- |
| rs2258734 | 1 | 6.04366e-14 | A | G | 0.000961041434499621 | 56.0652979598839 | 0.289 | A | G |
| rs2076616 | 1 | 6.20297e-11 | G | A | 0.000736799884008522 | 42.9738339556586 | 0.0467401 | G | A |
| rs2301888 | 1 | 3.75232e-26 | A | G | 0.0019222962296568 | 112.251048624403 | 0.1136 | A | G |
| rs28411352 | 1 | 1.66418e-11 | T | C | 0.000774334849760245 | 45.164756358556 | 0.4096 | T | C |
| rs6679677 | 1 | 1.40929e-145 | A | C | 0.0112015396813439 | 660.243883771518 | 0.2881 | A | C |
| rs12126142 | 1 | 1.013e-10 | A | G | 0.00071862502862948 | 41.9130236664156 | 0.6261 | A | G |
| rs3761959 | 1 | 9.64495e-11 | T | C | 0.000717610978962601 | 41.8538378494508 | 0.1063 | T | C |
| rs1234313 | 1 | 1.89701e-09 | G | A | 0.000615738794392477 | 35.9085987320739 | 0.8641 | G | A |
| rs61828284 | 1 | 6.32805e-09 | T | C | 0.000576612422506277 | 33.6255140976527 | 0.1263 | T | C |
| rs10911902 | 1 | 2.357e-08 | T | C | 0.00053247470649769 | 31.0502243031709 | 0.4834 | T | C |
| rs3134883 | 10 | 1.98381e-15 | A | G | 0.00107723348509969 | 62.851027209663 | 0.3566 | A | G |
| rs502919 | 10 | 6.17007e-10 | C | T | 0.000656243505366125 | 38.2722999280117 | 0.587801 | C | T |
| rs2275806 | 10 | 2.51397e-09 | A | G | 0.000605541537591372 | 35.3135557187281 | 0.5104 | A | G |
| rs1538981 | 10 | 4.41601e-09 | T | C | 0.000594056723991988 | 34.6433941289253 | 0.3743 | T | C |
| rs7097397 | 10 | 1.41612e-12 | A | G | 0.000854051207089392 | 49.8183598819764 | 0.742 | A | G |
| rs71508903 | 10 | 3.12608e-25 | T | C | 0.00185180621703494 | 108.127200563464 | 0.186 | T | C |
| rs6479800 | 10 | 3.01093e-11 | C | G | 0.000756090786340865 | 44.0998266821519 | 0.3371 | C | G |
| rs9943599 | 11 | 2.70402e-10 | T | C | 0.00068828011840664 | 40.1419707813783 | 0.8761 | T | C |
| rs660442 | 11 | 1.11301e-09 | A | G | 0.000637421158338194 | 37.1738753649617 | 0.9149 | A | G |
| rs34502849 | 11 | 1.068e-09 | A | G | 0.000633546456743195 | 36.9477627160603 | 0.9387 | A | G |
| rs4409785 | 11 | 7.84802e-09 | C | T | 0.000572172816140967 | 33.3664674559768 | 0.2467 | C | T |
| rs6421571 | 11 | 5.57057e-14 | C | T | 0.000971400144704648 | 56.6701926670335 | 0.00829698 | C | T |
| rs7105899 | 11 | 4.63895e-10 | A | G | 0.000664750328436899 | 38.7687501813755 | 0.4338 | A | G |
| rs4963581 | 12 | 3.75301e-08 | A | G | 0.00051632682328896 | 30.1081055374145 | 0.9271 | A | G |
| rs4622308 | 12 | 2.20699e-12 | T | C | 0.000845769827270578 | 49.3348830284812 | 0.8738 | T | C |
| rs9532434 | 13 | 1.94089e-19 | C | T | 0.00140252214664057 | 81.8566014468835 | 0.8816 | C | T |
| rs1950897 | 14 | 1.02212e-13 | T | C | 0.000944649342905473 | 55.1081108439142 | 0.9675 | T | C |
| rs1595260 | 14 | 2.28613e-11 | T | A | 0.00077105965920406 | 44.9735763681987 | 0.6758 | T | A |
| rs2841275 | 14 | 1.7108e-19 | C | A | 0.00139816037628769 | 81.601675279814 | 0.8118 | C | A |
| rs8032939 | 15 | 4.46581e-24 | C | T | 0.00175194175045908 | 102.285867982857 | 0.4636 | C | T |
| rs7170107 | 15 | 6.11223e-18 | T | C | 0.00128079986879687 | 74.7433091738027 | 0.4438 | T | C |
| rs7206670 | 16 | 4.142e-09 | T | G | 0.000595023709630473 | 34.6998190597437 | 0.3998 | T | G |
| rs12918327 | 16 | 3.04299e-08 | T | C | 0.00052295264721102 | 30.4946734549618 | 0.335 | T | C |
| rs9927316 | 16 | 2.30303e-11 | G | C | 0.000760849344255047 | 44.3775861391863 | 0.702 | G | C |
| rs4795400 | 17 | 5.85504e-10 | T | C | 0.000657325104866287 | 38.3354205961804 | 0.8966 | T | C |
| rs2847297 | 18 | 2.64972e-14 | G | A | 0.000986968703644098 | 57.5793389913463 | 0.2503 | G | A |
| rs34536443 | 19 | 1.07895e-15 | C | G | 0.00110207415477557 | 64.3019514073775 | 0.9687 | C | G |
| rs1355208 | 2 | 6.76862e-12 | G | A | 0.000810049190921235 | 47.2495614142664 | 0.9224 | G | A |
| rs12466919 | 2 | 1.58891e-11 | T | C | 0.000779601196224082 | 45.4721670741779 | 0.4267 | T | C |
| rs1858037 | 2 | 1.14393e-14 | A | T | 0.00102287985551872 | 59.6765256552822 | 0.3763 | A | T |
| rs28421442 | 2 | 7.86303e-09 | A | T | 0.000570171795089792 | 33.2497106086072 | 0.3829 | A | T |
| rs11123811 | 2 | 2.01094e-18 | C | T | 0.00130532856512629 | 76.1765949180311 | 0.9801 | C | T |
| rs11889341 | 2 | 4.32215e-30 | T | C | 0.00221094483306058 | 129.143816614502 | 0.3458 | T | C |
| rs3087243 | 2 | 3.31513e-24 | A | G | 0.00177119977751859 | 103.412229150603 | 0.0335097 | A | G |
| rs1883832 | 20 | 1.13006e-16 | C | T | 0.00117588190877531 | 68.6134306991008 | 0.8667 | C | T |
| rs6011186 | 20 | 3.19396e-10 | T | C | 0.000676352589078809 | 39.4458609068439 | 0.5263 | T | C |
| rs8126756 | 21 | 1.81401e-09 | C | T | 0.00061878584168435 | 36.0864061822702 | 0.7019 | C | T |
| rs1893592 | 21 | 1.48115e-13 | C | A | 0.000937120085919884 | 54.6684637630414 | 0.2391 | C | A |
| rs7278257 | 21 | 4.27504e-10 | C | G | 0.000670135275503143 | 39.0830150339214 | 0.9315 | C | G |
| rs2073609 | 21 | 1.46501e-08 | C | T | 0.000548151364659465 | 31.9648794273622 | 0.9051 | C | T |
| rs5754104 | 22 | 1.355e-10 | A | G | 0.000704482804846108 | 41.0876123484325 | 0.749401 | A | G |
| rs2069235 | 22 | 1.69005e-20 | A | G | 0.0014681367501917 | 85.6917532868614 | 0.0229103 | A | G |
| rs4602367 | 3 | 1.75699e-10 | G | A | 0.000704523323802617 | 41.0899772051797 | 0.0580403 | G | A |
| rs3806624 | 3 | 3.93641e-11 | G | A | 0.00074405731438719 | 43.3974385787144 | 0.1576 | G | A |
| rs13103285 | 4 | 4.29042e-14 | T | C | 0.00097695857072941 | 56.9947809589962 | 0.1979 | T | C |
| rs34046593 | 4 | 7.16638e-17 | A | G | 0.00119903249995213 | 69.9659035544603 | 0.6107 | A | G |
| rs2918392 | 5 | 4.62104e-08 | C | T | 0.000514115359899186 | 29.9790841132627 | 0.0989692 | C | T |
| rs7731626 | 5 | 1.94089e-26 | A | G | 0.00193513591072256 | 113.002265891452 | 0.6506 | A | G |
| rs403214 | 5 | 3.95804e-10 | G | A | 0.000671963065116686 | 39.1896853822411 | 0.4332 | G | A |
| rs244685 | 5 | 6.03601e-10 | G | T | 0.00065496959057473 | 38.1979561775948 | 0.1043 | G | T |
| rs9405192 | 6 | 9.25977e-11 | A | G | 0.000723561044714213 | 42.2011198944327 | 0.00570598 | A | G |
| rs12530098 | 6 | 1.35394e-11 | T | C | 0.000786800385694845 | 45.8924082435734 | 0.781399 | T | C |
| rs3025669 | 6 | 2.91273e-30 | G | C | 0.00223042784521827 | 130.284385596443 | 0.567599 | G | C |
| rs1611236 | 6 | 4.5436e-19 | A | G | 0.00135509891890628 | 79.0850432483033 | 0.0616098 | A | G |
| rs9258357 | 6 | 5.76501e-22 | C | T | 0.00159842263977987 | 93.3084145739876 | 0.0617007 | C | T |
| rs11754264 | 6 | 1.88105e-12 | C | T | 0.000849974017077673 | 49.5803276535847 | 0.692201 | C | T |
| rs112733823 | 6 | 3.82032e-24 | T | C | 0.00176780053508964 | 103.21341151019 | 0.8449 | T | C |
| rs139395255 | 6 | 8.6836e-60 | G | A | 0.00454375326935948 | 266.027792697619 | 0.710101 | G | A |
| rs114508013 | 6 | 1.81593e-28 | A | G | 0.00209653273183503 | 122.446834473192 | 0.5345 | A | G |
| rs115521560 | 6 | 1.29122e-103 | C | A | 0.0079726642579076 | 468.397191829171 | 0.2787 | C | A |
| rs71565312 | 6 | 1.1051e-67 | A | G | 0.00516066708279514 | 302.334245307225 | 0.9961 | A | G |
| rs146305655 | 6 | 3.29079e-22 | A | G | 0.00160777348524116 | 93.8551520918116 | 0.3937 | A | G |
| rs5020946 | 6 | 1e-200 | T | G | 0.0248937958134512 | 1487.89967838416 | 0.7489 | T | G |
| rs9271365 | 6 | 1e-200 | G | T | 0.0244095978658332 | 1458.23511558168 | 0.718701 | G | T |
| rs2233424 | 6 | 6.49232e-26 | T | C | 0.0018889882274731 | 110.302371755295 | 0.617401 | T | C |
| rs76153210 | 6 | 6.83282e-15 | T | C | 0.00104016153387336 | 60.6858175702955 | 0.525801 | T | C |
| rs62422878 | 6 | 3.57396e-09 | T | C | 0.000595284016876986 | 34.7150083612477 | 0.702499 | T | C |
| rs7749323 | 6 | 3.46817e-29 | A | G | 0.0021497161530944 | 125.559674495087 | 0.7845 | A | G |
| rs212389 | 6 | 6.65733e-13 | A | G | 0.000887976559838028 | 51.7990461993269 | 0.8328 | A | G |
| rs1571878 | 6 | 4.12572e-40 | T | C | 0.00301094140472042 | 176.013653747781 | 0.8879 | T | C |
| rs740122 | 7 | 5.37205e-09 | A | G | 0.000583983887497936 | 34.0556368743678 | 0.8129 | A | G |
| rs42034 | 7 | 1.28499e-08 | G | A | 0.000555728515629577 | 32.4069788301643 | 0.9353 | G | A |
| rs3757387 | 7 | 1.86681e-19 | C | T | 0.00139457020037203 | 81.3918470625492 | 0.7648 | C | T |
| rs9693589 | 8 | 1.49589e-18 | A | G | 0.00132831561999551 | 77.5198618078778 | 0.5083 | A | G |
| rs11574914 | 9 | 9.91745e-15 | A | G | 0.00102633884407141 | 59.8785361777755 | 0.0157 | A | G |
| rs10435844 | 9 | 9.72971e-11 | T | G | 0.000719779256577692 | 41.9803912466634 | 0.341 | T | G |

**RA_Neutrophil count**

| SNP | chr.exposure | pval.exposure | effect_allele.exposure | other_allele.exposure | r2 | F | pval.outcome | effect_allele.outcome | other_allele.outcome |
| --- | --- | --- | --- | --- | --- | --- | --- | --- | --- |
| rs2258734 | 1 | 6.04366e-14 | A | G | 0.000961041434499621 | 56.0652979598839 | 0.321062 | A | G |
| rs2076616 | 1 | 6.20297e-11 | G | A | 0.000736799884008522 | 42.9738339556586 | 0.00604895 | G | A |
| rs2301888 | 1 | 3.75232e-26 | A | G | 0.0019222962296568 | 112.251048624403 | 0.00323497 | A | G |
| rs28411352 | 1 | 1.66418e-11 | T | C | 0.000774334849760245 | 45.164756358556 | 0.001695 | T | C |
| rs12126142 | 1 | 1.013e-10 | A | G | 0.00071862502862948 | 41.9130236664156 | 0.970404 | A | G |
| rs3761959 | 1 | 9.64495e-11 | T | C | 0.000717610978962601 | 41.8538378494508 | 0.79995 | T | C |
| rs1234313 | 1 | 1.89701e-09 | G | A | 0.000615738794392477 | 35.9085987320739 | 0.963073 | G | A |
| rs61828284 | 1 | 6.32805e-09 | T | C | 0.000576612422506277 | 33.6255140976527 | 0.0354969 | T | C |
| rs10911902 | 1 | 2.357e-08 | T | C | 0.00053247470649769 | 31.0502243031709 | 0.919803 | T | C |
| rs3134883 | 10 | 1.98381e-15 | A | G | 0.00107723348509969 | 62.851027209663 | 0.523746 | A | G |
| rs502919 | 10 | 6.17007e-10 | C | T | 0.000656243505366125 | 38.2722999280117 | 0.214403 | C | T |
| rs2275806 | 10 | 2.51397e-09 | A | G | 0.000605541537591372 | 35.3135557187281 | 0.471244 | A | G |
| rs1538981 | 10 | 4.41601e-09 | T | C | 0.000594056723991988 | 34.6433941289253 | 0.327671 | T | C |
| rs71508903 | 10 | 3.12608e-25 | T | C | 0.00185180621703494 | 108.127200563464 | 0.747248 | T | C |
| rs9943599 | 11 | 2.70402e-10 | T | C | 0.00068828011840664 | 40.1419707813783 | 5.21003e-06 | T | C |
| rs660442 | 11 | 1.11301e-09 | A | G | 0.000637421158338194 | 37.1738753649617 | 0.527295 | A | G |
| rs34502849 | 11 | 1.068e-09 | A | G | 0.000633546456743195 | 36.9477627160603 | 0.739266 | A | G |
| rs4409785 | 11 | 7.84802e-09 | C | T | 0.000572172816140967 | 33.3664674559768 | 0.712417 | C | T |
| rs6421571 | 11 | 5.57057e-14 | C | T | 0.000971400144704648 | 56.6701926670335 | 0.764432 | C | T |
| rs7105899 | 11 | 4.63895e-10 | A | G | 0.000664750328436899 | 38.7687501813755 | 0.432849 | A | G |
| rs4622308 | 12 | 2.20699e-12 | T | C | 0.000845769827270578 | 49.3348830284812 | 0.770018 | T | C |
| rs9532434 | 13 | 1.94089e-19 | C | T | 0.00140252214664057 | 81.8566014468835 | 0.0358509 | C | T |
| rs1950897 | 14 | 1.02212e-13 | T | C | 0.000944649342905473 | 55.1081108439142 | 0.815069 | T | C |
| rs1595260 | 14 | 2.28613e-11 | T | A | 0.00077105965920406 | 44.9735763681987 | 0.119931 | T | A |
| rs2841275 | 14 | 1.7108e-19 | C | A | 0.00139816037628769 | 81.601675279814 | 0.442761 | C | A |
| rs8032939 | 15 | 4.46581e-24 | C | T | 0.00175194175045908 | 102.285867982857 | 0.147838 | C | T |
| rs7170107 | 15 | 6.11223e-18 | T | C | 0.00128079986879687 | 74.7433091738027 | 0.120499 | T | C |
| rs7206670 | 16 | 4.142e-09 | T | G | 0.000595023709630473 | 34.6998190597437 | 0.174484 | T | G |
| rs2847297 | 18 | 2.64972e-14 | G | A | 0.000986968703644098 | 57.5793389913463 | 0.137601 | G | A |
| rs34536443 | 19 | 1.07895e-15 | C | G | 0.00110207415477557 | 64.3019514073775 | 0.265877 | C | G |
| rs1355208 | 2 | 6.76862e-12 | G | A | 0.000810049190921235 | 47.2495614142664 | 0.15414 | G | A |
| rs28421442 | 2 | 7.86303e-09 | A | T | 0.000570171795089792 | 33.2497106086072 | 0.857744 | A | T |
| rs11123811 | 2 | 2.01094e-18 | C | T | 0.00130532856512629 | 76.1765949180311 | 0.383023 | C | T |
| rs11889341 | 2 | 4.32215e-30 | T | C | 0.00221094483306058 | 129.143816614502 | 0.463852 | T | C |
| rs3087243 | 2 | 3.31513e-24 | A | G | 0.00177119977751859 | 103.412229150603 | 0.303279 | A | G |
| rs1883832 | 20 | 1.13006e-16 | C | T | 0.00117588190877531 | 68.6134306991008 | 0.687257 | C | T |
| rs6011186 | 20 | 3.19396e-10 | T | C | 0.000676352589078809 | 39.4458609068439 | 0.136289 | T | C |
| rs8126756 | 21 | 1.81401e-09 | C | T | 0.00061878584168435 | 36.0864061822702 | 0.773215 | C | T |
| rs1893592 | 21 | 1.48115e-13 | C | A | 0.000937120085919884 | 54.6684637630414 | 0.351742 | C | A |
| rs7278257 | 21 | 4.27504e-10 | C | G | 0.000670135275503143 | 39.0830150339214 | 0.208156 | C | G |
| rs2073609 | 21 | 1.46501e-08 | C | T | 0.000548151364659465 | 31.9648794273622 | 0.225052 | C | T |
| rs5754104 | 22 | 1.355e-10 | A | G | 0.000704482804846108 | 41.0876123484325 | 0.908031 | A | G |
| rs2069235 | 22 | 1.69005e-20 | A | G | 0.0014681367501917 | 85.6917532868614 | 0.600968 | A | G |
| rs4602367 | 3 | 1.75699e-10 | G | A | 0.000704523323802617 | 41.0899772051797 | 0.346548 | G | A |
| rs34046593 | 4 | 7.16638e-17 | A | G | 0.00119903249995213 | 69.9659035544603 | 0.0579816 | A | G |
| rs2918392 | 5 | 4.62104e-08 | C | T | 0.000514115359899186 | 29.9790841132627 | 0.0140751 | C | T |
| rs7731626 | 5 | 1.94089e-26 | A | G | 0.00193513591072256 | 113.002265891452 | 0.492514 | A | G |
| rs403214 | 5 | 3.95804e-10 | G | A | 0.000671963065116686 | 39.1896853822411 | 0.0314543 | G | A |
| rs244685 | 5 | 6.03601e-10 | G | T | 0.00065496959057473 | 38.1979561775948 | 0.524476 | G | T |
| rs9405192 | 6 | 9.25977e-11 | A | G | 0.000723561044714213 | 42.2011198944327 | 0.334089 | A | G |
| rs12530098 | 6 | 1.35394e-11 | T | C | 0.000786800385694845 | 45.8924082435734 | 0.203179 | T | C |
| rs9258357 | 6 | 5.76501e-22 | C | T | 0.00159842263977987 | 93.3084145739876 | 3.16002e-06 | C | T |
| rs112733823 | 6 | 3.82032e-24 | T | C | 0.00176780053508964 | 103.21341151019 | 0.0254771 | T | C |
| rs41316148 | 6 | 2.474e-12 | C | T | 0.000840552377068482 | 49.0302861638887 | 0.548022 | C | T |
| rs115521560 | 6 | 1.29122e-103 | C | A | 0.0079726642579076 | 468.397191829171 | 0.0735292 | C | A |
| rs71565312 | 6 | 1.1051e-67 | A | G | 0.00516066708279514 | 302.334245307225 | 0.704086 | A | G |
| rs2233424 | 6 | 6.49232e-26 | T | C | 0.0018889882274731 | 110.302371755295 | 0.483818 | T | C |
| rs76153210 | 6 | 6.83282e-15 | T | C | 0.00104016153387336 | 60.6858175702955 | 0.0421124 | T | C |
| rs62422878 | 6 | 3.57396e-09 | T | C | 0.000595284016876986 | 34.7150083612477 | 0.0454245 | T | C |
| rs7749323 | 6 | 3.46817e-29 | A | G | 0.0021497161530944 | 125.559674495087 | 0.309208 | A | G |
| rs4717901 | 7 | 9.52138e-13 | C | A | 0.000872608333697627 | 50.9017762186936 | 0.962612 | C | A |
| rs117026326 | 7 | 2.44681e-19 | T | C | 0.00138346329791184 | 80.7427125082266 | 0.11573 | T | C |
| rs3757387 | 7 | 1.86681e-19 | C | T | 0.00139457020037203 | 81.3918470625492 | 0.0356262 | C | T |
| rs11574914 | 9 | 9.91745e-15 | A | G | 0.00102633884407141 | 59.8785361777755 | 0.763452 | A | G |
| rs10435844 | 9 | 9.72971e-11 | T | G | 0.000719779256577692 | 41.9803912466634 | 0.0118321 | T | G |

**RA and NETs**

| SNP | chr.exposure | pval.exposure | effect_allele.exposure | other_allele.exposure | r2 | F | pval.outcome | effect_allele.outcome | other_allele.outcome |
| --- | --- | --- | --- | --- | --- | --- | --- | --- | --- |
| rs2258734 | 1 | 6.04366e-14 | A | G | 0.000961041434499621 | 56.0652979598839 | 0.559515966014314 | G | A |
| rs2076616 | 1 | 6.20297e-11 | G | A | 0.000736799884008522 | 42.9738339556586 | 0.183155543916008 | A | G |
| rs2301888 | 1 | 3.75232e-26 | A | G | 0.0019222962296568 | 112.251048624403 | 0.893249012239931 | G | A |
| rs28411352 | 1 | 1.66418e-11 | T | C | 0.000774334849760245 | 45.164756358556 | 0.637263232818091 | C | T |
| rs6679677 | 1 | 1.40929e-145 | A | C | 0.0112015396813439 | 660.243883771518 | 0.836495028046296 | C | A |
| rs12126142 | 1 | 1.013e-10 | A | G | 0.00071862502862948 | 41.9130236664156 | 0.44437860707924 | G | A |
| rs3761959 | 1 | 9.64495e-11 | T | C | 0.000717610978962601 | 41.8538378494508 | 0.968124765896517 | C | T |
| rs1234313 | 1 | 1.89701e-09 | G | A | 0.000615738794392477 | 35.9085987320739 | 0.55605194290023 | A | G |
| rs61828284 | 1 | 6.32805e-09 | T | C | 0.000576612422506277 | 33.6255140976527 | 0.949342969589124 | C | T |
| rs10911902 | 1 | 2.357e-08 | T | C | 0.00053247470649769 | 31.0502243031709 | 0.947739311320541 | C | T |
| rs3134883 | 10 | 1.98381e-15 | A | G | 0.00107723348509969 | 62.851027209663 | 0.450571046738412 | G | A |
| rs502919 | 10 | 6.17007e-10 | C | T | 0.000656243505366125 | 38.2722999280117 | 0.00215898336960209 | T | C |
| rs2275806 | 10 | 2.51397e-09 | A | G | 0.000605541537591372 | 35.3135557187281 | 0.861998764981099 | G | A |
| rs1538981 | 10 | 4.41601e-09 | T | C | 0.000594056723991988 | 34.6433941289253 | 0.27358754718897 | C | T |
| rs7097397 | 10 | 1.41612e-12 | A | G | 0.000854051207089392 | 49.8183598819764 | 0.615467328932542 | G | A |
| rs71508903 | 10 | 3.12608e-25 | T | C | 0.00185180621703494 | 108.127200563464 | 0.651450395830517 | C | T |
| rs6479800 | 10 | 3.01093e-11 | C | G | 0.000756090786340865 | 44.0998266821519 | 0.146180391180986 | G | C |
| rs9943599 | 11 | 2.70402e-10 | T | C | 0.00068828011840664 | 40.1419707813783 | 0.365047128921385 | C | T |
| rs660442 | 11 | 1.11301e-09 | A | G | 0.000637421158338194 | 37.1738753649617 | 0.180774166635775 | G | A |
| rs34502849 | 11 | 1.068e-09 | A | G | 0.000633546456743195 | 36.9477627160603 | 0.00816674593875079 | G | A |
| rs4409785 | 11 | 7.84802e-09 | C | T | 0.000572172816140967 | 33.3664674559768 | 0.990754390584525 | T | C |
| rs7105899 | 11 | 4.63895e-10 | A | G | 0.000664750328436899 | 38.7687501813755 | 0.0396694343751586 | G | A |
| rs4963581 | 12 | 3.75301e-08 | A | G | 0.00051632682328896 | 30.1081055374145 | 0.65705915775293 | G | A |
| rs4622308 | 12 | 2.20699e-12 | T | C | 0.000845769827270578 | 49.3348830284812 | 0.638418581249483 | C | T |
| rs9532434 | 13 | 1.94089e-19 | C | T | 0.00140252214664057 | 81.8566014468835 | 0.470520112969995 | T | C |
| rs1950897 | 14 | 1.02212e-13 | T | C | 0.000944649342905473 | 55.1081108439142 | 0.475613178441678 | C | T |
| rs1595260 | 14 | 2.28613e-11 | T | A | 0.00077105965920406 | 44.9735763681987 | 0.140749015145068 | A | T |
| rs2841275 | 14 | 1.7108e-19 | C | A | 0.00139816037628769 | 81.601675279814 | 0.475227491815593 | A | C |
| rs8032939 | 15 | 4.46581e-24 | C | T | 0.00175194175045908 | 102.285867982857 | 0.359309753981109 | T | C |
| rs7170107 | 15 | 6.11223e-18 | T | C | 0.00128079986879687 | 74.7433091738027 | 0.392796189107831 | C | T |
| rs7206670 | 16 | 4.142e-09 | T | G | 0.000595023709630473 | 34.6998190597437 | 0.302163047432623 | G | T |
| rs12918327 | 16 | 3.04299e-08 | T | C | 0.00052295264721102 | 30.4946734549618 | 0.153245645132811 | C | T |
| rs9927316 | 16 | 2.30303e-11 | G | C | 0.000760849344255047 | 44.3775861391863 | 0.977009029534962 | C | G |
| rs4795400 | 17 | 5.85504e-10 | T | C | 0.000657325104866287 | 38.3354205961804 | 0.344062605763386 | C | T |
| rs2847297 | 18 | 2.64972e-14 | G | A | 0.000986968703644098 | 57.5793389913463 | 0.322603361706186 | A | G |
| rs34536443 | 19 | 1.07895e-15 | C | G | 0.00110207415477557 | 64.3019514073775 | 0.136183158514448 | G | C |
| rs1355208 | 2 | 6.76862e-12 | G | A | 0.000810049190921235 | 47.2495614142664 | 0.435454243830268 | A | G |
| rs12466919 | 2 | 1.58891e-11 | T | C | 0.000779601196224082 | 45.4721670741779 | 0.195939622308398 | C | T |
| rs1858037 | 2 | 1.14393e-14 | A | T | 0.00102287985551872 | 59.6765256552822 | 0.87549502039412 | T | A |
| rs28421442 | 2 | 7.86303e-09 | A | T | 0.000570171795089792 | 33.2497106086072 | 0.599652581314026 | T | A |
| rs11123811 | 2 | 2.01094e-18 | C | T | 0.00130532856512629 | 76.1765949180311 | 0.250171289158313 | T | C |
| rs11889341 | 2 | 4.32215e-30 | T | C | 0.00221094483306058 | 129.143816614502 | 0.554830830884105 | C | T |
| rs3087243 | 2 | 3.31513e-24 | A | G | 0.00177119977751859 | 103.412229150603 | 0.27912274692044 | G | A |
| rs1883832 | 20 | 1.13006e-16 | C | T | 0.00117588190877531 | 68.6134306991008 | 0.744533600942092 | T | C |
| rs6011186 | 20 | 3.19396e-10 | T | C | 0.000676352589078809 | 39.4458609068439 | 0.665984963071262 | C | T |
| rs8126756 | 21 | 1.81401e-09 | C | T | 0.00061878584168435 | 36.0864061822702 | 0.0898013789882993 | T | C |
| rs1893592 | 21 | 1.48115e-13 | C | A | 0.000937120085919884 | 54.6684637630414 | 0.746325028585779 | A | C |
| rs7278257 | 21 | 4.27504e-10 | C | G | 0.000670135275503143 | 39.0830150339214 | 0.247837004793192 | G | C |
| rs2073609 | 21 | 1.46501e-08 | C | T | 0.000548151364659465 | 31.9648794273622 | 0.460799462981118 | T | C |
| rs5754104 | 22 | 1.355e-10 | A | G | 0.000704482804846108 | 41.0876123484325 | 0.879937998850906 | G | A |
| rs2069235 | 22 | 1.69005e-20 | A | G | 0.0014681367501917 | 85.6917532868614 | 0.306933904157944 | G | A |
| rs4602367 | 3 | 1.75699e-10 | G | A | 0.000704523323802617 | 41.0899772051797 | 0.920279947093625 | A | G |
| rs3806624 | 3 | 3.93641e-11 | G | A | 0.00074405731438719 | 43.3974385787144 | 0.534015809232656 | A | G |
| rs13103285 | 4 | 4.29042e-14 | T | C | 0.00097695857072941 | 56.9947809589962 | 0.795315181658907 | C | T |
| rs34046593 | 4 | 7.16638e-17 | A | G | 0.00119903249995213 | 69.9659035544603 | 0.488149044112167 | G | A |
| rs2918392 | 5 | 4.62104e-08 | C | T | 0.000514115359899186 | 29.9790841132627 | 0.415620929852403 | T | C |
| rs7731626 | 5 | 1.94089e-26 | A | G | 0.00193513591072256 | 113.002265891452 | 0.880254730411724 | G | A |
| rs244685 | 5 | 6.03601e-10 | G | T | 0.00065496959057473 | 38.1979561775948 | 0.564391444226025 | T | G |
| rs9405192 | 6 | 9.25977e-11 | A | G | 0.000723561044714213 | 42.2011198944327 | 0.190956824709052 | G | A |
| rs12530098 | 6 | 1.35394e-11 | T | C | 0.000786800385694845 | 45.8924082435734 | 0.0849420414335235 | C | T |
| rs146305655 | 6 | 3.29079e-22 | A | G | 0.00160777348524116 | 93.8551520918116 | 0.0619514284080153 | G | A |
| rs2233424 | 6 | 6.49232e-26 | T | C | 0.0018889882274731 | 110.302371755295 | 0.07596629244604 | C | T |
| rs76153210 | 6 | 6.83282e-15 | T | C | 0.00104016153387336 | 60.6858175702955 | 0.368422947315324 | C | T |
| rs62422878 | 6 | 3.57396e-09 | T | C | 0.000595284016876986 | 34.7150083612477 | 0.829331150185781 | C | T |
| rs7749323 | 6 | 3.46817e-29 | A | G | 0.0021497161530944 | 125.559674495087 | 0.453260039281946 | G | A |
| rs212389 | 6 | 6.65733e-13 | A | G | 0.000887976559838028 | 51.7990461993269 | 0.700291412285641 | G | A |
| rs1571878 | 6 | 4.12572e-40 | T | C | 0.00301094140472042 | 176.013653747781 | 0.639137511813891 | C | T |
| rs740122 | 7 | 5.37205e-09 | A | G | 0.000583983887497936 | 34.0556368743678 | 0.294155652386993 | G | A |
| rs42034 | 7 | 1.28499e-08 | G | A | 0.000555728515629577 | 32.4069788301643 | 0.21502980754351 | A | G |
| rs3757387 | 7 | 1.86681e-19 | C | T | 0.00139457020037203 | 81.3918470625492 | 0.628616381128651 | T | C |
| rs9693589 | 8 | 1.49589e-18 | A | G | 0.00132831561999551 | 77.5198618078778 | 0.801981241543583 | G | A |
| rs11574914 | 9 | 9.91745e-15 | A | G | 0.00102633884407141 | 59.8785361777755 | 0.554231882470087 | G | A |
| rs10435844 | 9 | 9.72971e-11 | T | G | 0.000719779256577692 | 41.9803912466634 | 0.147030186769013 | G | T |

**RA and MPO-DNA**

| SNP | chr.exposure | pval.exposure | effect_allele.exposure | other_allele.exposure | r2 | F | pval.outcome | effect_allele.outcome | other_allele.outcome |
| --- | --- | --- | --- | --- | --- | --- | --- | --- | --- |
| rs2258734 | 1 | 6.04366e-14 | A | G | 0.000961041434499621 | 56.0652979598839 | 0.1172 | A | G |
| rs2076616 | 1 | 6.20297e-11 | G | A | 0.000736799884008522 | 42.9738339556586 | 0.2653 | G | A |
| rs2301888 | 1 | 3.75232e-26 | A | G | 0.0019222962296568 | 112.251048624403 | 0.3978 | A | G |
| rs28411352 | 1 | 1.66418e-11 | T | C | 0.000774334849760245 | 45.164756358556 | 0.8185 | T | C |
| rs6679677 | 1 | 1.40929e-145 | A | C | 0.0112015396813439 | 660.243883771518 | 0.0308198 | A | C |
| rs12126142 | 1 | 1.013e-10 | A | G | 0.00071862502862948 | 41.9130236664156 | 0.663001 | A | G |
| rs3761959 | 1 | 9.64495e-11 | T | C | 0.000717610978962601 | 41.8538378494508 | 0.1496 | T | C |
| rs1234313 | 1 | 1.89701e-09 | G | A | 0.000615738794392477 | 35.9085987320739 | 0.0965606 | G | A |
| rs61828284 | 1 | 6.32805e-09 | T | C | 0.000576612422506277 | 33.6255140976527 | 0.0927705 | T | C |
| rs10911902 | 1 | 2.357e-08 | T | C | 0.00053247470649769 | 31.0502243031709 | 0.2027 | T | C |
| rs3134883 | 10 | 1.98381e-15 | A | G | 0.00107723348509969 | 62.851027209663 | 0.8073 | A | G |
| rs502919 | 10 | 6.17007e-10 | C | T | 0.000656243505366125 | 38.2722999280117 | 0.6652 | C | T |
| rs2275806 | 10 | 2.51397e-09 | A | G | 0.000605541537591372 | 35.3135557187281 | 0.2446 | A | G |
| rs1538981 | 10 | 4.41601e-09 | T | C | 0.000594056723991988 | 34.6433941289253 | 0.2586 | T | C |
| rs7097397 | 10 | 1.41612e-12 | A | G | 0.000854051207089392 | 49.8183598819764 | 0.7387 | A | G |
| rs71508903 | 10 | 3.12608e-25 | T | C | 0.00185180621703494 | 108.127200563464 | 0.6977 | T | C |
| rs6479800 | 10 | 3.01093e-11 | C | G | 0.000756090786340865 | 44.0998266821519 | 0.9072 | C | G |
| rs9943599 | 11 | 2.70402e-10 | T | C | 0.00068828011840664 | 40.1419707813783 | 0.2198 | T | C |
| rs660442 | 11 | 1.11301e-09 | A | G | 0.000637421158338194 | 37.1738753649617 | 0.167 | A | G |
| rs34502849 | 11 | 1.068e-09 | A | G | 0.000633546456743195 | 36.9477627160603 | 0.4608 | A | G |
| rs4409785 | 11 | 7.84802e-09 | C | T | 0.000572172816140967 | 33.3664674559768 | 0.4247 | C | T |
| rs6421571 | 11 | 5.57057e-14 | C | T | 0.000971400144704648 | 56.6701926670335 | 0.3308 | C | T |
| rs4963581 | 12 | 3.75301e-08 | A | G | 0.00051632682328896 | 30.1081055374145 | 0.537499 | A | G |
| rs4622308 | 12 | 2.20699e-12 | T | C | 0.000845769827270578 | 49.3348830284812 | 0.1345 | T | C |
| rs9532434 | 13 | 1.94089e-19 | C | T | 0.00140252214664057 | 81.8566014468835 | 0.5012 | C | T |
| rs1950897 | 14 | 1.02212e-13 | T | C | 0.000944649342905473 | 55.1081108439142 | 0.4611 | T | C |
| rs1595260 | 14 | 2.28613e-11 | T | A | 0.00077105965920406 | 44.9735763681987 | 0.837 | T | A |
| rs2841275 | 14 | 1.7108e-19 | C | A | 0.00139816037628769 | 81.601675279814 | 0.0626297 | C | A |
| rs8032939 | 15 | 4.46581e-24 | C | T | 0.00175194175045908 | 102.285867982857 | 0.4594 | C | T |
| rs7170107 | 15 | 6.11223e-18 | T | C | 0.00128079986879687 | 74.7433091738027 | 0.146 | T | C |
| rs7206670 | 16 | 4.142e-09 | T | G | 0.000595023709630473 | 34.6998190597437 | 0.9436 | T | G |
| rs12918327 | 16 | 3.04299e-08 | T | C | 0.00052295264721102 | 30.4946734549618 | 0.9046 | T | C |
| rs9927316 | 16 | 2.30303e-11 | G | C | 0.000760849344255047 | 44.3775861391863 | 0.000970398 | G | C |
| rs4795400 | 17 | 5.85504e-10 | T | C | 0.000657325104866287 | 38.3354205961804 | 0.747799 | T | C |
| rs2847297 | 18 | 2.64972e-14 | G | A | 0.000986968703644098 | 57.5793389913463 | 0.4863 | G | A |
| rs34536443 | 19 | 1.07895e-15 | C | G | 0.00110207415477557 | 64.3019514073775 | 0.7716 | C | G |
| rs1355208 | 2 | 6.76862e-12 | G | A | 0.000810049190921235 | 47.2495614142664 | 0.3228 | G | A |
| rs12466919 | 2 | 1.58891e-11 | T | C | 0.000779601196224082 | 45.4721670741779 | 0.2627 | T | C |
| rs1858037 | 2 | 1.14393e-14 | A | T | 0.00102287985551872 | 59.6765256552822 | 0.1037 | A | T |
| rs28421442 | 2 | 7.86303e-09 | A | T | 0.000570171795089792 | 33.2497106086072 | 0.6467 | A | T |
| rs11123811 | 2 | 2.01094e-18 | C | T | 0.00130532856512629 | 76.1765949180311 | 0.1249 | C | T |
| rs11889341 | 2 | 4.32215e-30 | T | C | 0.00221094483306058 | 129.143816614502 | 0.5572 | T | C |
| rs3087243 | 2 | 3.31513e-24 | A | G | 0.00177119977751859 | 103.412229150603 | 0.0117899 | A | G |
| rs1883832 | 20 | 1.13006e-16 | C | T | 0.00117588190877531 | 68.6134306991008 | 0.4832 | C | T |
| rs6011186 | 20 | 3.19396e-10 | T | C | 0.000676352589078809 | 39.4458609068439 | 0.2535 | T | C |
| rs8126756 | 21 | 1.81401e-09 | C | T | 0.00061878584168435 | 36.0864061822702 | 0.1656 | C | T |
| rs1893592 | 21 | 1.48115e-13 | C | A | 0.000937120085919884 | 54.6684637630414 | 0.7719 | C | A |
| rs7278257 | 21 | 4.27504e-10 | C | G | 0.000670135275503143 | 39.0830150339214 | 0.6407 | C | G |
| rs2073609 | 21 | 1.46501e-08 | C | T | 0.000548151364659465 | 31.9648794273622 | 0.2394 | C | T |
| rs5754104 | 22 | 1.355e-10 | A | G | 0.000704482804846108 | 41.0876123484325 | 0.4287 | A | G |
| rs2069235 | 22 | 1.69005e-20 | A | G | 0.0014681367501917 | 85.6917532868614 | 0.9819 | A | G |
| rs4602367 | 3 | 1.75699e-10 | G | A | 0.000704523323802617 | 41.0899772051797 | 0.8887 | G | A |
| rs3806624 | 3 | 3.93641e-11 | G | A | 0.00074405731438719 | 43.3974385787144 | 0.9573 | G | A |
| rs13103285 | 4 | 4.29042e-14 | T | C | 0.00097695857072941 | 56.9947809589962 | 0.0578402 | T | C |
| rs34046593 | 4 | 7.16638e-17 | A | G | 0.00119903249995213 | 69.9659035544603 | 0.6055 | A | G |
| rs2918392 | 5 | 4.62104e-08 | C | T | 0.000514115359899186 | 29.9790841132627 | 0.9378 | C | T |
| rs7731626 | 5 | 1.94089e-26 | A | G | 0.00193513591072256 | 113.002265891452 | 0.2789 | A | G |
| rs403214 | 5 | 3.95804e-10 | G | A | 0.000671963065116686 | 39.1896853822411 | 0.2938 | G | A |
| rs244685 | 5 | 6.03601e-10 | G | T | 0.00065496959057473 | 38.1979561775948 | 0.4911 | G | T |
| rs9405192 | 6 | 9.25977e-11 | A | G | 0.000723561044714213 | 42.2011198944327 | 0.1402 | A | G |
| rs12530098 | 6 | 1.35394e-11 | T | C | 0.000786800385694845 | 45.8924082435734 | 0.508899 | T | C |
| rs1611236 | 6 | 4.5436e-19 | A | G | 0.00135509891890628 | 79.0850432483033 | 0.7633 | A | G |
| rs112733823 | 6 | 3.82032e-24 | T | C | 0.00176780053508964 | 103.21341151019 | 0.6999 | T | C |
| rs146305655 | 6 | 3.29079e-22 | A | G | 0.00160777348524116 | 93.8551520918116 | 0.8674 | A | G |
| rs2233424 | 6 | 6.49232e-26 | T | C | 0.0018889882274731 | 110.302371755295 | 0.8898 | T | C |
| rs76153210 | 6 | 6.83282e-15 | T | C | 0.00104016153387336 | 60.6858175702955 | 0.3506 | T | C |
| rs62422878 | 6 | 3.57396e-09 | T | C | 0.000595284016876986 | 34.7150083612477 | 0.5261 | T | C |
| rs7749323 | 6 | 3.46817e-29 | A | G | 0.0021497161530944 | 125.559674495087 | 0.609901 | A | G |
| rs212389 | 6 | 6.65733e-13 | A | G | 0.000887976559838028 | 51.7990461993269 | 0.8023 | A | G |
| rs1571878 | 6 | 4.12572e-40 | T | C | 0.00301094140472042 | 176.013653747781 | 0.5405 | T | C |
| rs740122 | 7 | 5.37205e-09 | A | G | 0.000583983887497936 | 34.0556368743678 | 0.481599 | A | G |
| rs42034 | 7 | 1.28499e-08 | G | A | 0.000555728515629577 | 32.4069788301643 | 0.1578 | G | A |
| rs3757387 | 7 | 1.86681e-19 | C | T | 0.00139457020037203 | 81.3918470625492 | 0.8508 | C | T |
| rs9693589 | 8 | 1.49589e-18 | A | G | 0.00132831561999551 | 77.5198618078778 | 0.1392 | A | G |
| rs11574914 | 9 | 9.91745e-15 | A | G | 0.00102633884407141 | 59.8785361777755 | 0.1663 | A | G |
| rs10435844 | 9 | 9.72971e-11 | T | G | 0.000719779256577692 | 41.9803912466634 | 0.750601 | T | G |

**RA and MPO**

| SNP | chr.exposure | pval.exposure | effect_allele.exposure | other_allele.exposure | r2 | F | pval.outcome | effect_allele.outcome | other_allele.outcome |
| --- | --- | --- | --- | --- | --- | --- | --- | --- | --- |
| rs2076616 | 1 | 6.20297e-11 | G | A | 0.000736799884008522 | 42.9738339556586 | 0.552561981002645 | G | A |
| rs2301888 | 1 | 3.75232e-26 | A | G | 0.0019222962296568 | 112.251048624403 | 0.992622099506925 | A | G |
| rs28411352 | 1 | 1.66418e-11 | T | C | 0.000774334849760245 | 45.164756358556 | 0.0179626331330432 | T | C |
| rs6679677 | 1 | 1.40929e-145 | A | C | 0.0112015396813439 | 660.243883771518 | 0.0147112643673781 | A | C |
| rs12126142 | 1 | 1.013e-10 | A | G | 0.00071862502862948 | 41.9130236664156 | 0.961404388032996 | A | G |
| rs3761959 | 1 | 9.64495e-11 | T | C | 0.000717610978962601 | 41.8538378494508 | 0.0504463791873079 | T | C |
| rs1234313 | 1 | 1.89701e-09 | G | A | 0.000615738794392477 | 35.9085987320739 | 0.565039748580588 | A | G |
| rs61828284 | 1 | 6.32805e-09 | T | C | 0.000576612422506277 | 33.6255140976527 | 0.861975456309959 | T | C |
| rs10911902 | 1 | 2.357e-08 | T | C | 0.00053247470649769 | 31.0502243031709 | 0.97196091231247 | T | C |
| rs3134883 | 10 | 1.98381e-15 | A | G | 0.00107723348509969 | 62.851027209663 | 0.868380174975509 | A | G |
| rs502919 | 10 | 6.17007e-10 | C | T | 0.000656243505366125 | 38.2722999280117 | 0.986964632109409 | C | T |
| rs2275806 | 10 | 2.51397e-09 | A | G | 0.000605541537591372 | 35.3135557187281 | 0.425403442470565 | G | A |
| rs1538981 | 10 | 4.41601e-09 | T | C | 0.000594056723991988 | 34.6433941289253 | 0.198972932177432 | C | T |
| rs7097397 | 10 | 1.41612e-12 | A | G | 0.000854051207089392 | 49.8183598819764 | 0.0703218034527604 | A | G |
| rs71508903 | 10 | 3.12608e-25 | T | C | 0.00185180621703494 | 108.127200563464 | 0.946384023314202 | T | C |
| rs6479800 | 10 | 3.01093e-11 | C | G | 0.000756090786340865 | 44.0998266821519 | 0.344822820602452 | C | G |
| rs9943599 | 11 | 2.70402e-10 | T | C | 0.00068828011840664 | 40.1419707813783 | 0.931171338701897 | C | T |
| rs660442 | 11 | 1.11301e-09 | A | G | 0.000637421158338194 | 37.1738753649617 | 0.447897872948378 | A | G |
| rs34502849 | 11 | 1.068e-09 | A | G | 0.000633546456743195 | 36.9477627160603 | 0.849277072859072 | A | G |
| rs4409785 | 11 | 7.84802e-09 | C | T | 0.000572172816140967 | 33.3664674559768 | 0.758963691623647 | C | T |
| rs6421571 | 11 | 5.57057e-14 | C | T | 0.000971400144704648 | 56.6701926670335 | 0.4658820738039 | T | C |
| rs4963581 | 12 | 3.75301e-08 | A | G | 0.00051632682328896 | 30.1081055374145 | 0.291221879224086 | A | G |
| rs9532434 | 13 | 1.94089e-19 | C | T | 0.00140252214664057 | 81.8566014468835 | 0.594559058142314 | T | C |
| rs1950897 | 14 | 1.02212e-13 | T | C | 0.000944649342905473 | 55.1081108439142 | 0.882851786152336 | C | T |
| rs1595260 | 14 | 2.28613e-11 | T | A | 0.00077105965920406 | 44.9735763681987 | 0.674638310943302 | A | T |
| rs8032939 | 15 | 4.46581e-24 | C | T | 0.00175194175045908 | 102.285867982857 | 0.715538489763854 | C | T |
| rs7170107 | 15 | 6.11223e-18 | T | C | 0.00128079986879687 | 74.7433091738027 | 0.028076954616225 | T | C |
| rs7206670 | 16 | 4.142e-09 | T | G | 0.000595023709630473 | 34.6998190597437 | 0.00931043559035846 | G | T |
| rs12918327 | 16 | 3.04299e-08 | T | C | 0.00052295264721102 | 30.4946734549618 | 0.114606434108652 | T | C |
| rs9927316 | 16 | 2.30303e-11 | G | C | 0.000760849344255047 | 44.3775861391863 | 0.00749031358510757 | G | C |
| rs4795400 | 17 | 5.85504e-10 | T | C | 0.000657325104866287 | 38.3354205961804 | 0.000620354590116912 | T | C |
| rs2847297 | 18 | 2.64972e-14 | G | A | 0.000986968703644098 | 57.5793389913463 | 0.50099224113237 | G | A |
| rs34536443 | 19 | 1.07895e-15 | C | G | 0.00110207415477557 | 64.3019514073775 | 0.472354437869952 | C | G |
| rs1355208 | 2 | 6.76862e-12 | G | A | 0.000810049190921235 | 47.2495614142664 | 0.054875690752006 | A | G |
| rs12466919 | 2 | 1.58891e-11 | T | C | 0.000779601196224082 | 45.4721670741779 | 0.654422164575816 | T | C |
| rs1858037 | 2 | 1.14393e-14 | A | T | 0.00102287985551872 | 59.6765256552822 | 0.278839952446702 | A | T |
| rs28421442 | 2 | 7.86303e-09 | A | T | 0.000570171795089792 | 33.2497106086072 | 0.660781689467082 | A | T |
| rs11123811 | 2 | 2.01094e-18 | C | T | 0.00130532856512629 | 76.1765949180311 | 0.411200846472726 | T | C |
| rs11889341 | 2 | 4.32215e-30 | T | C | 0.00221094483306058 | 129.143816614502 | 0.103379398273548 | T | C |
| rs3087243 | 2 | 3.31513e-24 | A | G | 0.00177119977751859 | 103.412229150603 | 0.521432384124059 | A | G |
| rs1883832 | 20 | 1.13006e-16 | C | T | 0.00117588190877531 | 68.6134306991008 | 0.0537044162126706 | T | C |
| rs6011186 | 20 | 3.19396e-10 | T | C | 0.000676352589078809 | 39.4458609068439 | 0.136623686603478 | T | C |
| rs1893592 | 21 | 1.48115e-13 | C | A | 0.000937120085919884 | 54.6684637630414 | 0.0361443151251074 | C | A |
| rs7278257 | 21 | 4.27504e-10 | C | G | 0.000670135275503143 | 39.0830150339214 | 0.445050199447568 | C | G |
| rs2073609 | 21 | 1.46501e-08 | C | T | 0.000548151364659465 | 31.9648794273622 | 0.8865474806818 | C | T |
| rs2069235 | 22 | 1.69005e-20 | A | G | 0.0014681367501917 | 85.6917532868614 | 0.906660509471005 | A | G |
| rs4602367 | 3 | 1.75699e-10 | G | A | 0.000704523323802617 | 41.0899772051797 | 0.901535432071709 | A | G |
| rs3806624 | 3 | 3.93641e-11 | G | A | 0.00074405731438719 | 43.3974385787144 | 0.291032841793955 | G | A |
| rs13103285 | 4 | 4.29042e-14 | T | C | 0.00097695857072941 | 56.9947809589962 | 0.247494755206011 | T | C |
| rs34046593 | 4 | 7.16638e-17 | A | G | 0.00119903249995213 | 69.9659035544603 | 0.711832807125513 | A | G |
| rs2918392 | 5 | 4.62104e-08 | C | T | 0.000514115359899186 | 29.9790841132627 | 0.529753701622121 | C | T |
| rs7731626 | 5 | 1.94089e-26 | A | G | 0.00193513591072256 | 113.002265891452 | 0.657296016084214 | A | G |
| rs403214 | 5 | 3.95804e-10 | G | A | 0.000671963065116686 | 39.1896853822411 | 0.881889151110923 | G | A |
| rs244685 | 5 | 6.03601e-10 | G | T | 0.00065496959057473 | 38.1979561775948 | 0.477362171030143 | T | G |
| rs9405192 | 6 | 9.25977e-11 | A | G | 0.000723561044714213 | 42.2011198944327 | 0.679536830164967 | A | G |
| rs12530098 | 6 | 1.35394e-11 | T | C | 0.000786800385694845 | 45.8924082435734 | 0.894526889495318 | T | C |
| rs139395255 | 6 | 8.6836e-60 | G | A | 0.00454375326935948 | 266.027792697619 | 0.763927246491427 | G | A |
| rs114508013 | 6 | 1.81593e-28 | A | G | 0.00209653273183503 | 122.446834473192 | 0.893659136873125 | A | G |
| rs115521560 | 6 | 1.29122e-103 | C | A | 0.0079726642579076 | 468.397191829171 | 0.794607756370367 | C | A |
| rs71565312 | 6 | 1.1051e-67 | A | G | 0.00516066708279514 | 302.334245307225 | 0.40976041408257 | A | G |
| rs146305655 | 6 | 3.29079e-22 | A | G | 0.00160777348524116 | 93.8551520918116 | 0.780173150361942 | A | G |
| rs5020946 | 6 | 1e-200 | T | G | 0.0248937958134512 | 1487.89967838416 | 0.46259400476616 | T | G |
| rs9271365 | 6 | 1e-200 | G | T | 0.0244095978658332 | 1458.23511558168 | 0.632478839300475 | G | T |
| rs2233424 | 6 | 6.49232e-26 | T | C | 0.0018889882274731 | 110.302371755295 | 0.145305147618011 | T | C |
| rs76153210 | 6 | 6.83282e-15 | T | C | 0.00104016153387336 | 60.6858175702955 | 0.810605227257483 | T | C |
| rs62422878 | 6 | 3.57396e-09 | T | C | 0.000595284016876986 | 34.7150083612477 | 0.518292590238513 | T | C |
| rs7749323 | 6 | 3.46817e-29 | A | G | 0.0021497161530944 | 125.559674495087 | 0.972152953630177 | A | G |
| rs212389 | 6 | 6.65733e-13 | A | G | 0.000887976559838028 | 51.7990461993269 | 0.721963098228555 | G | A |
| rs1571878 | 6 | 4.12572e-40 | T | C | 0.00301094140472042 | 176.013653747781 | 0.352244321001723 | C | T |
| rs740122 | 7 | 5.37205e-09 | A | G | 0.000583983887497936 | 34.0556368743678 | 0.764298487247311 | A | G |
| rs42034 | 7 | 1.28499e-08 | G | A | 0.000555728515629577 | 32.4069788301643 | 0.263818957584938 | G | A |
| rs3757387 | 7 | 1.86681e-19 | C | T | 0.00139457020037203 | 81.3918470625492 | 0.544604217172492 | C | T |
| rs9693589 | 8 | 1.49589e-18 | A | G | 0.00132831561999551 | 77.5198618078778 | 0.491147570368399 | A | G |
| rs11574914 | 9 | 9.91745e-15 | A | G | 0.00102633884407141 | 59.8785361777755 | 0.559056884555116 | A | G |
| rs10435844 | 9 | 9.72971e-11 | T | G | 0.000719779256577692 | 41.9803912466634 | 0.496635774302246 | G | T |
| rs5912815 | X | 4.79104e-09 | G | T | 0.000591471155142243 | 34.492523196539 | 0.23427610771121 | T | G |

**RA and IL-18**

| SNP | chr.exposure | pval.exposure | effect_allele.exposure | other_allele.exposure | r2 | F | pval.outcome | effect_allele.outcome | other_allele.outcome |
| --- | --- | --- | --- | --- | --- | --- | --- | --- | --- |
| rs2258734 | 1 | 6.04366e-14 | A | G | 0.000961041434499621 | 56.0652979598839 | 0.580801 | A | G |
| rs2076616 | 1 | 6.20297e-11 | G | A | 0.000736799884008522 | 42.9738339556586 | 0.9315 | G | A |
| rs2301888 | 1 | 3.75232e-26 | A | G | 0.0019222962296568 | 112.251048624403 | 0.7591 | A | G |
| rs28411352 | 1 | 1.66418e-11 | T | C | 0.000774334849760245 | 45.164756358556 | 0.9667 | T | C |
| rs6679677 | 1 | 1.40929e-145 | A | C | 0.0112015396813439 | 660.243883771518 | 0.3264 | A | C |
| rs12126142 | 1 | 1.013e-10 | A | G | 0.00071862502862948 | 41.9130236664156 | 0.1093 | A | G |
| rs3761959 | 1 | 9.64495e-11 | T | C | 0.000717610978962601 | 41.8538378494508 | 0.8099 | T | C |
| rs1234313 | 1 | 1.89701e-09 | G | A | 0.000615738794392477 | 35.9085987320739 | 0.3283 | G | A |
| rs61828284 | 1 | 6.32805e-09 | T | C | 0.000576612422506277 | 33.6255140976527 | 0.0790606 | T | C |
| rs10911902 | 1 | 2.357e-08 | T | C | 0.00053247470649769 | 31.0502243031709 | 0.4853 | T | C |
| rs3134883 | 10 | 1.98381e-15 | A | G | 0.00107723348509969 | 62.851027209663 | 0.4746 | A | G |
| rs502919 | 10 | 6.17007e-10 | C | T | 0.000656243505366125 | 38.2722999280117 | 0.5569 | C | T |
| rs2275806 | 10 | 2.51397e-09 | A | G | 0.000605541537591372 | 35.3135557187281 | 0.03684 | A | G |
| rs1538981 | 10 | 4.41601e-09 | T | C | 0.000594056723991988 | 34.6433941289253 | 0.8803 | T | C |
| rs7097397 | 10 | 1.41612e-12 | A | G | 0.000854051207089392 | 49.8183598819764 | 0.8121 | A | G |
| rs71508903 | 10 | 3.12608e-25 | T | C | 0.00185180621703494 | 108.127200563464 | 0.3537 | T | C |
| rs6479800 | 10 | 3.01093e-11 | C | G | 0.000756090786340865 | 44.0998266821519 | 0.2833 | C | G |
| rs9943599 | 11 | 2.70402e-10 | T | C | 0.00068828011840664 | 40.1419707813783 | 0.6467 | T | C |
| rs660442 | 11 | 1.11301e-09 | A | G | 0.000637421158338194 | 37.1738753649617 | 0.3063 | A | G |
| rs34502849 | 11 | 1.068e-09 | A | G | 0.000633546456743195 | 36.9477627160603 | 0.4457 | A | G |
| rs4409785 | 11 | 7.84802e-09 | C | T | 0.000572172816140967 | 33.3664674559768 | 0.4068 | C | T |
| rs6421571 | 11 | 5.57057e-14 | C | T | 0.000971400144704648 | 56.6701926670335 | 0.9839 | C | T |
| rs7105899 | 11 | 4.63895e-10 | A | G | 0.000664750328436899 | 38.7687501813755 | 0.8717 | A | G |
| rs4963581 | 12 | 3.75301e-08 | A | G | 0.00051632682328896 | 30.1081055374145 | 0.9786 | A | G |
| rs4622308 | 12 | 2.20699e-12 | T | C | 0.000845769827270578 | 49.3348830284812 | 0.2776 | T | C |
| rs9532434 | 13 | 1.94089e-19 | C | T | 0.00140252214664057 | 81.8566014468835 | 0.4418 | C | T |
| rs1950897 | 14 | 1.02212e-13 | T | C | 0.000944649342905473 | 55.1081108439142 | 0.8546 | T | C |
| rs1595260 | 14 | 2.28613e-11 | T | A | 0.00077105965920406 | 44.9735763681987 | 0.9598 | T | A |
| rs2841275 | 14 | 1.7108e-19 | C | A | 0.00139816037628769 | 81.601675279814 | 0.535601 | C | A |
| rs8032939 | 15 | 4.46581e-24 | C | T | 0.00175194175045908 | 102.285867982857 | 0.5075 | C | T |
| rs7170107 | 15 | 6.11223e-18 | T | C | 0.00128079986879687 | 74.7433091738027 | 0.9503 | T | C |
| rs7206670 | 16 | 4.142e-09 | T | G | 0.000595023709630473 | 34.6998190597437 | 0.4187 | T | G |
| rs12918327 | 16 | 3.04299e-08 | T | C | 0.00052295264721102 | 30.4946734549618 | 0.617 | T | C |
| rs9927316 | 16 | 2.30303e-11 | G | C | 0.000760849344255047 | 44.3775861391863 | 0.7083 | G | C |
| rs4795400 | 17 | 5.85504e-10 | T | C | 0.000657325104866287 | 38.3354205961804 | 0.785301 | T | C |
| rs2847297 | 18 | 2.64972e-14 | G | A | 0.000986968703644098 | 57.5793389913463 | 0.6631 | G | A |
| rs34536443 | 19 | 1.07895e-15 | C | G | 0.00110207415477557 | 64.3019514073775 | 0.000645402 | C | G |
| rs1355208 | 2 | 6.76862e-12 | G | A | 0.000810049190921235 | 47.2495614142664 | 0.657201 | G | A |
| rs12466919 | 2 | 1.58891e-11 | T | C | 0.000779601196224082 | 45.4721670741779 | 0.2534 | T | C |
| rs1858037 | 2 | 1.14393e-14 | A | T | 0.00102287985551872 | 59.6765256552822 | 0.5526 | A | T |
| rs28421442 | 2 | 7.86303e-09 | A | T | 0.000570171795089792 | 33.2497106086072 | 0.4392 | A | T |
| rs11123811 | 2 | 2.01094e-18 | C | T | 0.00130532856512629 | 76.1765949180311 | 0.1355 | C | T |
| rs11889341 | 2 | 4.32215e-30 | T | C | 0.00221094483306058 | 129.143816614502 | 0.03983 | T | C |
| rs3087243 | 2 | 3.31513e-24 | A | G | 0.00177119977751859 | 103.412229150603 | 0.6306 | A | G |
| rs1883832 | 20 | 1.13006e-16 | C | T | 0.00117588190877531 | 68.6134306991008 | 0.0844909 | C | T |
| rs6011186 | 20 | 3.19396e-10 | T | C | 0.000676352589078809 | 39.4458609068439 | 0.555 | T | C |
| rs8126756 | 21 | 1.81401e-09 | C | T | 0.00061878584168435 | 36.0864061822702 | 0.869 | C | T |
| rs1893592 | 21 | 1.48115e-13 | C | A | 0.000937120085919884 | 54.6684637630414 | 0.756 | C | A |
| rs7278257 | 21 | 4.27504e-10 | C | G | 0.000670135275503143 | 39.0830150339214 | 0.5693 | C | G |
| rs2073609 | 21 | 1.46501e-08 | C | T | 0.000548151364659465 | 31.9648794273622 | 0.5967 | C | T |
| rs5754104 | 22 | 1.355e-10 | A | G | 0.000704482804846108 | 41.0876123484325 | 0.8686 | A | G |
| rs2069235 | 22 | 1.69005e-20 | A | G | 0.0014681367501917 | 85.6917532868614 | 0.9813 | A | G |
| rs4602367 | 3 | 1.75699e-10 | G | A | 0.000704523323802617 | 41.0899772051797 | 0.938 | G | A |
| rs3806624 | 3 | 3.93641e-11 | G | A | 0.00074405731438719 | 43.3974385787144 | 0.515101 | G | A |
| rs13103285 | 4 | 4.29042e-14 | T | C | 0.00097695857072941 | 56.9947809589962 | 0.6799 | T | C |
| rs34046593 | 4 | 7.16638e-17 | A | G | 0.00119903249995213 | 69.9659035544603 | 0.4008 | A | G |
| rs2918392 | 5 | 4.62104e-08 | C | T | 0.000514115359899186 | 29.9790841132627 | 0.1434 | C | T |
| rs7731626 | 5 | 1.94089e-26 | A | G | 0.00193513591072256 | 113.002265891452 | 0.4444 | A | G |
| rs403214 | 5 | 3.95804e-10 | G | A | 0.000671963065116686 | 39.1896853822411 | 0.673101 | G | A |
| rs244685 | 5 | 6.03601e-10 | G | T | 0.00065496959057473 | 38.1979561775948 | 0.4777 | G | T |
| rs9405192 | 6 | 9.25977e-11 | A | G | 0.000723561044714213 | 42.2011198944327 | 0.8752 | A | G |
| rs12530098 | 6 | 1.35394e-11 | T | C | 0.000786800385694845 | 45.8924082435734 | 0.0385398 | T | C |
| rs3025669 | 6 | 2.91273e-30 | G | C | 0.00223042784521827 | 130.284385596443 | 0.2817 | G | C |
| rs1611236 | 6 | 4.5436e-19 | A | G | 0.00135509891890628 | 79.0850432483033 | 0.7907 | A | G |
| rs9258357 | 6 | 5.76501e-22 | C | T | 0.00159842263977987 | 93.3084145739876 | 0.1595 | C | T |
| rs11754264 | 6 | 1.88105e-12 | C | T | 0.000849974017077673 | 49.5803276535847 | 0.335 | C | T |
| rs112733823 | 6 | 3.82032e-24 | T | C | 0.00176780053508964 | 103.21341151019 | 0.0648201 | T | C |
| rs139395255 | 6 | 8.6836e-60 | G | A | 0.00454375326935948 | 266.027792697619 | 0.9647 | G | A |
| rs114508013 | 6 | 1.81593e-28 | A | G | 0.00209653273183503 | 122.446834473192 | 0.0156599 | A | G |
| rs115521560 | 6 | 1.29122e-103 | C | A | 0.0079726642579076 | 468.397191829171 | 0.693999 | C | A |
| rs71565312 | 6 | 1.1051e-67 | A | G | 0.00516066708279514 | 302.334245307225 | 0.4919 | A | G |
| rs146305655 | 6 | 3.29079e-22 | A | G | 0.00160777348524116 | 93.8551520918116 | 0.2779 | A | G |
| rs5020946 | 6 | 1e-200 | T | G | 0.0248937958134512 | 1487.89967838416 | 0.604301 | T | G |
| rs9271365 | 6 | 1e-200 | G | T | 0.0244095978658332 | 1458.23511558168 | 0.5664 | G | T |
| rs2233424 | 6 | 6.49232e-26 | T | C | 0.0018889882274731 | 110.302371755295 | 0.0339899 | T | C |
| rs76153210 | 6 | 6.83282e-15 | T | C | 0.00104016153387336 | 60.6858175702955 | 0.0416303 | T | C |
| rs62422878 | 6 | 3.57396e-09 | T | C | 0.000595284016876986 | 34.7150083612477 | 0.5229 | T | C |
| rs7749323 | 6 | 3.46817e-29 | A | G | 0.0021497161530944 | 125.559674495087 | 0.8973 | A | G |
| rs212389 | 6 | 6.65733e-13 | A | G | 0.000887976559838028 | 51.7990461993269 | 0.4069 | A | G |
| rs1571878 | 6 | 4.12572e-40 | T | C | 0.00301094140472042 | 176.013653747781 | 0.0786394 | T | C |
| rs740122 | 7 | 5.37205e-09 | A | G | 0.000583983887497936 | 34.0556368743678 | 0.0655602 | A | G |
| rs42034 | 7 | 1.28499e-08 | G | A | 0.000555728515629577 | 32.4069788301643 | 0.2792 | G | A |
| rs3757387 | 7 | 1.86681e-19 | C | T | 0.00139457020037203 | 81.3918470625492 | 0.391 | C | T |
| rs9693589 | 8 | 1.49589e-18 | A | G | 0.00132831561999551 | 77.5198618078778 | 0.3574 | A | G |
| rs11574914 | 9 | 9.91745e-15 | A | G | 0.00102633884407141 | 59.8785361777755 | 0.8478 | A | G |
| rs10435844 | 9 | 9.72971e-11 | T | G | 0.000719779256577692 | 41.9803912466634 | 0.537901 | T | G |

**RA and IL-13**

| SNP | chr.exposure | pval.exposure | effect_allele.exposure | other_allele.exposure | r2 | F | pval.outcome | effect_allele.outcome | other_allele.outcome |
| --- | --- | --- | --- | --- | --- | --- | --- | --- | --- |
| rs2258734 | 1 | 6.04366e-14 | A | G | 0.000961041434499621 | 56.0652979598839 | 0.5389 | A | G |
| rs2076616 | 1 | 6.20297e-11 | G | A | 0.000736799884008522 | 42.9738339556586 | 0.1028 | G | A |
| rs2301888 | 1 | 3.75232e-26 | A | G | 0.0019222962296568 | 112.251048624403 | 0.5571 | A | G |
| rs28411352 | 1 | 1.66418e-11 | T | C | 0.000774334849760245 | 45.164756358556 | 0.8232 | T | C |
| rs6679677 | 1 | 1.40929e-145 | A | C | 0.0112015396813439 | 660.243883771518 | 0.3174 | A | C |
| rs12126142 | 1 | 1.013e-10 | A | G | 0.00071862502862948 | 41.9130236664156 | 0.0699407 | A | G |
| rs3761959 | 1 | 9.64495e-11 | T | C | 0.000717610978962601 | 41.8538378494508 | 0.1895 | T | C |
| rs1234313 | 1 | 1.89701e-09 | G | A | 0.000615738794392477 | 35.9085987320739 | 0.3098 | G | A |
| rs61828284 | 1 | 6.32805e-09 | T | C | 0.000576612422506277 | 33.6255140976527 | 0.9301 | T | C |
| rs10911902 | 1 | 2.357e-08 | T | C | 0.00053247470649769 | 31.0502243031709 | 0.119 | T | C |
| rs3134883 | 10 | 1.98381e-15 | A | G | 0.00107723348509969 | 62.851027209663 | 0.0796691 | A | G |
| rs502919 | 10 | 6.17007e-10 | C | T | 0.000656243505366125 | 38.2722999280117 | 0.8076 | C | T |
| rs2275806 | 10 | 2.51397e-09 | A | G | 0.000605541537591372 | 35.3135557187281 | 0.5539 | A | G |
| rs1538981 | 10 | 4.41601e-09 | T | C | 0.000594056723991988 | 34.6433941289253 | 0.9763 | T | C |
| rs7097397 | 10 | 1.41612e-12 | A | G | 0.000854051207089392 | 49.8183598819764 | 0.0615503 | A | G |
| rs71508903 | 10 | 3.12608e-25 | T | C | 0.00185180621703494 | 108.127200563464 | 0.3251 | T | C |
| rs6479800 | 10 | 3.01093e-11 | C | G | 0.000756090786340865 | 44.0998266821519 | 0.8494 | C | G |
| rs9943599 | 11 | 2.70402e-10 | T | C | 0.00068828011840664 | 40.1419707813783 | 0.6131 | T | C |
| rs660442 | 11 | 1.11301e-09 | A | G | 0.000637421158338194 | 37.1738753649617 | 0.3052 | A | G |
| rs34502849 | 11 | 1.068e-09 | A | G | 0.000633546456743195 | 36.9477627160603 | 0.5398 | A | G |
| rs4409785 | 11 | 7.84802e-09 | C | T | 0.000572172816140967 | 33.3664674559768 | 0.7957 | C | T |
| rs6421571 | 11 | 5.57057e-14 | C | T | 0.000971400144704648 | 56.6701926670335 | 0.0674808 | C | T |
| rs7105899 | 11 | 4.63895e-10 | A | G | 0.000664750328436899 | 38.7687501813755 | 0.5993 | A | G |
| rs4963581 | 12 | 3.75301e-08 | A | G | 0.00051632682328896 | 30.1081055374145 | 0.2628 | A | G |
| rs4622308 | 12 | 2.20699e-12 | T | C | 0.000845769827270578 | 49.3348830284812 | 0.823 | T | C |
| rs9532434 | 13 | 1.94089e-19 | C | T | 0.00140252214664057 | 81.8566014468835 | 0.5926 | C | T |
| rs1950897 | 14 | 1.02212e-13 | T | C | 0.000944649342905473 | 55.1081108439142 | 0.3909 | T | C |
| rs1595260 | 14 | 2.28613e-11 | T | A | 0.00077105965920406 | 44.9735763681987 | 0.8045 | T | A |
| rs2841275 | 14 | 1.7108e-19 | C | A | 0.00139816037628769 | 81.601675279814 | 0.144 | C | A |
| rs8032939 | 15 | 4.46581e-24 | C | T | 0.00175194175045908 | 102.285867982857 | 0.0614497 | C | T |
| rs7170107 | 15 | 6.11223e-18 | T | C | 0.00128079986879687 | 74.7433091738027 | 0.3554 | T | C |
| rs7206670 | 16 | 4.142e-09 | T | G | 0.000595023709630473 | 34.6998190597437 | 0.53 | T | G |
| rs12918327 | 16 | 3.04299e-08 | T | C | 0.00052295264721102 | 30.4946734549618 | 0.1267 | T | C |
| rs9927316 | 16 | 2.30303e-11 | G | C | 0.000760849344255047 | 44.3775861391863 | 0.7119 | G | C |
| rs4795400 | 17 | 5.85504e-10 | T | C | 0.000657325104866287 | 38.3354205961804 | 0.3589 | T | C |
| rs2847297 | 18 | 2.64972e-14 | G | A | 0.000986968703644098 | 57.5793389913463 | 0.759799 | G | A |
| rs34536443 | 19 | 1.07895e-15 | C | G | 0.00110207415477557 | 64.3019514073775 | 0.8395 | C | G |
| rs1355208 | 2 | 6.76862e-12 | G | A | 0.000810049190921235 | 47.2495614142664 | 0.281 | G | A |
| rs12466919 | 2 | 1.58891e-11 | T | C | 0.000779601196224082 | 45.4721670741779 | 0.4315 | T | C |
| rs1858037 | 2 | 1.14393e-14 | A | T | 0.00102287985551872 | 59.6765256552822 | 0.9246 | A | T |
| rs28421442 | 2 | 7.86303e-09 | A | T | 0.000570171795089792 | 33.2497106086072 | 0.2393 | A | T |
| rs11123811 | 2 | 2.01094e-18 | C | T | 0.00130532856512629 | 76.1765949180311 | 0.4805 | C | T |
| rs11889341 | 2 | 4.32215e-30 | T | C | 0.00221094483306058 | 129.143816614502 | 0.6988 | T | C |
| rs3087243 | 2 | 3.31513e-24 | A | G | 0.00177119977751859 | 103.412229150603 | 0.51 | A | G |
| rs1883832 | 20 | 1.13006e-16 | C | T | 0.00117588190877531 | 68.6134306991008 | 0.4249 | C | T |
| rs6011186 | 20 | 3.19396e-10 | T | C | 0.000676352589078809 | 39.4458609068439 | 0.4523 | T | C |
| rs8126756 | 21 | 1.81401e-09 | C | T | 0.00061878584168435 | 36.0864061822702 | 0.2016 | C | T |
| rs1893592 | 21 | 1.48115e-13 | C | A | 0.000937120085919884 | 54.6684637630414 | 0.9702 | C | A |
| rs7278257 | 21 | 4.27504e-10 | C | G | 0.000670135275503143 | 39.0830150339214 | 0.2805 | C | G |
| rs2073609 | 21 | 1.46501e-08 | C | T | 0.000548151364659465 | 31.9648794273622 | 0.4781 | C | T |
| rs5754104 | 22 | 1.355e-10 | A | G | 0.000704482804846108 | 41.0876123484325 | 0.429 | A | G |
| rs2069235 | 22 | 1.69005e-20 | A | G | 0.0014681367501917 | 85.6917532868614 | 0.00981703 | A | G |
| rs4602367 | 3 | 1.75699e-10 | G | A | 0.000704523323802617 | 41.0899772051797 | 0.7472 | G | A |
| rs3806624 | 3 | 3.93641e-11 | G | A | 0.00074405731438719 | 43.3974385787144 | 0.8174 | G | A |
| rs13103285 | 4 | 4.29042e-14 | T | C | 0.00097695857072941 | 56.9947809589962 | 0.731499 | T | C |
| rs34046593 | 4 | 7.16638e-17 | A | G | 0.00119903249995213 | 69.9659035544603 | 0.7949 | A | G |
| rs2918392 | 5 | 4.62104e-08 | C | T | 0.000514115359899186 | 29.9790841132627 | 0.3091 | C | T |
| rs7731626 | 5 | 1.94089e-26 | A | G | 0.00193513591072256 | 113.002265891452 | 0.3967 | A | G |
| rs403214 | 5 | 3.95804e-10 | G | A | 0.000671963065116686 | 39.1896853822411 | 0.9905 | G | A |
| rs244685 | 5 | 6.03601e-10 | G | T | 0.00065496959057473 | 38.1979561775948 | 0.2375 | G | T |
| rs9405192 | 6 | 9.25977e-11 | A | G | 0.000723561044714213 | 42.2011198944327 | 0.9884 | A | G |
| rs12530098 | 6 | 1.35394e-11 | T | C | 0.000786800385694845 | 45.8924082435734 | 0.1089 | T | C |
| rs3025669 | 6 | 2.91273e-30 | G | C | 0.00223042784521827 | 130.284385596443 | 0.8147 | G | C |
| rs1611236 | 6 | 4.5436e-19 | A | G | 0.00135509891890628 | 79.0850432483033 | 0.6711 | A | G |
| rs9258357 | 6 | 5.76501e-22 | C | T | 0.00159842263977987 | 93.3084145739876 | 0.1117 | C | T |
| rs11754264 | 6 | 1.88105e-12 | C | T | 0.000849974017077673 | 49.5803276535847 | 0.248 | C | T |
| rs112733823 | 6 | 3.82032e-24 | T | C | 0.00176780053508964 | 103.21341151019 | 0.8885 | T | C |
| rs139395255 | 6 | 8.6836e-60 | G | A | 0.00454375326935948 | 266.027792697619 | 0.1767 | G | A |
| rs114508013 | 6 | 1.81593e-28 | A | G | 0.00209653273183503 | 122.446834473192 | 0.2673 | A | G |
| rs115521560 | 6 | 1.29122e-103 | C | A | 0.0079726642579076 | 468.397191829171 | 0.0176299 | C | A |
| rs71565312 | 6 | 1.1051e-67 | A | G | 0.00516066708279514 | 302.334245307225 | 0.1951 | A | G |
| rs146305655 | 6 | 3.29079e-22 | A | G | 0.00160777348524116 | 93.8551520918116 | 0.204 | A | G |
| rs5020946 | 6 | 1e-200 | T | G | 0.0248937958134512 | 1487.89967838416 | 0.1067 | T | G |
| rs9271365 | 6 | 1e-200 | G | T | 0.0244095978658332 | 1458.23511558168 | 0.6003 | G | T |
| rs2233424 | 6 | 6.49232e-26 | T | C | 0.0018889882274731 | 110.302371755295 | 0.3392 | T | C |
| rs76153210 | 6 | 6.83282e-15 | T | C | 0.00104016153387336 | 60.6858175702955 | 0.1024 | T | C |
| rs62422878 | 6 | 3.57396e-09 | T | C | 0.000595284016876986 | 34.7150083612477 | 0.3032 | T | C |
| rs7749323 | 6 | 3.46817e-29 | A | G | 0.0021497161530944 | 125.559674495087 | 0.3412 | A | G |
| rs212389 | 6 | 6.65733e-13 | A | G | 0.000887976559838028 | 51.7990461993269 | 0.8667 | A | G |
| rs1571878 | 6 | 4.12572e-40 | T | C | 0.00301094140472042 | 176.013653747781 | 0.758799 | T | C |
| rs740122 | 7 | 5.37205e-09 | A | G | 0.000583983887497936 | 34.0556368743678 | 0.519501 | A | G |
| rs42034 | 7 | 1.28499e-08 | G | A | 0.000555728515629577 | 32.4069788301643 | 0.721099 | G | A |
| rs3757387 | 7 | 1.86681e-19 | C | T | 0.00139457020037203 | 81.3918470625492 | 0.3051 | C | T |
| rs9693589 | 8 | 1.49589e-18 | A | G | 0.00132831561999551 | 77.5198618078778 | 0.7842 | A | G |
| rs11574914 | 9 | 9.91745e-15 | A | G | 0.00102633884407141 | 59.8785361777755 | 0.8337 | A | G |
| rs10435844 | 9 | 9.72971e-11 | T | G | 0.000719779256577692 | 41.9803912466634 | 0.1085 | T | G |

**RA and IL-6**

| SNP | chr.exposure | pval.exposure | effect_allele.exposure | other_allele.exposure | r2 | F | pval.outcome | effect_allele.outcome | other_allele.outcome |
| --- | --- | --- | --- | --- | --- | --- | --- | --- | --- |
| rs2258734 | 1 | 6.04366e-14 | A | G | 0.000961041434499621 | 56.0652979598839 | 0.9877 | A | G |
| rs2076616 | 1 | 6.20297e-11 | G | A | 0.000736799884008522 | 42.9738339556586 | 0.1737 | G | A |
| rs2301888 | 1 | 3.75232e-26 | A | G | 0.0019222962296568 | 112.251048624403 | 0.5923 | A | G |
| rs28411352 | 1 | 1.66418e-11 | T | C | 0.000774334849760245 | 45.164756358556 | 0.1143 | T | C |
| rs6679677 | 1 | 1.40929e-145 | A | C | 0.0112015396813439 | 660.243883771518 | 0.5322 | A | C |
| rs12126142 | 1 | 1.013e-10 | A | G | 0.00071862502862948 | 41.9130236664156 | 0.000184001 | A | G |
| rs3761959 | 1 | 9.64495e-11 | T | C | 0.000717610978962601 | 41.8538378494508 | 0.3191 | T | C |
| rs1234313 | 1 | 1.89701e-09 | G | A | 0.000615738794392477 | 35.9085987320739 | 0.703701 | G | A |
| rs61828284 | 1 | 6.32805e-09 | T | C | 0.000576612422506277 | 33.6255140976527 | 0.8226 | T | C |
| rs10911902 | 1 | 2.357e-08 | T | C | 0.00053247470649769 | 31.0502243031709 | 0.815 | T | C |
| rs3134883 | 10 | 1.98381e-15 | A | G | 0.00107723348509969 | 62.851027209663 | 0.5047 | A | G |
| rs502919 | 10 | 6.17007e-10 | C | T | 0.000656243505366125 | 38.2722999280117 | 0.4435 | C | T |
| rs2275806 | 10 | 2.51397e-09 | A | G | 0.000605541537591372 | 35.3135557187281 | 0.1118 | A | G |
| rs1538981 | 10 | 4.41601e-09 | T | C | 0.000594056723991988 | 34.6433941289253 | 0.754699 | T | C |
| rs7097397 | 10 | 1.41612e-12 | A | G | 0.000854051207089392 | 49.8183598819764 | 0.4672 | A | G |
| rs71508903 | 10 | 3.12608e-25 | T | C | 0.00185180621703494 | 108.127200563464 | 0.0311401 | T | C |
| rs6479800 | 10 | 3.01093e-11 | C | G | 0.000756090786340865 | 44.0998266821519 | 0.9788 | C | G |
| rs9943599 | 11 | 2.70402e-10 | T | C | 0.00068828011840664 | 40.1419707813783 | 0.2866 | T | C |
| rs660442 | 11 | 1.11301e-09 | A | G | 0.000637421158338194 | 37.1738753649617 | 0.8059 | A | G |
| rs34502849 | 11 | 1.068e-09 | A | G | 0.000633546456743195 | 36.9477627160603 | 0.9402 | A | G |
| rs4409785 | 11 | 7.84802e-09 | C | T | 0.000572172816140967 | 33.3664674559768 | 0.2935 | C | T |
| rs6421571 | 11 | 5.57057e-14 | C | T | 0.000971400144704648 | 56.6701926670335 | 0.05254 | C | T |
| rs7105899 | 11 | 4.63895e-10 | A | G | 0.000664750328436899 | 38.7687501813755 | 0.6716 | A | G |
| rs4963581 | 12 | 3.75301e-08 | A | G | 0.00051632682328896 | 30.1081055374145 | 0.1496 | A | G |
| rs4622308 | 12 | 2.20699e-12 | T | C | 0.000845769827270578 | 49.3348830284812 | 0.6583 | T | C |
| rs9532434 | 13 | 1.94089e-19 | C | T | 0.00140252214664057 | 81.8566014468835 | 0.4825 | C | T |
| rs1950897 | 14 | 1.02212e-13 | T | C | 0.000944649342905473 | 55.1081108439142 | 0.5472 | T | C |
| rs1595260 | 14 | 2.28613e-11 | T | A | 0.00077105965920406 | 44.9735763681987 | 0.2157 | T | A |
| rs2841275 | 14 | 1.7108e-19 | C | A | 0.00139816037628769 | 81.601675279814 | 0.7402 | C | A |
| rs8032939 | 15 | 4.46581e-24 | C | T | 0.00175194175045908 | 102.285867982857 | 0.9033 | C | T |
| rs7170107 | 15 | 6.11223e-18 | T | C | 0.00128079986879687 | 74.7433091738027 | 0.5164 | T | C |
| rs7206670 | 16 | 4.142e-09 | T | G | 0.000595023709630473 | 34.6998190597437 | 0.0526296 | T | G |
| rs12918327 | 16 | 3.04299e-08 | T | C | 0.00052295264721102 | 30.4946734549618 | 0.00438097 | T | C |
| rs9927316 | 16 | 2.30303e-11 | G | C | 0.000760849344255047 | 44.3775861391863 | 0.8149 | G | C |
| rs4795400 | 17 | 5.85504e-10 | T | C | 0.000657325104866287 | 38.3354205961804 | 0.3835 | T | C |
| rs2847297 | 18 | 2.64972e-14 | G | A | 0.000986968703644098 | 57.5793389913463 | 0.9248 | G | A |
| rs34536443 | 19 | 1.07895e-15 | C | G | 0.00110207415477557 | 64.3019514073775 | 0.7971 | C | G |
| rs1355208 | 2 | 6.76862e-12 | G | A | 0.000810049190921235 | 47.2495614142664 | 0.619001 | G | A |
| rs12466919 | 2 | 1.58891e-11 | T | C | 0.000779601196224082 | 45.4721670741779 | 0.69 | T | C |
| rs1858037 | 2 | 1.14393e-14 | A | T | 0.00102287985551872 | 59.6765256552822 | 0.4543 | A | T |
| rs28421442 | 2 | 7.86303e-09 | A | T | 0.000570171795089792 | 33.2497106086072 | 0.0461296 | A | T |
| rs11123811 | 2 | 2.01094e-18 | C | T | 0.00130532856512629 | 76.1765949180311 | 0.752199 | C | T |
| rs11889341 | 2 | 4.32215e-30 | T | C | 0.00221094483306058 | 129.143816614502 | 0.650801 | T | C |
| rs3087243 | 2 | 3.31513e-24 | A | G | 0.00177119977751859 | 103.412229150603 | 0.663201 | A | G |
| rs1883832 | 20 | 1.13006e-16 | C | T | 0.00117588190877531 | 68.6134306991008 | 0.5568 | C | T |
| rs6011186 | 20 | 3.19396e-10 | T | C | 0.000676352589078809 | 39.4458609068439 | 0.2931 | T | C |
| rs8126756 | 21 | 1.81401e-09 | C | T | 0.00061878584168435 | 36.0864061822702 | 0.2249 | C | T |
| rs1893592 | 21 | 1.48115e-13 | C | A | 0.000937120085919884 | 54.6684637630414 | 0.705601 | C | A |
| rs7278257 | 21 | 4.27504e-10 | C | G | 0.000670135275503143 | 39.0830150339214 | 0.9356 | C | G |
| rs2073609 | 21 | 1.46501e-08 | C | T | 0.000548151364659465 | 31.9648794273622 | 0.2176 | C | T |
| rs5754104 | 22 | 1.355e-10 | A | G | 0.000704482804846108 | 41.0876123484325 | 0.7124 | A | G |
| rs2069235 | 22 | 1.69005e-20 | A | G | 0.0014681367501917 | 85.6917532868614 | 0.0109001 | A | G |
| rs4602367 | 3 | 1.75699e-10 | G | A | 0.000704523323802617 | 41.0899772051797 | 0.6645 | G | A |
| rs3806624 | 3 | 3.93641e-11 | G | A | 0.00074405731438719 | 43.3974385787144 | 0.8111 | G | A |
| rs13103285 | 4 | 4.29042e-14 | T | C | 0.00097695857072941 | 56.9947809589962 | 0.450001 | T | C |
| rs34046593 | 4 | 7.16638e-17 | A | G | 0.00119903249995213 | 69.9659035544603 | 0.2021 | A | G |
| rs2918392 | 5 | 4.62104e-08 | C | T | 0.000514115359899186 | 29.9790841132627 | 0.2073 | C | T |
| rs7731626 | 5 | 1.94089e-26 | A | G | 0.00193513591072256 | 113.002265891452 | 0.4394 | A | G |
| rs403214 | 5 | 3.95804e-10 | G | A | 0.000671963065116686 | 39.1896853822411 | 0.8884 | G | A |
| rs244685 | 5 | 6.03601e-10 | G | T | 0.00065496959057473 | 38.1979561775948 | 0.7428 | G | T |
| rs9405192 | 6 | 9.25977e-11 | A | G | 0.000723561044714213 | 42.2011198944327 | 0.2169 | A | G |
| rs12530098 | 6 | 1.35394e-11 | T | C | 0.000786800385694845 | 45.8924082435734 | 0.510199 | T | C |
| rs3025669 | 6 | 2.91273e-30 | G | C | 0.00223042784521827 | 130.284385596443 | 0.7538 | G | C |
| rs1611236 | 6 | 4.5436e-19 | A | G | 0.00135509891890628 | 79.0850432483033 | 0.696599 | A | G |
| rs9258357 | 6 | 5.76501e-22 | C | T | 0.00159842263977987 | 93.3084145739876 | 0.5278 | C | T |
| rs11754264 | 6 | 1.88105e-12 | C | T | 0.000849974017077673 | 49.5803276535847 | 0.8442 | C | T |
| rs112733823 | 6 | 3.82032e-24 | T | C | 0.00176780053508964 | 103.21341151019 | 0.788401 | T | C |
| rs139395255 | 6 | 8.6836e-60 | G | A | 0.00454375326935948 | 266.027792697619 | 0.768599 | G | A |
| rs114508013 | 6 | 1.81593e-28 | A | G | 0.00209653273183503 | 122.446834473192 | 0.1033 | A | G |
| rs115521560 | 6 | 1.29122e-103 | C | A | 0.0079726642579076 | 468.397191829171 | 0.2946 | C | A |
| rs71565312 | 6 | 1.1051e-67 | A | G | 0.00516066708279514 | 302.334245307225 | 0.4683 | A | G |
| rs146305655 | 6 | 3.29079e-22 | A | G | 0.00160777348524116 | 93.8551520918116 | 0.5256 | A | G |
| rs5020946 | 6 | 1e-200 | T | G | 0.0248937958134512 | 1487.89967838416 | 0.4475 | T | G |
| rs9271365 | 6 | 1e-200 | G | T | 0.0244095978658332 | 1458.23511558168 | 0.5662 | G | T |
| rs2233424 | 6 | 6.49232e-26 | T | C | 0.0018889882274731 | 110.302371755295 | 0.3 | T | C |
| rs76153210 | 6 | 6.83282e-15 | T | C | 0.00104016153387336 | 60.6858175702955 | 0.0373104 | T | C |
| rs62422878 | 6 | 3.57396e-09 | T | C | 0.000595284016876986 | 34.7150083612477 | 0.7375 | T | C |
| rs7749323 | 6 | 3.46817e-29 | A | G | 0.0021497161530944 | 125.559674495087 | 0.5952 | A | G |
| rs212389 | 6 | 6.65733e-13 | A | G | 0.000887976559838028 | 51.7990461993269 | 0.9817 | A | G |
| rs1571878 | 6 | 4.12572e-40 | T | C | 0.00301094140472042 | 176.013653747781 | 0.6003 | T | C |
| rs740122 | 7 | 5.37205e-09 | A | G | 0.000583983887497936 | 34.0556368743678 | 0.9438 | A | G |
| rs42034 | 7 | 1.28499e-08 | G | A | 0.000555728515629577 | 32.4069788301643 | 0.0953103 | G | A |
| rs3757387 | 7 | 1.86681e-19 | C | T | 0.00139457020037203 | 81.3918470625492 | 0.2147 | C | T |
| rs9693589 | 8 | 1.49589e-18 | A | G | 0.00132831561999551 | 77.5198618078778 | 0.8186 | A | G |
| rs11574914 | 9 | 9.91745e-15 | A | G | 0.00102633884407141 | 59.8785361777755 | 0.6081 | A | G |
| rs10435844 | 9 | 9.72971e-11 | T | G | 0.000719779256577692 | 41.9803912466634 | 0.6436 | T | G |

**RA and IL-5**

| SNP | chr.exposure | pval.exposure | effect_allele.exposure | other_allele.exposure | r2 | F | pval.outcome | effect_allele.outcome | other_allele.outcome |
| --- | --- | --- | --- | --- | --- | --- | --- | --- | --- |
| rs2258734 | 1 | 6.04366e-14 | A | G | 0.000961041434499621 | 56.0652979598839 | 0.8251 | A | G |
| rs2076616 | 1 | 6.20297e-11 | G | A | 0.000736799884008522 | 42.9738339556586 | 0.0576103 | G | A |
| rs2301888 | 1 | 3.75232e-26 | A | G | 0.0019222962296568 | 112.251048624403 | 0.4335 | A | G |
| rs28411352 | 1 | 1.66418e-11 | T | C | 0.000774334849760245 | 45.164756358556 | 0.2243 | T | C |
| rs6679677 | 1 | 1.40929e-145 | A | C | 0.0112015396813439 | 660.243883771518 | 0.0663896 | A | C |
| rs12126142 | 1 | 1.013e-10 | A | G | 0.00071862502862948 | 41.9130236664156 | 0.0812101 | A | G |
| rs3761959 | 1 | 9.64495e-11 | T | C | 0.000717610978962601 | 41.8538378494508 | 0.8348 | T | C |
| rs1234313 | 1 | 1.89701e-09 | G | A | 0.000615738794392477 | 35.9085987320739 | 0.8425 | G | A |
| rs61828284 | 1 | 6.32805e-09 | T | C | 0.000576612422506277 | 33.6255140976527 | 0.5824 | T | C |
| rs10911902 | 1 | 2.357e-08 | T | C | 0.00053247470649769 | 31.0502243031709 | 0.1101 | T | C |
| rs3134883 | 10 | 1.98381e-15 | A | G | 0.00107723348509969 | 62.851027209663 | 0.406 | A | G |
| rs502919 | 10 | 6.17007e-10 | C | T | 0.000656243505366125 | 38.2722999280117 | 0.444 | C | T |
| rs2275806 | 10 | 2.51397e-09 | A | G | 0.000605541537591372 | 35.3135557187281 | 0.7892 | A | G |
| rs1538981 | 10 | 4.41601e-09 | T | C | 0.000594056723991988 | 34.6433941289253 | 0.5278 | T | C |
| rs7097397 | 10 | 1.41612e-12 | A | G | 0.000854051207089392 | 49.8183598819764 | 0.2395 | A | G |
| rs71508903 | 10 | 3.12608e-25 | T | C | 0.00185180621703494 | 108.127200563464 | 0.4695 | T | C |
| rs6479800 | 10 | 3.01093e-11 | C | G | 0.000756090786340865 | 44.0998266821519 | 0.288 | C | G |
| rs9943599 | 11 | 2.70402e-10 | T | C | 0.00068828011840664 | 40.1419707813783 | 0.8918 | T | C |
| rs660442 | 11 | 1.11301e-09 | A | G | 0.000637421158338194 | 37.1738753649617 | 0.4501 | A | G |
| rs34502849 | 11 | 1.068e-09 | A | G | 0.000633546456743195 | 36.9477627160603 | 0.8607 | A | G |
| rs4409785 | 11 | 7.84802e-09 | C | T | 0.000572172816140967 | 33.3664674559768 | 0.9342 | C | T |
| rs6421571 | 11 | 5.57057e-14 | C | T | 0.000971400144704648 | 56.6701926670335 | 0.2029 | C | T |
| rs7105899 | 11 | 4.63895e-10 | A | G | 0.000664750328436899 | 38.7687501813755 | 0.7363 | A | G |
| rs4963581 | 12 | 3.75301e-08 | A | G | 0.00051632682328896 | 30.1081055374145 | 0.0606401 | A | G |
| rs4622308 | 12 | 2.20699e-12 | T | C | 0.000845769827270578 | 49.3348830284812 | 0.4228 | T | C |
| rs9532434 | 13 | 1.94089e-19 | C | T | 0.00140252214664057 | 81.8566014468835 | 0.7867 | C | T |
| rs1950897 | 14 | 1.02212e-13 | T | C | 0.000944649342905473 | 55.1081108439142 | 0.8207 | T | C |
| rs1595260 | 14 | 2.28613e-11 | T | A | 0.00077105965920406 | 44.9735763681987 | 0.0306902 | T | A |
| rs2841275 | 14 | 1.7108e-19 | C | A | 0.00139816037628769 | 81.601675279814 | 0.1899 | C | A |
| rs8032939 | 15 | 4.46581e-24 | C | T | 0.00175194175045908 | 102.285867982857 | 0.9101 | C | T |
| rs7170107 | 15 | 6.11223e-18 | T | C | 0.00128079986879687 | 74.7433091738027 | 0.5567 | T | C |
| rs7206670 | 16 | 4.142e-09 | T | G | 0.000595023709630473 | 34.6998190597437 | 0.2151 | T | G |
| rs12918327 | 16 | 3.04299e-08 | T | C | 0.00052295264721102 | 30.4946734549618 | 0.1518 | T | C |
| rs9927316 | 16 | 2.30303e-11 | G | C | 0.000760849344255047 | 44.3775861391863 | 0.7113 | G | C |
| rs4795400 | 17 | 5.85504e-10 | T | C | 0.000657325104866287 | 38.3354205961804 | 0.9742 | T | C |
| rs2847297 | 18 | 2.64972e-14 | G | A | 0.000986968703644098 | 57.5793389913463 | 0.5001 | G | A |
| rs34536443 | 19 | 1.07895e-15 | C | G | 0.00110207415477557 | 64.3019514073775 | 0.4617 | C | G |
| rs1355208 | 2 | 6.76862e-12 | G | A | 0.000810049190921235 | 47.2495614142664 | 0.1655 | G | A |
| rs12466919 | 2 | 1.58891e-11 | T | C | 0.000779601196224082 | 45.4721670741779 | 0.294 | T | C |
| rs1858037 | 2 | 1.14393e-14 | A | T | 0.00102287985551872 | 59.6765256552822 | 0.4704 | A | T |
| rs28421442 | 2 | 7.86303e-09 | A | T | 0.000570171795089792 | 33.2497106086072 | 0.582101 | A | T |
| rs11123811 | 2 | 2.01094e-18 | C | T | 0.00130532856512629 | 76.1765949180311 | 0.8878 | C | T |
| rs11889341 | 2 | 4.32215e-30 | T | C | 0.00221094483306058 | 129.143816614502 | 0.518601 | T | C |
| rs3087243 | 2 | 3.31513e-24 | A | G | 0.00177119977751859 | 103.412229150603 | 0.5904 | A | G |
| rs1883832 | 20 | 1.13006e-16 | C | T | 0.00117588190877531 | 68.6134306991008 | 0.3685 | C | T |
| rs6011186 | 20 | 3.19396e-10 | T | C | 0.000676352589078809 | 39.4458609068439 | 0.0239701 | T | C |
| rs8126756 | 21 | 1.81401e-09 | C | T | 0.00061878584168435 | 36.0864061822702 | 0.490801 | C | T |
| rs1893592 | 21 | 1.48115e-13 | C | A | 0.000937120085919884 | 54.6684637630414 | 0.5642 | C | A |
| rs7278257 | 21 | 4.27504e-10 | C | G | 0.000670135275503143 | 39.0830150339214 | 0.4737 | C | G |
| rs2073609 | 21 | 1.46501e-08 | C | T | 0.000548151364659465 | 31.9648794273622 | 0.5165 | C | T |
| rs5754104 | 22 | 1.355e-10 | A | G | 0.000704482804846108 | 41.0876123484325 | 0.5418 | A | G |
| rs2069235 | 22 | 1.69005e-20 | A | G | 0.0014681367501917 | 85.6917532868614 | 0.2242 | A | G |
| rs4602367 | 3 | 1.75699e-10 | G | A | 0.000704523323802617 | 41.0899772051797 | 0.763801 | G | A |
| rs3806624 | 3 | 3.93641e-11 | G | A | 0.00074405731438719 | 43.3974385787144 | 0.9119 | G | A |
| rs13103285 | 4 | 4.29042e-14 | T | C | 0.00097695857072941 | 56.9947809589962 | 0.02044 | T | C |
| rs34046593 | 4 | 7.16638e-17 | A | G | 0.00119903249995213 | 69.9659035544603 | 0.3765 | A | G |
| rs2918392 | 5 | 4.62104e-08 | C | T | 0.000514115359899186 | 29.9790841132627 | 0.0678094 | C | T |
| rs7731626 | 5 | 1.94089e-26 | A | G | 0.00193513591072256 | 113.002265891452 | 0.0402903 | A | G |
| rs403214 | 5 | 3.95804e-10 | G | A | 0.000671963065116686 | 39.1896853822411 | 0.8361 | G | A |
| rs244685 | 5 | 6.03601e-10 | G | T | 0.00065496959057473 | 38.1979561775948 | 0.001508 | G | T |
| rs9405192 | 6 | 9.25977e-11 | A | G | 0.000723561044714213 | 42.2011198944327 | 0.2319 | A | G |
| rs12530098 | 6 | 1.35394e-11 | T | C | 0.000786800385694845 | 45.8924082435734 | 0.8018 | T | C |
| rs3025669 | 6 | 2.91273e-30 | G | C | 0.00223042784521827 | 130.284385596443 | 0.0422105 | G | C |
| rs1611236 | 6 | 4.5436e-19 | A | G | 0.00135509891890628 | 79.0850432483033 | 0.9978 | A | G |
| rs9258357 | 6 | 5.76501e-22 | C | T | 0.00159842263977987 | 93.3084145739876 | 0.0202698 | C | T |
| rs11754264 | 6 | 1.88105e-12 | C | T | 0.000849974017077673 | 49.5803276535847 | 0.624701 | C | T |
| rs112733823 | 6 | 3.82032e-24 | T | C | 0.00176780053508964 | 103.21341151019 | 0.2684 | T | C |
| rs139395255 | 6 | 8.6836e-60 | G | A | 0.00454375326935948 | 266.027792697619 | 0.2123 | G | A |
| rs114508013 | 6 | 1.81593e-28 | A | G | 0.00209653273183503 | 122.446834473192 | 0.685799 | A | G |
| rs115521560 | 6 | 1.29122e-103 | C | A | 0.0079726642579076 | 468.397191829171 | 0.0803508 | C | A |
| rs71565312 | 6 | 1.1051e-67 | A | G | 0.00516066708279514 | 302.334245307225 | 0.1892 | A | G |
| rs146305655 | 6 | 3.29079e-22 | A | G | 0.00160777348524116 | 93.8551520918116 | 0.6323 | A | G |
| rs5020946 | 6 | 1e-200 | T | G | 0.0248937958134512 | 1487.89967838416 | 0.1076 | T | G |
| rs9271365 | 6 | 1e-200 | G | T | 0.0244095978658332 | 1458.23511558168 | 0.1771 | G | T |
| rs2233424 | 6 | 6.49232e-26 | T | C | 0.0018889882274731 | 110.302371755295 | 0.6543 | T | C |
| rs76153210 | 6 | 6.83282e-15 | T | C | 0.00104016153387336 | 60.6858175702955 | 0.6392 | T | C |
| rs62422878 | 6 | 3.57396e-09 | T | C | 0.000595284016876986 | 34.7150083612477 | 0.1702 | T | C |
| rs7749323 | 6 | 3.46817e-29 | A | G | 0.0021497161530944 | 125.559674495087 | 0.8712 | A | G |
| rs212389 | 6 | 6.65733e-13 | A | G | 0.000887976559838028 | 51.7990461993269 | 0.5739 | A | G |
| rs1571878 | 6 | 4.12572e-40 | T | C | 0.00301094140472042 | 176.013653747781 | 0.6093 | T | C |
| rs740122 | 7 | 5.37205e-09 | A | G | 0.000583983887497936 | 34.0556368743678 | 0.3026 | A | G |
| rs42034 | 7 | 1.28499e-08 | G | A | 0.000555728515629577 | 32.4069788301643 | 0.6006 | G | A |
| rs3757387 | 7 | 1.86681e-19 | C | T | 0.00139457020037203 | 81.3918470625492 | 0.6591 | C | T |
| rs9693589 | 8 | 1.49589e-18 | A | G | 0.00132831561999551 | 77.5198618078778 | 0.6367 | A | G |
| rs11574914 | 9 | 9.91745e-15 | A | G | 0.00102633884407141 | 59.8785361777755 | 0.1991 | A | G |
| rs10435844 | 9 | 9.72971e-11 | T | G | 0.000719779256577692 | 41.9803912466634 | 0.7955 | T | G |

**RA and IL-4**

| SNP | chr.exposure | pval.exposure | effect_allele.exposure | other_allele.exposure | r2 | F | pval.outcome | effect_allele.outcome | other_allele.outcome |
| --- | --- | --- | --- | --- | --- | --- | --- | --- | --- |
| rs2258734 | 1 | 6.04366e-14 | A | G | 0.000961041434499621 | 56.0652979598839 | 0.8485 | A | G |
| rs2076616 | 1 | 6.20297e-11 | G | A | 0.000736799884008522 | 42.9738339556586 | 0.8396 | G | A |
| rs2301888 | 1 | 3.75232e-26 | A | G | 0.0019222962296568 | 112.251048624403 | 0.2999 | A | G |
| rs28411352 | 1 | 1.66418e-11 | T | C | 0.000774334849760245 | 45.164756358556 | 0.1158 | T | C |
| rs6679677 | 1 | 1.40929e-145 | A | C | 0.0112015396813439 | 660.243883771518 | 0.4607 | A | C |
| rs12126142 | 1 | 1.013e-10 | A | G | 0.00071862502862948 | 41.9130236664156 | 0.00851609 | A | G |
| rs3761959 | 1 | 9.64495e-11 | T | C | 0.000717610978962601 | 41.8538378494508 | 0.0387704 | T | C |
| rs1234313 | 1 | 1.89701e-09 | G | A | 0.000615738794392477 | 35.9085987320739 | 0.2808 | G | A |
| rs61828284 | 1 | 6.32805e-09 | T | C | 0.000576612422506277 | 33.6255140976527 | 0.296 | T | C |
| rs10911902 | 1 | 2.357e-08 | T | C | 0.00053247470649769 | 31.0502243031709 | 0.5144 | T | C |
| rs3134883 | 10 | 1.98381e-15 | A | G | 0.00107723348509969 | 62.851027209663 | 0.8738 | A | G |
| rs502919 | 10 | 6.17007e-10 | C | T | 0.000656243505366125 | 38.2722999280117 | 0.0661607 | C | T |
| rs2275806 | 10 | 2.51397e-09 | A | G | 0.000605541537591372 | 35.3135557187281 | 0.609799 | A | G |
| rs1538981 | 10 | 4.41601e-09 | T | C | 0.000594056723991988 | 34.6433941289253 | 0.1618 | T | C |
| rs7097397 | 10 | 1.41612e-12 | A | G | 0.000854051207089392 | 49.8183598819764 | 0.276 | A | G |
| rs71508903 | 10 | 3.12608e-25 | T | C | 0.00185180621703494 | 108.127200563464 | 0.00110301 | T | C |
| rs6479800 | 10 | 3.01093e-11 | C | G | 0.000756090786340865 | 44.0998266821519 | 0.5523 | C | G |
| rs9943599 | 11 | 2.70402e-10 | T | C | 0.00068828011840664 | 40.1419707813783 | 0.4885 | T | C |
| rs660442 | 11 | 1.11301e-09 | A | G | 0.000637421158338194 | 37.1738753649617 | 0.5192 | A | G |
| rs34502849 | 11 | 1.068e-09 | A | G | 0.000633546456743195 | 36.9477627160603 | 0.5131 | A | G |
| rs4409785 | 11 | 7.84802e-09 | C | T | 0.000572172816140967 | 33.3664674559768 | 0.4017 | C | T |
| rs6421571 | 11 | 5.57057e-14 | C | T | 0.000971400144704648 | 56.6701926670335 | 0.5037 | C | T |
| rs7105899 | 11 | 4.63895e-10 | A | G | 0.000664750328436899 | 38.7687501813755 | 0.9077 | A | G |
| rs4963581 | 12 | 3.75301e-08 | A | G | 0.00051632682328896 | 30.1081055374145 | 0.7944 | A | G |
| rs4622308 | 12 | 2.20699e-12 | T | C | 0.000845769827270578 | 49.3348830284812 | 0.425 | T | C |
| rs9532434 | 13 | 1.94089e-19 | C | T | 0.00140252214664057 | 81.8566014468835 | 0.7173 | C | T |
| rs1950897 | 14 | 1.02212e-13 | T | C | 0.000944649342905473 | 55.1081108439142 | 0.8688 | T | C |
| rs1595260 | 14 | 2.28613e-11 | T | A | 0.00077105965920406 | 44.9735763681987 | 0.2595 | T | A |
| rs2841275 | 14 | 1.7108e-19 | C | A | 0.00139816037628769 | 81.601675279814 | 0.6386 | C | A |
| rs8032939 | 15 | 4.46581e-24 | C | T | 0.00175194175045908 | 102.285867982857 | 0.9007 | C | T |
| rs7170107 | 15 | 6.11223e-18 | T | C | 0.00128079986879687 | 74.7433091738027 | 0.6338 | T | C |
| rs7206670 | 16 | 4.142e-09 | T | G | 0.000595023709630473 | 34.6998190597437 | 0.1123 | T | G |
| rs12918327 | 16 | 3.04299e-08 | T | C | 0.00052295264721102 | 30.4946734549618 | 0.2526 | T | C |
| rs9927316 | 16 | 2.30303e-11 | G | C | 0.000760849344255047 | 44.3775861391863 | 0.8319 | G | C |
| rs4795400 | 17 | 5.85504e-10 | T | C | 0.000657325104866287 | 38.3354205961804 | 0.9583 | T | C |
| rs2847297 | 18 | 2.64972e-14 | G | A | 0.000986968703644098 | 57.5793389913463 | 0.755899 | G | A |
| rs34536443 | 19 | 1.07895e-15 | C | G | 0.00110207415477557 | 64.3019514073775 | 0.2726 | C | G |
| rs1355208 | 2 | 6.76862e-12 | G | A | 0.000810049190921235 | 47.2495614142664 | 0.756599 | G | A |
| rs12466919 | 2 | 1.58891e-11 | T | C | 0.000779601196224082 | 45.4721670741779 | 0.9009 | T | C |
| rs1858037 | 2 | 1.14393e-14 | A | T | 0.00102287985551872 | 59.6765256552822 | 0.8843 | A | T |
| rs28421442 | 2 | 7.86303e-09 | A | T | 0.000570171795089792 | 33.2497106086072 | 0.1091 | A | T |
| rs11123811 | 2 | 2.01094e-18 | C | T | 0.00130532856512629 | 76.1765949180311 | 0.659699 | C | T |
| rs11889341 | 2 | 4.32215e-30 | T | C | 0.00221094483306058 | 129.143816614502 | 0.2605 | T | C |
| rs3087243 | 2 | 3.31513e-24 | A | G | 0.00177119977751859 | 103.412229150603 | 0.6404 | A | G |
| rs1883832 | 20 | 1.13006e-16 | C | T | 0.00117588190877531 | 68.6134306991008 | 0.5364 | C | T |
| rs6011186 | 20 | 3.19396e-10 | T | C | 0.000676352589078809 | 39.4458609068439 | 0.1681 | T | C |
| rs8126756 | 21 | 1.81401e-09 | C | T | 0.00061878584168435 | 36.0864061822702 | 0.2601 | C | T |
| rs1893592 | 21 | 1.48115e-13 | C | A | 0.000937120085919884 | 54.6684637630414 | 0.9728 | C | A |
| rs7278257 | 21 | 4.27504e-10 | C | G | 0.000670135275503143 | 39.0830150339214 | 0.686099 | C | G |
| rs2073609 | 21 | 1.46501e-08 | C | T | 0.000548151364659465 | 31.9648794273622 | 0.9565 | C | T |
| rs5754104 | 22 | 1.355e-10 | A | G | 0.000704482804846108 | 41.0876123484325 | 0.8854 | A | G |
| rs2069235 | 22 | 1.69005e-20 | A | G | 0.0014681367501917 | 85.6917532868614 | 0.139 | A | G |
| rs4602367 | 3 | 1.75699e-10 | G | A | 0.000704523323802617 | 41.0899772051797 | 0.543501 | G | A |
| rs3806624 | 3 | 3.93641e-11 | G | A | 0.00074405731438719 | 43.3974385787144 | 0.98 | G | A |
| rs13103285 | 4 | 4.29042e-14 | T | C | 0.00097695857072941 | 56.9947809589962 | 0.1724 | T | C |
| rs34046593 | 4 | 7.16638e-17 | A | G | 0.00119903249995213 | 69.9659035544603 | 0.6462 | A | G |
| rs2918392 | 5 | 4.62104e-08 | C | T | 0.000514115359899186 | 29.9790841132627 | 0.0347896 | C | T |
| rs7731626 | 5 | 1.94089e-26 | A | G | 0.00193513591072256 | 113.002265891452 | 0.1607 | A | G |
| rs403214 | 5 | 3.95804e-10 | G | A | 0.000671963065116686 | 39.1896853822411 | 0.7039 | G | A |
| rs244685 | 5 | 6.03601e-10 | G | T | 0.00065496959057473 | 38.1979561775948 | 0.8773 | G | T |
| rs9405192 | 6 | 9.25977e-11 | A | G | 0.000723561044714213 | 42.2011198944327 | 0.00398602 | A | G |
| rs12530098 | 6 | 1.35394e-11 | T | C | 0.000786800385694845 | 45.8924082435734 | 0.5877 | T | C |
| rs3025669 | 6 | 2.91273e-30 | G | C | 0.00223042784521827 | 130.284385596443 | 0.1043 | G | C |
| rs1611236 | 6 | 4.5436e-19 | A | G | 0.00135509891890628 | 79.0850432483033 | 0.8336 | A | G |
| rs9258357 | 6 | 5.76501e-22 | C | T | 0.00159842263977987 | 93.3084145739876 | 0.0547394 | C | T |
| rs11754264 | 6 | 1.88105e-12 | C | T | 0.000849974017077673 | 49.5803276535847 | 0.9383 | C | T |
| rs112733823 | 6 | 3.82032e-24 | T | C | 0.00176780053508964 | 103.21341151019 | 0.2561 | T | C |
| rs139395255 | 6 | 8.6836e-60 | G | A | 0.00454375326935948 | 266.027792697619 | 0.785599 | G | A |
| rs114508013 | 6 | 1.81593e-28 | A | G | 0.00209653273183503 | 122.446834473192 | 0.001388 | A | G |
| rs115521560 | 6 | 1.29122e-103 | C | A | 0.0079726642579076 | 468.397191829171 | 0.619699 | C | A |
| rs71565312 | 6 | 1.1051e-67 | A | G | 0.00516066708279514 | 302.334245307225 | 0.6575 | A | G |
| rs146305655 | 6 | 3.29079e-22 | A | G | 0.00160777348524116 | 93.8551520918116 | 0.617999 | A | G |
| rs5020946 | 6 | 1e-200 | T | G | 0.0248937958134512 | 1487.89967838416 | 0.7738 | T | G |
| rs9271365 | 6 | 1e-200 | G | T | 0.0244095978658332 | 1458.23511558168 | 0.5874 | G | T |
| rs2233424 | 6 | 6.49232e-26 | T | C | 0.0018889882274731 | 110.302371755295 | 0.8833 | T | C |
| rs76153210 | 6 | 6.83282e-15 | T | C | 0.00104016153387336 | 60.6858175702955 | 0.0775604 | T | C |
| rs62422878 | 6 | 3.57396e-09 | T | C | 0.000595284016876986 | 34.7150083612477 | 0.5923 | T | C |
| rs7749323 | 6 | 3.46817e-29 | A | G | 0.0021497161530944 | 125.559674495087 | 0.5848 | A | G |
| rs212389 | 6 | 6.65733e-13 | A | G | 0.000887976559838028 | 51.7990461993269 | 0.676299 | A | G |
| rs1571878 | 6 | 4.12572e-40 | T | C | 0.00301094140472042 | 176.013653747781 | 0.581599 | T | C |
| rs740122 | 7 | 5.37205e-09 | A | G | 0.000583983887497936 | 34.0556368743678 | 0.9855 | A | G |
| rs42034 | 7 | 1.28499e-08 | G | A | 0.000555728515629577 | 32.4069788301643 | 0.0420504 | G | A |
| rs3757387 | 7 | 1.86681e-19 | C | T | 0.00139457020037203 | 81.3918470625492 | 0.6305 | C | T |
| rs9693589 | 8 | 1.49589e-18 | A | G | 0.00132831561999551 | 77.5198618078778 | 0.3165 | A | G |
| rs11574914 | 9 | 9.91745e-15 | A | G | 0.00102633884407141 | 59.8785361777755 | 0.01516 | A | G |
| rs10435844 | 9 | 9.72971e-11 | T | G | 0.000719779256577692 | 41.9803912466634 | 0.4741 | T | G |

**RA and IL-1β**

| SNP | chr.exposure | pval.exposure | effect_allele.exposure | other_allele.exposure | r2 | F | pval.outcome | effect_allele.outcome | other_allele.outcome |
| --- | --- | --- | --- | --- | --- | --- | --- | --- | --- |
| rs2258734 | 1 | 6.04366e-14 | A | G | 0.000961041434499621 | 56.0652979598839 | 0.1525 | A | G |
| rs2076616 | 1 | 6.20297e-11 | G | A | 0.000736799884008522 | 42.9738339556586 | 0.1951 | G | A |
| rs2301888 | 1 | 3.75232e-26 | A | G | 0.0019222962296568 | 112.251048624403 | 0.0862502 | A | G |
| rs28411352 | 1 | 1.66418e-11 | T | C | 0.000774334849760245 | 45.164756358556 | 0.604399 | T | C |
| rs6679677 | 1 | 1.40929e-145 | A | C | 0.0112015396813439 | 660.243883771518 | 0.2323 | A | C |
| rs12126142 | 1 | 1.013e-10 | A | G | 0.00071862502862948 | 41.9130236664156 | 0.3302 | A | G |
| rs3761959 | 1 | 9.64495e-11 | T | C | 0.000717610978962601 | 41.8538378494508 | 0.3601 | T | C |
| rs1234313 | 1 | 1.89701e-09 | G | A | 0.000615738794392477 | 35.9085987320739 | 0.2456 | G | A |
| rs61828284 | 1 | 6.32805e-09 | T | C | 0.000576612422506277 | 33.6255140976527 | 0.5514 | T | C |
| rs10911902 | 1 | 2.357e-08 | T | C | 0.00053247470649769 | 31.0502243031709 | 0.4219 | T | C |
| rs3134883 | 10 | 1.98381e-15 | A | G | 0.00107723348509969 | 62.851027209663 | 0.6337 | A | G |
| rs502919 | 10 | 6.17007e-10 | C | T | 0.000656243505366125 | 38.2722999280117 | 0.8852 | C | T |
| rs2275806 | 10 | 2.51397e-09 | A | G | 0.000605541537591372 | 35.3135557187281 | 0.3847 | A | G |
| rs1538981 | 10 | 4.41601e-09 | T | C | 0.000594056723991988 | 34.6433941289253 | 0.619699 | T | C |
| rs7097397 | 10 | 1.41612e-12 | A | G | 0.000854051207089392 | 49.8183598819764 | 0.3656 | A | G |
| rs71508903 | 10 | 3.12608e-25 | T | C | 0.00185180621703494 | 108.127200563464 | 0.4921 | T | C |
| rs6479800 | 10 | 3.01093e-11 | C | G | 0.000756090786340865 | 44.0998266821519 | 0.117 | C | G |
| rs9943599 | 11 | 2.70402e-10 | T | C | 0.00068828011840664 | 40.1419707813783 | 0.9872 | T | C |
| rs660442 | 11 | 1.11301e-09 | A | G | 0.000637421158338194 | 37.1738753649617 | 0.9341 | A | G |
| rs34502849 | 11 | 1.068e-09 | A | G | 0.000633546456743195 | 36.9477627160603 | 0.6658 | A | G |
| rs4409785 | 11 | 7.84802e-09 | C | T | 0.000572172816140967 | 33.3664674559768 | 0.1317 | C | T |
| rs6421571 | 11 | 5.57057e-14 | C | T | 0.000971400144704648 | 56.6701926670335 | 0.09356 | C | T |
| rs7105899 | 11 | 4.63895e-10 | A | G | 0.000664750328436899 | 38.7687501813755 | 0.4692 | A | G |
| rs4963581 | 12 | 3.75301e-08 | A | G | 0.00051632682328896 | 30.1081055374145 | 0.391 | A | G |
| rs4622308 | 12 | 2.20699e-12 | T | C | 0.000845769827270578 | 49.3348830284812 | 0.5271 | T | C |
| rs9532434 | 13 | 1.94089e-19 | C | T | 0.00140252214664057 | 81.8566014468835 | 0.4358 | C | T |
| rs1950897 | 14 | 1.02212e-13 | T | C | 0.000944649342905473 | 55.1081108439142 | 0.8397 | T | C |
| rs1595260 | 14 | 2.28613e-11 | T | A | 0.00077105965920406 | 44.9735763681987 | 0.4632 | T | A |
| rs2841275 | 14 | 1.7108e-19 | C | A | 0.00139816037628769 | 81.601675279814 | 0.5551 | C | A |
| rs8032939 | 15 | 4.46581e-24 | C | T | 0.00175194175045908 | 102.285867982857 | 0.4793 | C | T |
| rs7170107 | 15 | 6.11223e-18 | T | C | 0.00128079986879687 | 74.7433091738027 | 0.3957 | T | C |
| rs7206670 | 16 | 4.142e-09 | T | G | 0.000595023709630473 | 34.6998190597437 | 0.1818 | T | G |
| rs12918327 | 16 | 3.04299e-08 | T | C | 0.00052295264721102 | 30.4946734549618 | 0.0887503 | T | C |
| rs9927316 | 16 | 2.30303e-11 | G | C | 0.000760849344255047 | 44.3775861391863 | 0.5247 | G | C |
| rs4795400 | 17 | 5.85504e-10 | T | C | 0.000657325104866287 | 38.3354205961804 | 0.9675 | T | C |
| rs2847297 | 18 | 2.64972e-14 | G | A | 0.000986968703644098 | 57.5793389913463 | 0.5446 | G | A |
| rs34536443 | 19 | 1.07895e-15 | C | G | 0.00110207415477557 | 64.3019514073775 | 0.6187 | C | G |
| rs1355208 | 2 | 6.76862e-12 | G | A | 0.000810049190921235 | 47.2495614142664 | 0.9236 | G | A |
| rs12466919 | 2 | 1.58891e-11 | T | C | 0.000779601196224082 | 45.4721670741779 | 0.3163 | T | C |
| rs1858037 | 2 | 1.14393e-14 | A | T | 0.00102287985551872 | 59.6765256552822 | 0.0256898 | A | T |
| rs28421442 | 2 | 7.86303e-09 | A | T | 0.000570171795089792 | 33.2497106086072 | 0.742099 | A | T |
| rs11123811 | 2 | 2.01094e-18 | C | T | 0.00130532856512629 | 76.1765949180311 | 0.4743 | C | T |
| rs11889341 | 2 | 4.32215e-30 | T | C | 0.00221094483306058 | 129.143816614502 | 0.6421 | T | C |
| rs3087243 | 2 | 3.31513e-24 | A | G | 0.00177119977751859 | 103.412229150603 | 0.0391796 | A | G |
| rs1883832 | 20 | 1.13006e-16 | C | T | 0.00117588190877531 | 68.6134306991008 | 0.683 | C | T |
| rs6011186 | 20 | 3.19396e-10 | T | C | 0.000676352589078809 | 39.4458609068439 | 0.0476398 | T | C |
| rs8126756 | 21 | 1.81401e-09 | C | T | 0.00061878584168435 | 36.0864061822702 | 0.2727 | C | T |
| rs1893592 | 21 | 1.48115e-13 | C | A | 0.000937120085919884 | 54.6684637630414 | 0.728501 | C | A |
| rs7278257 | 21 | 4.27504e-10 | C | G | 0.000670135275503143 | 39.0830150339214 | 0.601999 | C | G |
| rs2073609 | 21 | 1.46501e-08 | C | T | 0.000548151364659465 | 31.9648794273622 | 0.5822 | C | T |
| rs5754104 | 22 | 1.355e-10 | A | G | 0.000704482804846108 | 41.0876123484325 | 0.484299 | A | G |
| rs2069235 | 22 | 1.69005e-20 | A | G | 0.0014681367501917 | 85.6917532868614 | 0.1327 | A | G |
| rs4602367 | 3 | 1.75699e-10 | G | A | 0.000704523323802617 | 41.0899772051797 | 0.3936 | G | A |
| rs3806624 | 3 | 3.93641e-11 | G | A | 0.00074405731438719 | 43.3974385787144 | 0.2289 | G | A |
| rs13103285 | 4 | 4.29042e-14 | T | C | 0.00097695857072941 | 56.9947809589962 | 0.0304698 | T | C |
| rs34046593 | 4 | 7.16638e-17 | A | G | 0.00119903249995213 | 69.9659035544603 | 0.4558 | A | G |
| rs2918392 | 5 | 4.62104e-08 | C | T | 0.000514115359899186 | 29.9790841132627 | 0.584501 | C | T |
| rs7731626 | 5 | 1.94089e-26 | A | G | 0.00193513591072256 | 113.002265891452 | 0.5919 | A | G |
| rs403214 | 5 | 3.95804e-10 | G | A | 0.000671963065116686 | 39.1896853822411 | 0.4561 | G | A |
| rs244685 | 5 | 6.03601e-10 | G | T | 0.00065496959057473 | 38.1979561775948 | 0.0547003 | G | T |
| rs9405192 | 6 | 9.25977e-11 | A | G | 0.000723561044714213 | 42.2011198944327 | 0.1263 | A | G |
| rs12530098 | 6 | 1.35394e-11 | T | C | 0.000786800385694845 | 45.8924082435734 | 0.83 | T | C |
| rs3025669 | 6 | 2.91273e-30 | G | C | 0.00223042784521827 | 130.284385596443 | 0.9174 | G | C |
| rs1611236 | 6 | 4.5436e-19 | A | G | 0.00135509891890628 | 79.0850432483033 | 0.1675 | A | G |
| rs9258357 | 6 | 5.76501e-22 | C | T | 0.00159842263977987 | 93.3084145739876 | 0.0797609 | C | T |
| rs11754264 | 6 | 1.88105e-12 | C | T | 0.000849974017077673 | 49.5803276535847 | 0.6995 | C | T |
| rs112733823 | 6 | 3.82032e-24 | T | C | 0.00176780053508964 | 103.21341151019 | 0.8779 | T | C |
| rs139395255 | 6 | 8.6836e-60 | G | A | 0.00454375326935948 | 266.027792697619 | 0.6627 | G | A |
| rs114508013 | 6 | 1.81593e-28 | A | G | 0.00209653273183503 | 122.446834473192 | 0.4683 | A | G |
| rs115521560 | 6 | 1.29122e-103 | C | A | 0.0079726642579076 | 468.397191829171 | 0.2283 | C | A |
| rs71565312 | 6 | 1.1051e-67 | A | G | 0.00516066708279514 | 302.334245307225 | 0.3523 | A | G |
| rs146305655 | 6 | 3.29079e-22 | A | G | 0.00160777348524116 | 93.8551520918116 | 0.1968 | A | G |
| rs5020946 | 6 | 1e-200 | T | G | 0.0248937958134512 | 1487.89967838416 | 0.454 | T | G |
| rs9271365 | 6 | 1e-200 | G | T | 0.0244095978658332 | 1458.23511558168 | 0.669501 | G | T |
| rs2233424 | 6 | 6.49232e-26 | T | C | 0.0018889882274731 | 110.302371755295 | 0.3722 | T | C |
| rs76153210 | 6 | 6.83282e-15 | T | C | 0.00104016153387336 | 60.6858175702955 | 0.3249 | T | C |
| rs62422878 | 6 | 3.57396e-09 | T | C | 0.000595284016876986 | 34.7150083612477 | 0.1553 | T | C |
| rs7749323 | 6 | 3.46817e-29 | A | G | 0.0021497161530944 | 125.559674495087 | 0.6476 | A | G |
| rs212389 | 6 | 6.65733e-13 | A | G | 0.000887976559838028 | 51.7990461993269 | 0.1188 | A | G |
| rs1571878 | 6 | 4.12572e-40 | T | C | 0.00301094140472042 | 176.013653747781 | 0.8672 | T | C |
| rs740122 | 7 | 5.37205e-09 | A | G | 0.000583983887497936 | 34.0556368743678 | 0.3596 | A | G |
| rs42034 | 7 | 1.28499e-08 | G | A | 0.000555728515629577 | 32.4069788301643 | 0.5418 | G | A |
| rs3757387 | 7 | 1.86681e-19 | C | T | 0.00139457020037203 | 81.3918470625492 | 0.1431 | C | T |
| rs9693589 | 8 | 1.49589e-18 | A | G | 0.00132831561999551 | 77.5198618078778 | 0.9184 | A | G |
| rs11574914 | 9 | 9.91745e-15 | A | G | 0.00102633884407141 | 59.8785361777755 | 0.3108 | A | G |
| rs10435844 | 9 | 9.72971e-11 | T | G | 0.000719779256577692 | 41.9803912466634 | 0.4199 | T | G |

**Table S8. Results of heterogeneity test and pleiotropy test of instrumental variables**

| **Exposure** | **Outcomes** | **Heterogeneity** | | **Pleiotropy** | |
| --- | --- | --- | --- | --- | --- |
|  |  | **Q statistic (IVW)** | **P value** | **MR-Egger intercept** | **P value** |
| Rheumatoid arthritis | Neutrophil extracellular traps measurement | 72.028 | 0.411 | 0.043 | 0.205 |
|  | Interleukin-6 levels | 90.439 | 0.270 | 0 | 0.972 |
|  | Interleukin-18 levels | 78.844 | 0.609 | 0.001 | 0.752 |
|  | Interleukin-1β levels | 76.101 | 0.691 | 0.003 | 0.472 |
|  | TNF-a levels | 83.682 | 0.458 | 0.008 | 0.095 |
|  | Neutrophil count | 142.49 | <0.001 | 0 | 0.884 |
|  | Neutrophil count(Outlier corrected) | 95.499 | 0.002 | 0 | 0.936 |
|  | Interleukin-4 levels | 99.294 | 0.045 | 0.002 | 0.500 |
|  | Interleukin-5 levels | 94.038 | 0.191 | -0.004 | 0.492 |
|  | Interleukin-13 levels | 78.642 | 0.615 | -0.003 | 0.494 |
|  | Blood protein levels of myeloperoxidase | 86.753 | 0.130 | 0.004 | 0.393 |
|  | Blood protein levels of myeloperoxidase-DNA complexes | 86.294 | 0.137 | -0.008 | 0.141 |

**Table S9. Results of MR-PRESSO inspection**

| **Exposure** | **Outcomes** | **Raw** | | **Outlier corrected** | | **Global P** | **Number of outliers** | **Distortion P** |
| --- | --- | --- | --- | --- | --- | --- | --- | --- |
|  |  | OR (CI%) | **P** | **OR (CI%)** | **P** |  |  |  |
| Rheumatoid arthritis | Neutrophil extracellular traps measurement | 1.14 (0.90-1.45) | 0.27 | NA | NA | 0.415 | 0 | NA |
|  | Interleukin-6 levels | 1.02 (1.00-1.04) | 0.12 | NA | NA | 0.32 | 0 | NA |
|  | Interleukin-18 levels | 1.00 (0.96-1.03) | 0.83 | NA | NA | 0.321 | 0 | NA |
|  | Interleukin-1β levels | 1.02 (1.00-1.05) | 0.11 | NA | NA | 0.607 | 0 | NA |
|  | TNF-a levels | 1.04 (1.00-1.07) | 0.05 | NA | NA | 0.522 | 0 | NA |
|  | Neutrophil count | 1.01 (1.00-1.05) | 0.01 | 1.01 (1.00-1.01) | 0.02 | <0.001 | 3 | 0.397 |
|  | Neutrophil count(Outlier corrected) | 1.01 (1 - 1.01) | 0.02 | NA | NA | 0.001 | 0 | NA |
|  | Interleukin-4 levels | 1.01 (0.98-1.03) | 0.59 | NA | NA | 0.06 | 0 | NA |
|  | Interleukin-5 levels | 1.06 (1.02-1.10) | 0 | NA | NA | 0.226 | 0 | NA |
|  | Interleukin-13 levels | 1.06 (1.02-1.09) | 0 | NA | NA | 0.659 | 0 | NA |
|  | Blood protein levels of myeloperoxidase | 1.03 (1.00-1.01) | 0.04 | NA | NA | 0.165 | 0 | NA |
|  | Blood protein levels of myeloperoxidase-DNA complexes | 1.01 (0.97-1.05) | 0.57 | NA | NA | 0.151 | 0 | NA |
