# Supplementary material for: Causal association between neutrophil extracellular traps and rheumatoid arthritis: A 2-sample bidirectional Mendelian randomization study
Source: Medicine (Baltimore). 2025 Oct 31;104(44):e45331. doi: 10.1097/MD.0000000000045331 (PMC12582744; doi:10.1097/MD.0000000000045331)

**Supplemental Digital Content Figure 1.** Scatter plots of the Mendelian randomization analysis of rheumatoid arthritis (RA) as the outcome and (A) neutrophil extracellular traps (NETs), (B) interleukin (IL)-6, (C) IL-18, (D) IL-1β, (E) tumor necrosis factor (TNF)-α, (F) IL-5, (G) IL-5, (H) IL-13, (I) myeloperoxidase (MPO), and (J) MPO-DNA complexes as the exposures.


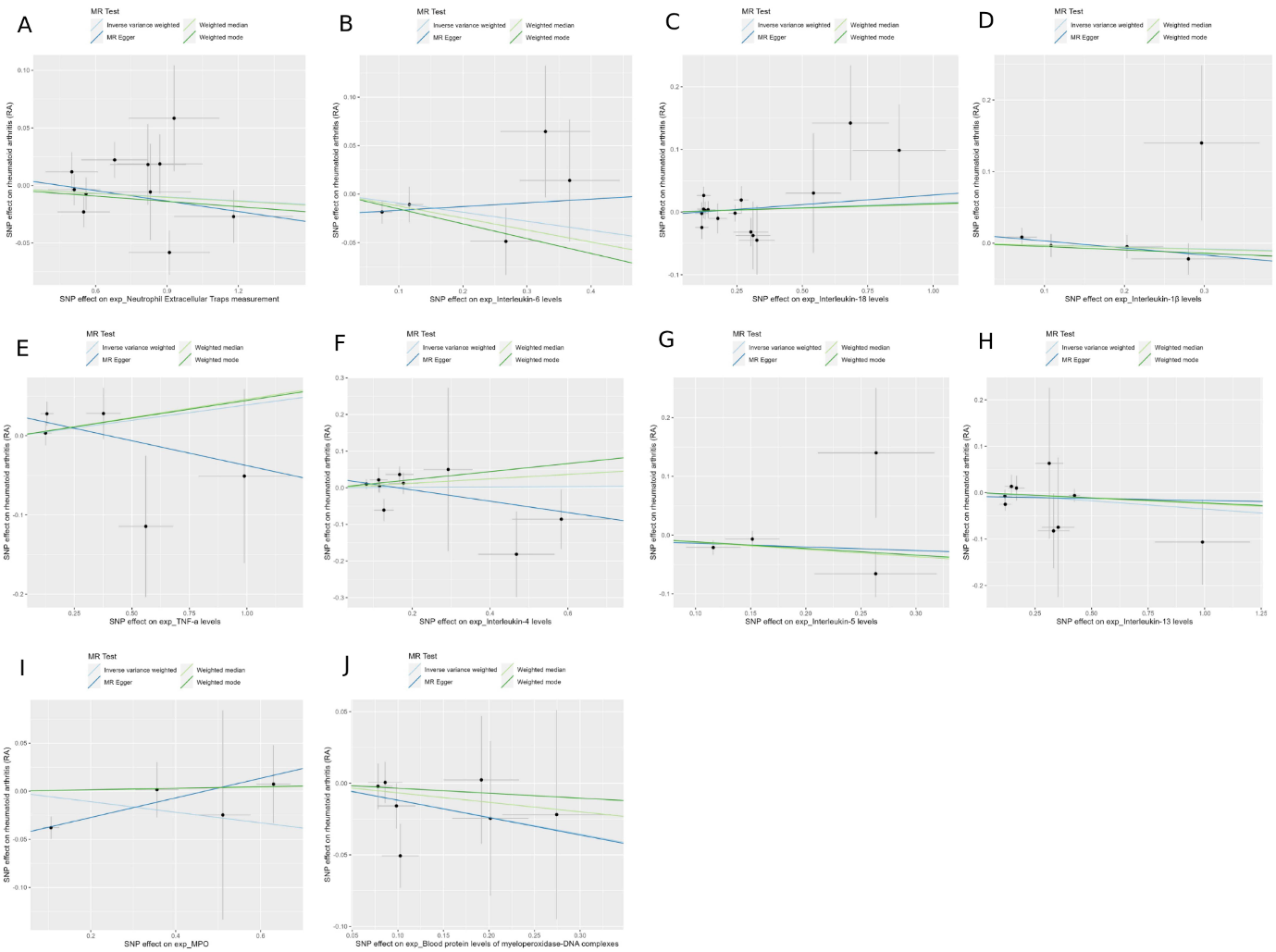


**Supplemental Digital Content Figure 2.** Forest plots of the Mendelian randomization analysis of rheumatoid arthritis (RA) as the outcome and (A) neutrophil extracellular traps (NETs), (B) interleukin (IL)-6, (C) IL-18, (D) IL-1β, (E) tumor necrosis factor (TNF)-α, (F) IL-5, (G) IL-5, (H) IL-13, (I) myeloperoxidase (MPO), and (J) MPO-DNA complexes as the exposures.


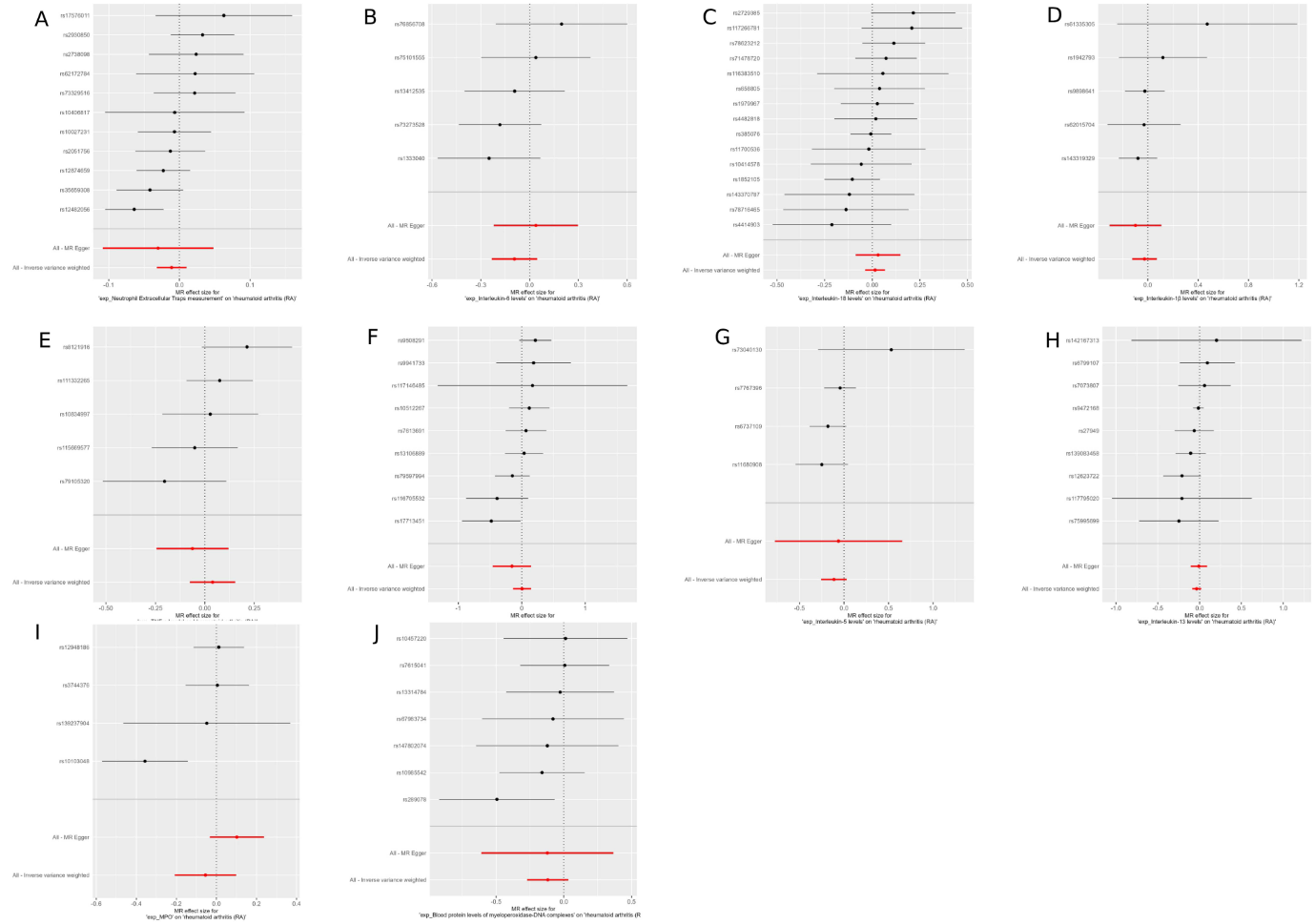


**Supplemental Digital Content Figure 3.** Funnel plots of the Mendelian randomization analysis of rheumatoid arthritis (RA) as the outcome and (A) neutrophil extracellular traps (NETs), (B) interleukin (IL)-6, (C) IL-18, (D) IL-1β, (E) tumor necrosis factor (TNF)-α, (F) IL-5, (G) IL-5, (H) IL-13, (I) myeloperoxidase (MPO), and (J) MPO-DNA complexes as the exposures.


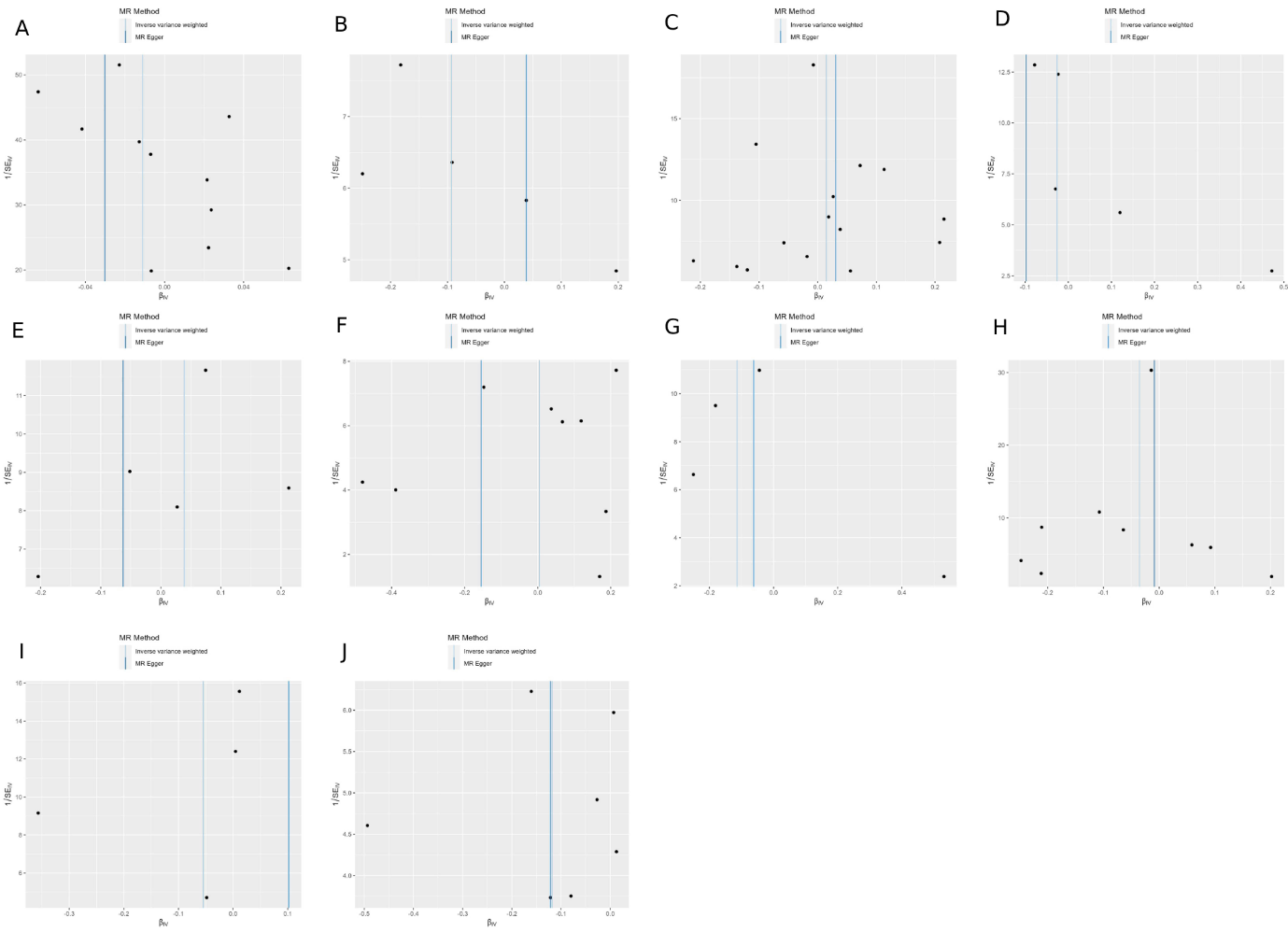


**Supplemental Digital Content Figure 4.** Leave-one-out forest plots of the Mendelian randomization analysis of rheumatoid arthritis (RA) as the outcome and (A) neutrophil extracellular traps (NETs), (B) interleukin (IL)-6, (C) IL-18, (D) IL-1β, (E) tumor necrosis factor (TNF)-α, (F) IL-5, (G) IL-5, (H) IL-13, (I) myeloperoxidase (MPO), and (J) MPO-DNA complexes as the exposures.


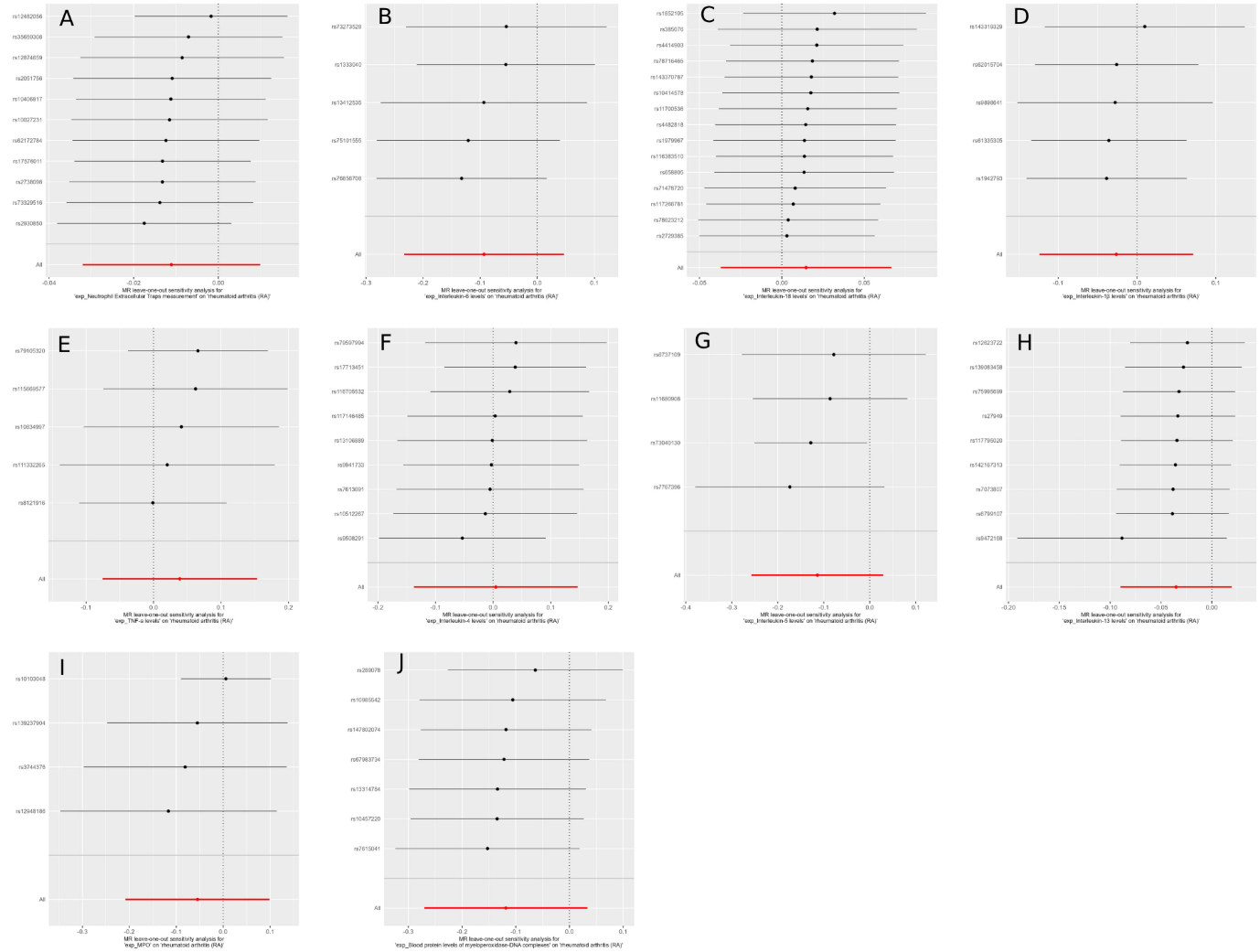


**Supplemental Digital Content Figure 5.** Scatter plots of the Mendelian randomization analysis of rheumatoid arthritis (RA) as the exposure and (A) neutrophil extracellular traps (NETs), (B) interleukin (IL)-6, (C) IL-18, (D) IL-1β, (E) IL-5, and (F) MPO-DNA complexes as the outcomes.


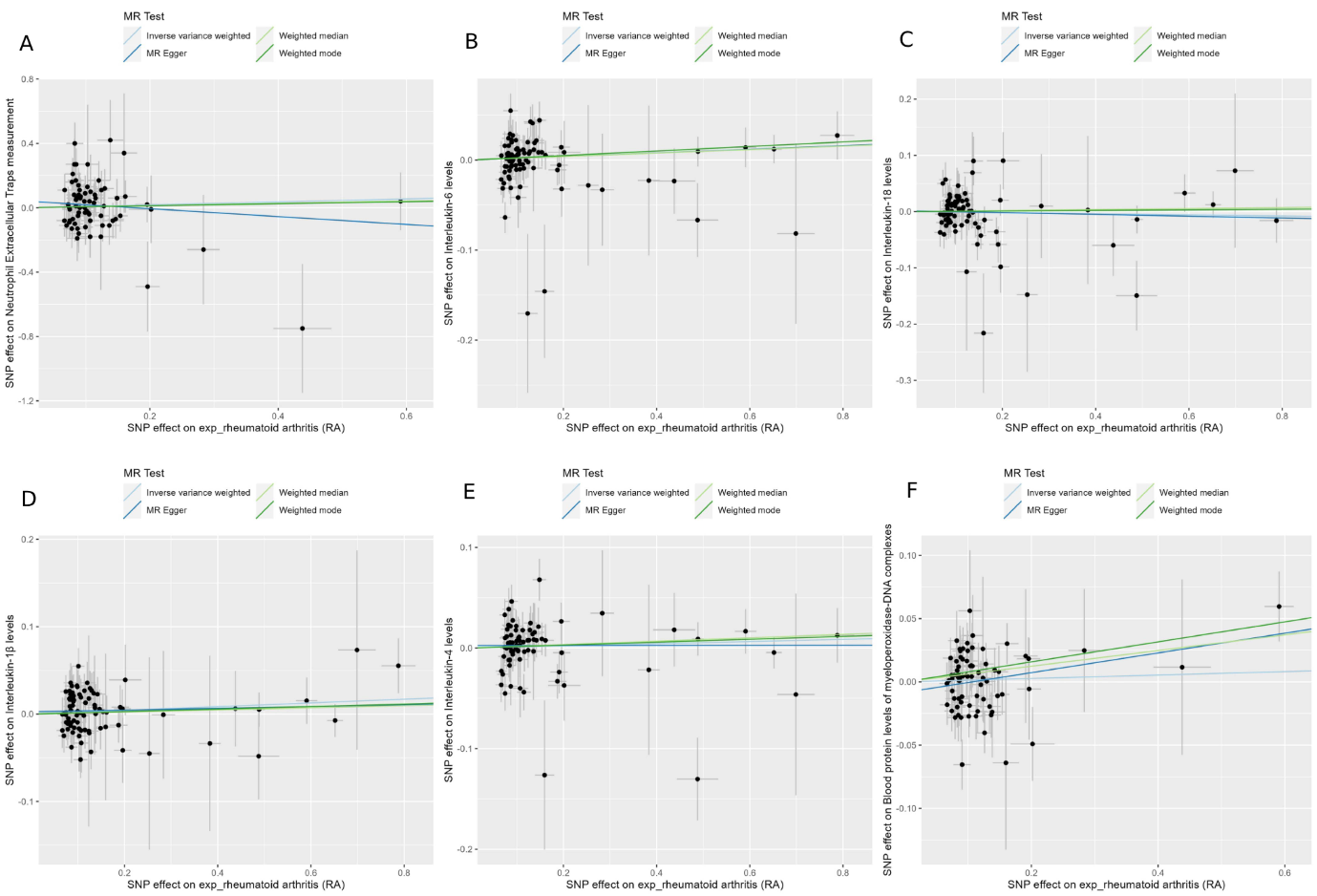


**Supplemental Digital Content Figure 6.** Forest plots of the Mendelian randomization analysis of rheumatoid arthritis (RA) as the exposure and (A) neutrophil extracellular traps (NETs), (B) interleukin (IL)-6, (C) IL-18, (D) IL-1β, (E) IL-5, and (F) MPO-DNA complexes as the outcomes.


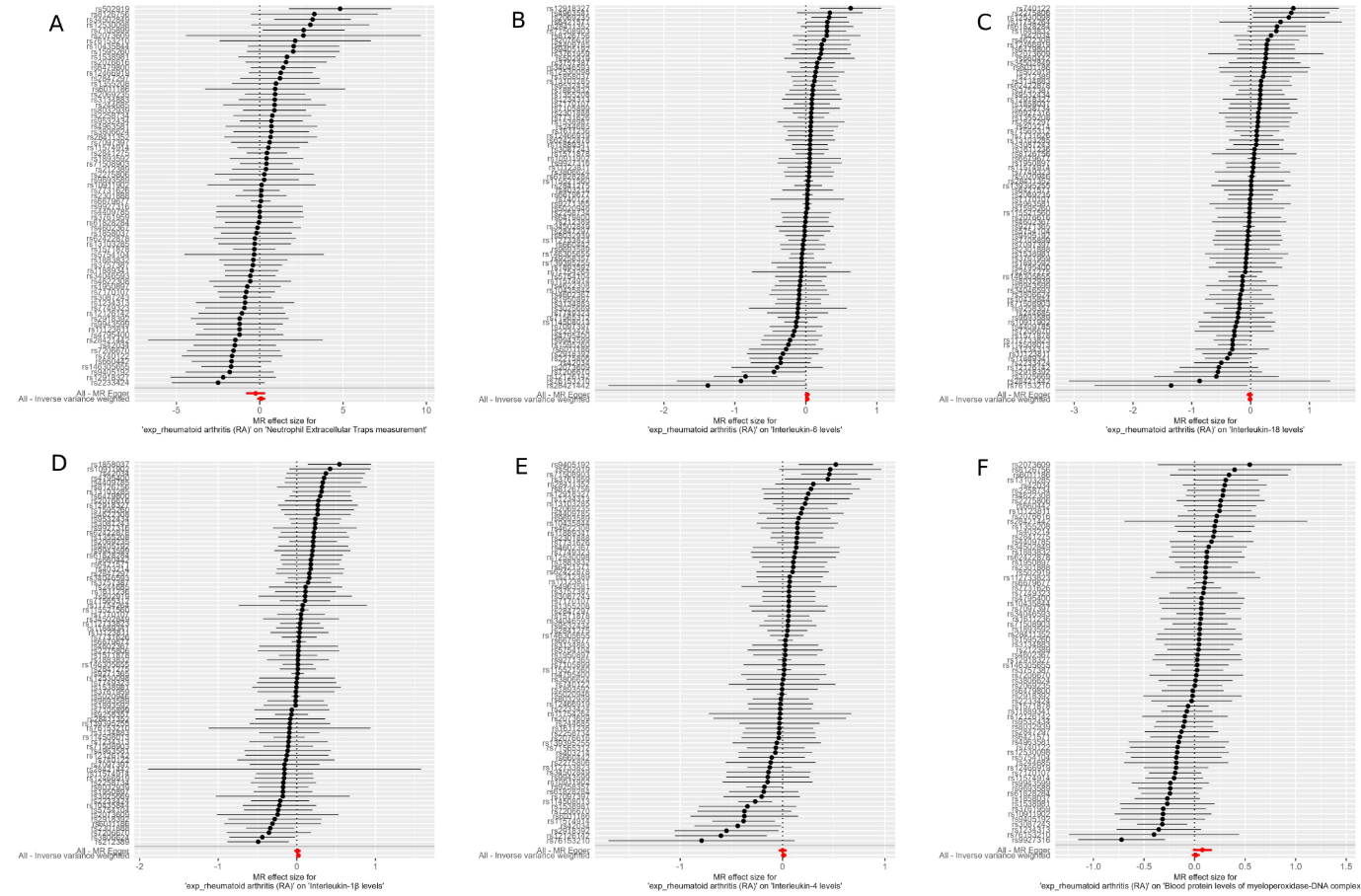


**Supplemental Digital Content Figure 7.** Funnel plots of the Mendelian randomization analysis of rheumatoid arthritis (RA) as the exposure and (A) neutrophil extracellular traps (NETs), (B) interleukin (IL)-6, (C) IL-18, (D) IL-1β, (E) IL-5, and (F) MPO-DNA complexes as the outcomes.


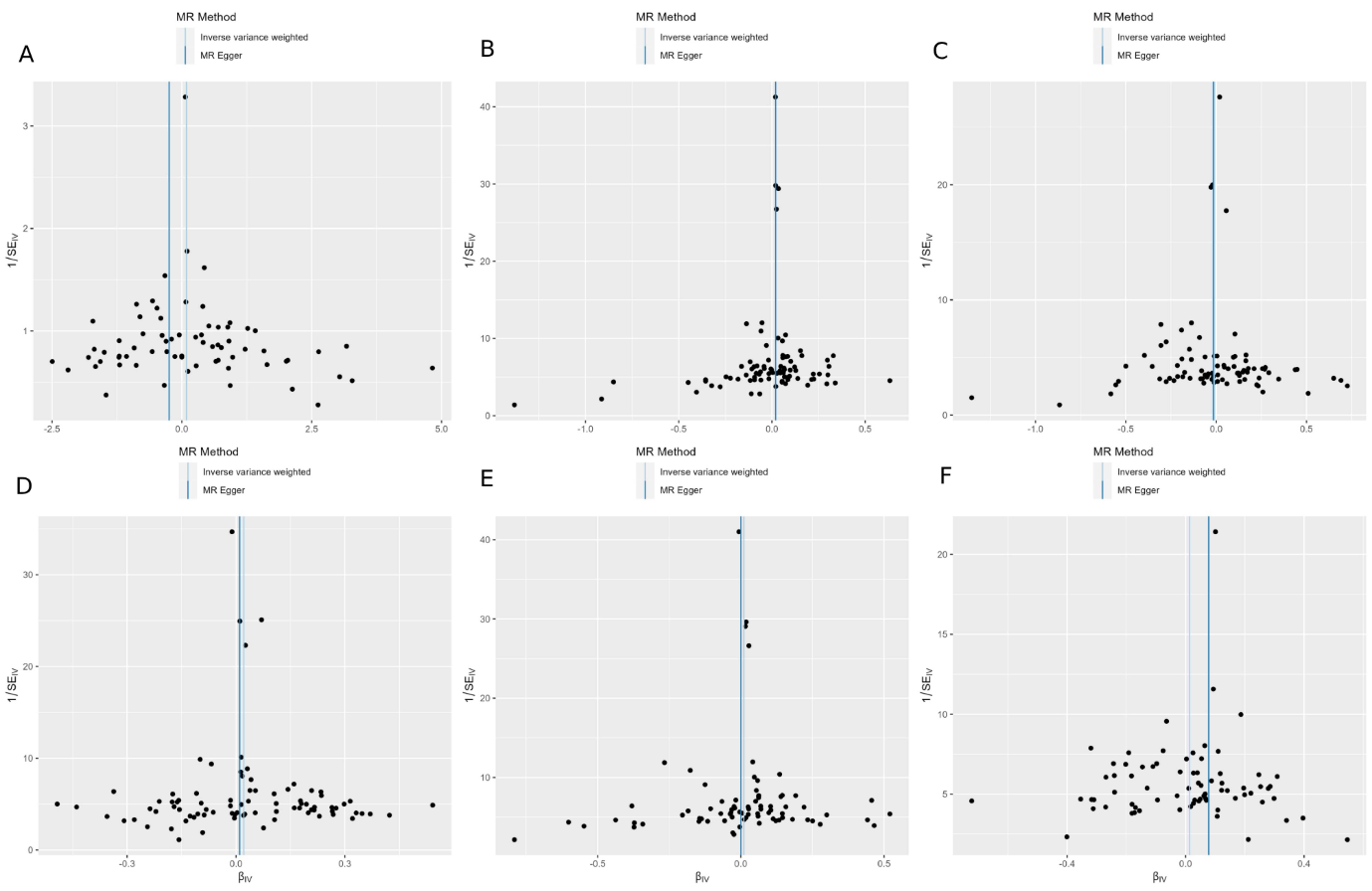


**Supplemental Digital Content Figure 8.** Leave-one-out forest plots of the Mendelian randomization analysis of rheumatoid arthritis (RA) as the exposure and (A) neutrophil extracellular traps (NETs), (B) interleukin (IL)-6, (C) IL-18, (D) IL-1β, (E) IL-5, and (F) MPO-DNA complexes as the outcomes.


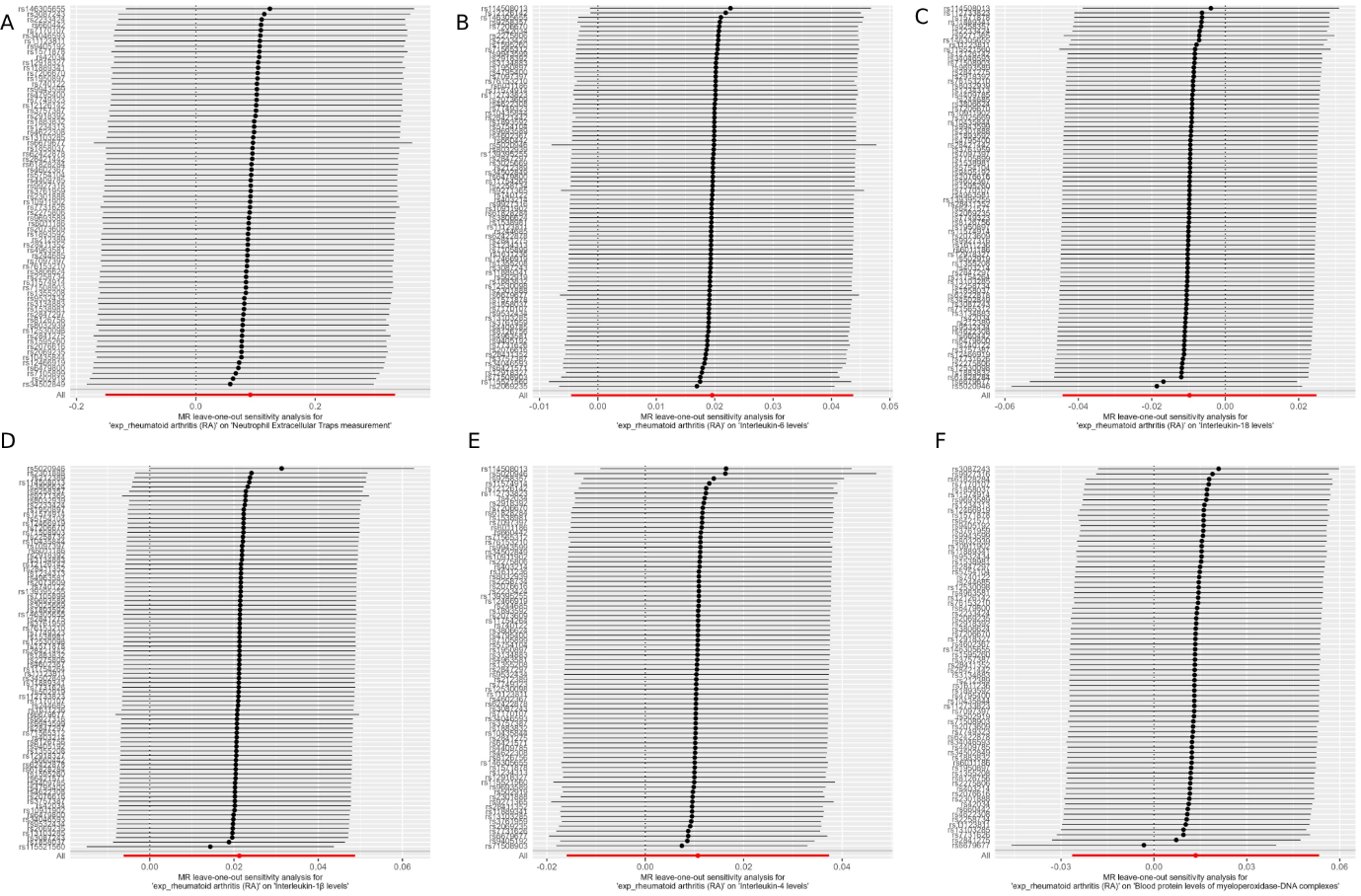

Supplement: Supplementary file 2 [file medi-104-e45331-s002.docx]
